# Exploring *Shigella* vaccine priorities and preferences: Results from a mixed-methods study in low- and middle-income settings

**DOI:** 10.1016/j.jvacx.2023.100368

**Published:** 2023-08-09

**Authors:** Jessica A. Fleming, Nikki Gurley, Sophia Knudson, Lassane Kabore, John Tanko Bawa, Patience Dapaah, Sandeep Kumar, Surendra Uranw, Thang Tran, Le Thi Phuong Mai, Chris Odero, Christopher Obong'o, Kofi Aburam, Stella Wanjiru, Nguyen Thi My Hanh, Luu Phuong Dung, William P. Hausdorff

**Affiliations:** a PATH, Seattle, 2201 Westlake Ave, Seattle, WA 98121, USA; b PATH, Senegal, Fann Résidence, Rue Saint John Perse X F, Dakar, Senegal; c PATH, Ghana, PMB CT 307 Cantonments, Accra, Ghana; d PATH, India, 15th Floor, Dr. Gopal Das Bhawan 28, Barakhamba Road, Connaught Place, New Delhi 110001, India; e B.P. Koirala Institute of Health Sciences, Buddha Road, Dharan 56700, Nepal; f PATH, Viet Nam, #1101, 11th Floor, Hanoi Towers, 49 Hai Ba Trung, Hoan Kiem District, Hanoi, Viet Nam; g National Institute of Hygiene & Epidemiology, 1 P. Yec Xanh, Phạm Đình Hổ, Hai Bà Trưng, Hà Nội 100000, Viet Nam; h PATH, Kenya, ACS Plaza, 4th Floor Lenana and Galana Road, P.O. Box 76634-00508, Nairobi, Kenya; i PATH, Washington, DC, 455 Massachusetts Ave. NW, Suite 1000, Washington, DC 20001, USA

**Keywords:** *Shigella*, Vaccine, Feasibility, Acceptability, Vaccine decision-making

## Abstract

**Background:**

*Shigella* is the leading bacterial cause of diarrheal mortality in children and can cause long-term effects on growth and development. No licensed *Shigella* vaccines currently exist but several promising candidates are in development and could be available in the next five years. Despite *Shigella* being a well-known public health target of the World Health Organization for decades, given current burden estimates and competing preventable disease priorities in low-income settings, whether the availability of an effective *Shigella* vaccine will lead to its prioritization and widespread introduction among countries at highest risk is unknown.

**Methods:**

We conducted a mixed-methods study of national stakeholders and healthcare providers in five countries in Asia and Africa and regional stakeholders in the Pan American Health Organization to identify preferences and priorities for forthcoming *Shigella* vaccines.

**Results:**

In our study of 89 individuals, diarrhea was the most frequently mentioned serious health concern for children under five years. Antimicrobial resistance (AMR) was more often considered very concerning than diarrhea or stunting. *Shigella* awareness was high but not considered a serious health concern by most stakeholders. Most participants were willing to consider adding a new vaccine to the routine immunization schedule but expressed reservations about a *Shigella* vaccine because of lower perceived burden relative to other preventable diseases and an already crowded schedule; interest was highest among national stakeholders in countries receiving more financial support for immunization. The priority of a *Shigella* vaccine rose when participants considered vaccine impacts on reducing stunting and AMR. Participants strongly preferred oral and combination vaccines compared to injectable and a single-antigen presentations, citing greater perceived community acceptability.

**Conclusions:**

This study provides a critical opportunity to hear directly from country and regional stakeholders about health priorities and preferences around new vaccines. These findings should inform ongoing *Shigella* vaccine development efforts and eventual vaccine introduction and implementation planning.

## Highlights

• High *Shigella* awareness but low vaccine interest due to low perceived burden and crowded schedule.
• *Shigella* vaccine priority increased if impacts on stunting and antimicrobial resistance included.
• Strong stakeholder preference for oral and combination vaccine formulations.

## 1. Background

*Shigella* is a gram-negative bacterium that is spread fecal-orally or through contaminated food and water. A cause of moderate to severe diarrhea, it is often associated with severe or bloody diarrhea, or dysentery. *Shigella* has long been considered a leading bacterial cause of diarrheal disease in low- and middle-income countries (LMICs), where the vast majority of cases occur [1], [2]. In the early 1990s, WHO estimated that half a million pediatric deaths were attributed to *Shigella* annually and the development of a safe and effective vaccine was a major public health priority [3], [4]. Over the intervening decades, however, the epidemiological portrait of *Shigella* has changed. Refined models now estimate that *Shigella* is responsible for 42,000–94,000 deaths of children less than five each year, still making it the largest bacterial cause of diarrheal disease mortality in that age group, but reducing it to moderate importance in global child survival compared to some other existing or likely forthcoming vaccine-preventable pathogens (e.g., malaria, tuberculosis, respiratory syncytial virus) [1], [5], [6]. These lower mortality values likely also reflect, in addition to enhancements in etiological determination, a confluence of environmental improvements affecting diarrheal deaths as a whole, including improved nutritional status and access to treatment [6]. However, with the emergence of increasingly sensitive molecular diagnostic tools, *Shigella* is now recognized to significantly contribute to the burden of non-bloody diarrhea and is associated with other negative health outcomes among children in LMICs, including stunting, severe malnutrition, metabolic disorders, and increased mortality from other infectious diseases [7], [8], [9], [10], [11], [12], [13], [14], [15], [16]. There is also evidence that since *Shigella* diarrhea is not always bloody, misdiagnosis and inappropriate administration of antibiotics for *Shigella* occurs frequently, leading to increasing antimicrobial resistance (AMR) to *Shigella* [17], [18], [19].

Vaccines against *Shigella* are currently in development and may be available by 2027; the most advanced candidates are injectable and will likely require one or two doses given between the ages of six months and one year [20]. They will enter an already crowded market and vaccination schedule, competing for space among vaccines targeting major enteric and other vaccine-preventable child illnesses.

Historically, vaccine prioritization is seldom informed by systematic surveys of those most directly involved in introduction decision-making and implementation at the country level. In 2020–2022, PATH undertook several studies, analyses, and expert convenings to address several key information gaps in defining a full *Shigella* vaccine value proposition. These included examining the strength of the association between *Shigella* infection, disease, and stunting; estimating the health impact and cost-effectiveness of potential *Shigella* vaccines; forecasting the potential demand for a *Shigella* vaccine in LMICs and among traveler and military populations from high-income countries; identifying design priorities, clinical testing, and regulatory, policy, and financial challenges to developing a *Shigella*-containing combination vaccine; and assessing knowledge and preferences of country and regional stakeholders around *Shigella* disease and vaccine priorities [21], [22], [23], [24]. This paper describes the findings from the country and regional stakeholder study. Considered together with the other evidence generated for the value proposition, this provides important data to inform ongoing vaccine development efforts, support vaccine introduction decision-making in LMICs, and help guide implementation planning.

## 2. Methods

We used mixed methods to explore factors that underlie the value of, and may shape future demand for, a *Shigella* vaccine among stakeholders in Burkina Faso, Ghana, Kenya, Nepal, and Vietnam, and the World Health Organization (WHO)’s Pan-American Health Organization (PAHO) regional headquarters in Washington, DC, USA.

### 2.1 Country and participant selection

Countries meeting the World Bank’s criterion for low- or lower-middle income with a PATH office or an existing relationship with relevant research partners were eligible for selection. Prioritization criteria included the availability of diarrhea burden data and country experience with diarrheal vaccine(s); countries were selected to maximize diversity of *Shigella* burden, stunting prevalence, and Gavi financing eligibility. We purposefully selected national- and regional-level participants who had authority over vaccine introduction decisions in their relevant geography; this included members of the Ministry of Health, the National Immunization Technical Advisory Group (NITAG), or those working in public health diarrhea control, nutrition, or vaccination. Healthcare providers from primary, secondary, or tertiary level facilities were either the head of their respective facility or worked in immunization at one of four to five randomly selected health facilities providing immunization within a day’s drive of each country capital.

### 2.3 Ethics

The study received exempt status from WIRB-Copernicus Group Institutional Review Board (IRB) (Olympia, WA, USA) and country approvals from Burkina Faso’s Comité d'Ethique pour la Recherche en Santé, the Ghana Health Service Ethics Review Committee, the Kenyatta National Hospital-University of Nairobi Research Ethics Committee, the Nepal Health Research Council and B.P. Koirala Institute of Health Sciences in Dharan, Nepal, and Vietnam’s National Institute of Hygiene & Epidemiology IRB. Informed consent was obtained from each participant prior to interviews.

### 2.4 Data collection

Audio-recorded, semi-structured interviews were conducted from October 2021 to April 2022 in English, French, Nepali, or Vietnamese, either in-person or virtually, collecting quantitative data on fixed-choice questions and qualitative descriptions on open-ended questions and follow-up probes (Supplemental material 1). To identify health concerns, participants were asked to list two or three of the most important health or development concerns for children under five years. They were then asked to rate the importance of diarrhea, growth stunting, and AMR as “a very serious health concern,” “serious, but not among the top health concerns,” or “not serious compared to other health concerns.” To explore vaccine impact thresholds and the priority of *Shigella* in vaccine introduction, we employed an iterative interview process in which we provided progressively more information about under-five year global- and country-specific *Shigella* diarrhea burden, deaths, sequelae, and potential vaccine impact to participants (Table 1) [8], [25], [26]. Participants rated the priority of introducing a *Shigella* vaccine in their country after hearing each new level of information.

**Table 1:** Information provided to participants by iterative interview level.^1^, ^2^

Level	Information categories	Annual numbers under five-years and information shared				
		Burkina Faso	Ghana	Kenya	Nepal	Vietnam
	N/A	No information given				
	Global *Shigella* diarrhea cases Global *Shigella* deaths	75 million cases 64,000 deaths				
	*Shigella* diarrhea cases	95,400	115,500	209,000	48,400	6,800
	*Shigella* diarrhea cases averted with 60% effective vaccine	52,075	67,231	115,342	27,008	3,925
	*Shigella* deaths	530	220	590	85	5
	*Shigella* deaths averted with 60% effective vaccine	289	124	315	44	3
	Antibiotic resistance	Vaccine has ability to slow pace or prevent antibiotic resistance for *Shigella*				
	Stunting cases from *Shigella*	15,400	12,700	28,800	9200	900
	Stunting cases from *Shigella* averted with 60% effective vaccine	8400	7000	15,400	4800	530

1 Data provided to participants at each stage came from the linked sources [8], [25], [26].

2 Information was provided progressively, where the participant gained more information at each level to build off the information from the previous level.

Stakeholders asked questions at Level 1 received no prior background information (Table 1). For Levels 2–4, participants were asked to assume the following vaccine characteristics based on the World Health Organization’s *Shigella* vaccine preferred product characteristics [20] and the Bill & Melinda Gates Foundation’s investigational target product profile (iTPP) for *Shigella* vaccines:

• Vaccine availability 2025–2030
• Injectable presentation
• One- or two-dose schedule given mid-to-late in the first year of life
• 60% vaccine efficacy

A cost estimate of approximately US$1/dose was used based on the LMIC price of typhoid conjugate vaccine [27] and the potential for funding support through Gavi, the Vaccine Alliance, if eligible.

Participants were also asked about their willingness to accept various vaccine attributes through fixed-choice questions modeled on discrete choice surveys, with accompanying open-ended questions to gather insights on attribute preference.

### 2.5 Data analysis

#### 2.5.1 Data processing

Interviewers used iPads to conduct and record interviews and capture open-ended responses. Responses to fixed-choice questions were automatically uploaded to datafiles using Research Electronic Data Capture (REDCap) tools on the iPad [28]. Audio recordings of interviews were transcribed in full; those conducted in local languages were first translated into English. Transcripts were coded using NVivo 12 Pro [29]. For quality assurance, we reviewed transcripts alongside the quantitative data files and aligned discrepancies to the responses recorded in the transcript.

#### 2.5.2 Data analysis

Interview transcripts and quantitative data were analyzed independently and together using mixed-method analytic approaches. A team of three investigators (NG, SK, JAF) coded interview transcripts using a deductively- and inductively developed codebook. Coding themes included perceptions of disease burden, barriers and benefits of a *Shigella*-containing vaccine, and operational considerations for vaccine introduction. The coding team met regularly to validate and refine coding. Codes were matrixed in NVivo and exported to Excel where they were integrated with quantitative data. Initial codes were reviewed in Excel and compressed into broader themes based on observed patterns. Data was subsequently visualized using Tableau Desktop 2021.4 to identify thematic patterns by stakeholder type, country, and across the study population.

Results present frequency distributions for fixed-choice questions alongside the qualitative findings detailing the rationale behind their selection. Findings are pooled across countries and stratified by participant type unless otherwise noted (country-specific figures are provided as Supplemental material); results from PAHO interviews are described separately.

## 3 Results

### 3.1 Sample

In each country, 10–13 healthcare providers and 5–9 national stakeholders were interviewed (Table 2). With one exception, healthcare providers worked in public health facilities.

**Table 2:** National Stakeholders and Health Providers by Country and Role.

Country	National stakeholders		Health providers			
	Total	Member of NITAG^1^	Total	Health facility level		
				Primary	Secondary	Tertiary
**Burkina Faso**	7	3	13	13^2^	0	0
**Ghana**	6	4	11	8	3	0
**Kenya**	5	5	10	5	3	2
**Nepal**	5	3	10	5	2	3
**Vietnam**	9	5	10	4	4	2
***Total***	*32*	*20*	*54*	*35*	*12*	*7*

1 NITAG-National immunization technical advisory group.

2 Immunizations only provided at primary health level.

All healthcare providers from Burkina Faso worked at the primary health level while 46% of providers from the other countries worked at secondary or tertiary health facilities. Of national stakeholders, 63% were current members of National Immunization Technical Advisory Groups (NITAGs) or equivalent vaccine decision-making bodies. Three regional stakeholders from PAHO were also interviewed.

### 3.2 Health concerns

**Fig. 1:**
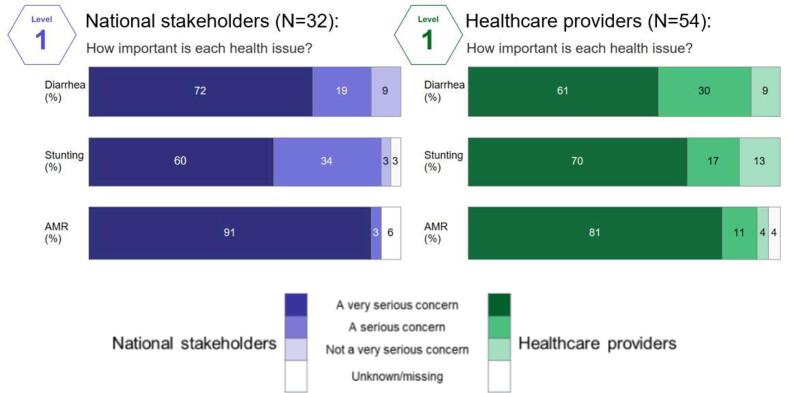
Importance of given health issues, by stakeholder group.

When provided no background information on *Shigella* burden or expected vaccine impact (Level 1), providers were first asked to list the two or three most important child health issues in their country. In response to this open-ended question, the most frequently mentioned health concern for children under five years across countries was diarrhea (n = 62/86), followed by respiratory infections (n = 49/86), malnutrition/anemia (n = 36/86), and vaccine preventable diseases (n = 26/86). African participants also cited malaria (n = 23/52). When subsequently asked to rate the importance of diarrhea, stunting, and AMR as a health concern, participants across countries rated AMR a more serious concern than both diarrhea and stunting, with more than 80% of both national stakeholders and healthcare providers rating AMR a “very serious concern” (Fig. 1). In four of the five countries, national stakeholders consistently rated AMR a “very serious concern” and considered it more serious than healthcare providers; Kenya was the outlier, where AMR was considered less serious across both groups (Supplemental material 2). Diarrhea was rated a “very serious concern” among both national stakeholders and healthcare providers in Burkina Faso, Ghana, and Kenya, while it was less of a concern among healthcare providers in Nepal and across both groups in Vietnam. Stunting was considered a “very serious concern” across both groups in Ghana and among healthcare providers in Burkina Faso and Nepal.

Two themes emerged from the most frequently cited rationales behind the ratings: the impact on an individual’s short- or long-term health and the quality and/or availability of health care or diagnostics (Table 3). Diarrhea was described as a “very serious concern” primarily due to its acute health impacts, specifically mortality, while the reasons for rating stunting as such were predominantly attributed to downstream health impacts such as an increased vulnerability to other diseases or the impact of irreversible sequelae:

*“It is a very serious problem because it has lifelong impact. It impairs the growth as well as the development of the child.” - A health provider in Nepal*

**Table 3:** Most frequently cited reasons for rating health issue a very serious concern by reason category and stakeholder group1.

		Health issue					
		Diarrhea		Stunting		AMR^2^	
		Number of respondents rating health issue as a very serious concern					
		NS^3^	HP^4^	NS^3^	HP^4^	NS^3^	HP^4^
		n = 23	n = 33	n = 19	n = 38	n = 29	n = 44
Category	Reason	Number of respondents mentioning cited reason					
Health impact	High mortality	16	22	1	1	0	0
	Creates vulnerability to other diseases	7	6	3	12	0	0
	Irreversible sequelae / health impacts	0	0	9	9	0	0
Quality of care	Poor treatment seeking or treatment adherence behavior	1	6	0	0	1	11
	Inadequate tools for diagnosis or management of condition	1	4	0	0	3	7
	Poorly regulated clinical care and over-prescription of drugs	0	0	0	0	18	21

1 Note: reasons are coded from qualitative data and respondents were able to provide multiple reasons for their selection. As a result, sum of numbers in columns may exceed the total number of respondents.

2 AMR-Antimicrobial resistance.

3 NS-National stakeholders.

4 HP-Health provider.

Participants attributed their concern about AMR to issues related to the quality of health care, including the inability to adequately diagnose pathogens or monitor AMR; poor public adherence to antibiotic regimens; or weak country regulations, leading to the over-prescription and over-use of antibiotics. One participant articulated how these quality-of-care factors are interrelated:

*“Now there are a lot of pharmacies, people can buy antibiotic … without a doctor’s prescription. If the symptoms are controlled, the patient stops on their own [before finishing the treatment course], so there is a high potential for antibiotic resistance.” - A health provider in Vietnam*

Across all health issues, healthcare providers more frequently cited concerns around challenges in delivering quality care than did national stakeholders (49/54 vs 24/32, respectively).

Similar to country results, PAHO respondents listed diarrhea, respiratory diseases, and malnutrition as the top three health concerns in Latin America. When probed specifically about AMR, diarrhea, and stunting, PAHO respondents unanimously agreed that AMR was the priority issue, followed by diarrhea, and then stunting. Reasons cited for high prioritization of AMR included the challenges of treating other top health concerns, and the difficulty of treating Shigella specifically, as described by one stakeholder:

*“There are two antibiotics that are mainly used to control or cure diarrhea by Shigella, you have to be concerned because both… may evolve to turn resistant.” – A PAHO stakeholder*

The primary concerns cited with stunting were lack of government programs to address stunting and the variability of stunting burden across the PAHO region.

### 3.3 *Shigella* as a health concern

**Fig. 2:**
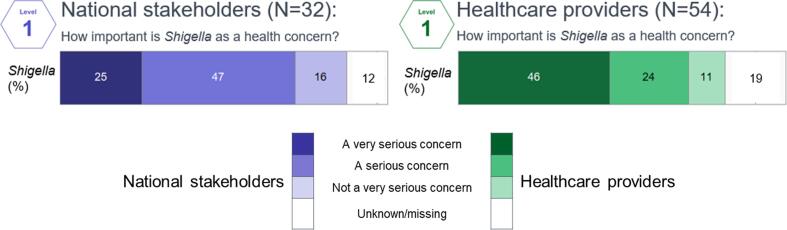
Importance of *Shigella*, by stakeholder group.

Provided no prior information, awareness of *Shigella* was high among participants (n = 81; 93%), however, less than half (46%) of healthcare providers and one quarter (25%) of national stakeholders rated *Shigella* as a “very serious concern” (Fig. 2). *Shigella* was more likely to be considered a “very serious concern” by both national stakeholders and healthcare providers in Burkina Faso and Ghana compared to other countries (Supplemental material 3).

For participants rating *Shigella* as “not a very serious concern,” reasons included the lower prevalence of *Shigella* compared to other diarrheal pathogens and other vaccine-preventable diseases; already having evidence of a reduction in diarrhea disease burden after the introduction of rotavirus vaccine; or *Shigella* not being the only—or even primary—pathogen to cause the given health issues. Often these reasons overlapped, as articulated by a national stakeholder:

*“It’s fairly low priority because we have other diseases that cause more of the [diarrhea] infections, […] things like typhoid. We’ve [already] introduced the Rotavirus vaccine.” - A national stakeholder in Kenya*

Participants rating *Shigella* a “serious” or “very serious” concern, particularly healthcare providers, often attributed its importance to challenges in diagnosing, treating, and preventing *Shigella*:

*“Shigella management is not typical. You have to treat it by trial and error.” - A health provider in Burkina Faso*

Across all countries, stakeholders expressed the need for local data on *Shigella* burden and improved availability of diagnostic and surveillance tools.

### 3.4 Prioritization of a *Shigella* vaccine

Participants typically had a positive perception of vaccines and were willing to consider adding new vaccines to the routine immunization schedule, citing perceived health benefits of immunization. However, decisions to introduce a new vaccine were described as requiring careful consideration of both the benefits and risks:

*“We really need to have a disease burden otherwise we cannot say there is a space for EPI introduction or not.” - A national stakeholder in Vietnam*

**Fig. 3:**
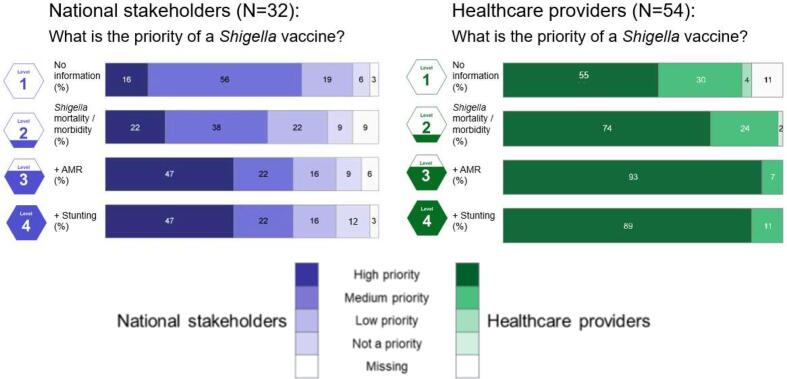
*Shigella* vaccine prioritization across scenarios of estimated vaccine impact on disease burden and sequelae, by stakeholder group (%).

When asked to prioritize the introduction of a hypothetical *Shigella* vaccine given no background information, 16% of national stakeholders and 55% of healthcare providers gave it “high priority” (Fig. 3, Level 1, top bar graph). *Shigella* vaccine prioritization in all countries combined increased among both national stakeholders and healthcare providers when provided information on global disease burden, hypothetical vaccine characteristics, and estimated country-specific reductions of *Shigella* diarrhea burden (Fig. 3, Level 2). By country, 86% and 100% of national stakeholders in Burkina Faso and Nepal, respectively, prioritized a *Shigella* vaccine as “medium priority” or “high priority” compared to 60% and 67% in Kenya and Ghana, respectively, and 11% in Vietnam (Supplemental material 4, Level 2). Across all countries combined, prioritization most notably increased when participants additionally received information on AMR and stunting; nearly half of national stakeholders and around 90% of healthcare providers rated a *Shigella* vaccine as “high priority” (Fig. 3, Levels 3 and 4). All healthcare providers rated a *Shigella* vaccine as “high priority” in Burkina Faso, Ghana, Kenya, and Nepal when provided information of vaccine impact on AMR (Supplemental material 4, Level 3). Across all levels of information provided and countries combined, roughly 40% fewer national stakeholders than healthcare providers cited a *Shigella* vaccine as “high priority.” Reflecting this lower prioritization, around a quarter of national stakeholders consistently rated a *Shigella* vaccine as “low priority” or “not a priority,” regardless of additional vaccine benefits.

Reflecting earlier themes around the importance of disease severity, the most frequently cited reasons for rating a *Shigella* vaccine as “high priority” were the perceived benefit of a *Shigella* vaccine on an individual’s short- or long-term health or the ability to improve the quality of care (Table 4). When presented with information on diarrhea morbidity and mortality, participants who rated a *Shigella* vaccine as “high priority” were concerned primarily with reducing the burden of acute health impacts (e.g., diarrhea) and averting mortality. When presented with information on stunting, participants who rated a *Shigella* vaccine as “high priority” noted the reduction in burden of stunting, malnutrition, and anemia cases, but also highlighted the ability of the vaccine to alleviate downstream health impacts, sequelae, and their social consequences:

*“The effect is beyond just diarrhea.…The policy will be to ensure that these children are spared this loss of cognitive and physical impairments so that they can attain their full potential.” - A national stakeholder in Ghana*

**Table 4:** Benefits cited as a reason for ranking a *Shigella* vaccine a “high priority” by levels of information provided, category, and stakeholder group.

		Level of information provided							
		No information		Diarrhea		AMR^1^		Stunting	
		Number of respondents citing a *Shigella* vaccine as high priority							
		NS^2^	HP^3^	NS^2^	HP^3^	NS^2^	HP^3^	NS^2^	HP^3^
		n = 5	n = 30	n = 7	n = 40	n = 15	n = 50	n = 15	n = 48
Category	Reason	Number of respondents mentioning cited reason							
Health impact	Potential *Shigella* vaccine has high efficacy	1	–	2	2	–	2	–	–
	Reduces mortality or “saves lives” of children	1	3	4	22	3	5	4	1
	Reduces high burden of disease (“fewer cases”) of the mentioned condition	2	4	2	19	10	17	5	15
	Alleviates downstream negative impacts, including sequelae and social impacts	–	1	2	1	1	–	2	14
	Multiple health benefits of a vaccine (e.g. treats multiple conditions)	–	–	–	–	–	3	2	8
Quality of care	Improved tool for preventing or treating the mentioned condition	–	–	–	–	2	7	–	3

Note: reasons are coded from qualitative data and respondents were able to provide multiple reasons for their selection. As a result, sum of numbers in columns may exceed the total number of respondents.

1 AMR-Antimicrobial resistance.

2 NS-National stakeholders.

3 HP-Health provider.

When given information on AMR, participants attributed high prioritization of a *Shigella* vaccine to the potential ability of the vaccine to both reduce the number of cases of antibiotic resistant *Shigella* and address quality of care concerns by preventing development of AMR in the first place:

*“Better to have a vaccine than to have antibiotics with doubtful performance. Anti-microbials are always prone to develop antimicrobial resistance.” - A health provider in Nepal*

Participants rating a *Shigella* vaccine highly when given information about stunting or AMR also cited the benefit of the vaccine protecting against multiple conditions, describing the benefit succinctly:

*“The vaccine is going to prevent two diseases at a go.” - A health provider in Ghana*

Across all health issues, healthcare providers were slightly more likely than national stakeholders to situate the benefit of a *Shigella* vaccine around its ability to provide better quality health care.

Conversely, participants rating a *Shigella* vaccine “low priority” or “not a priority” often cited concern over the lack of expected vaccine impact, noting that the burden of disease caused by *Shigella* was “not large”, or the “numbers do not speak” (Table 5). They also cited competition from other health priorities, the existence of other diarrhea prevention tools, and that *Shigella* is not the only contributing pathogen related to the given health issue:

*“Stunting is not only because of Shigella. […] There are multifactorial causes of stunting.” - A health provider in Nepal*

**Table 5:** Barriers cited as a reason for ranking a *Shigella* vaccine a “low priority” or “not a priority” by levels of information provided, category, and stakeholder group.

		Level of information provided							
		No information		Diarrhea		AMR^1^		Stunting	
		Number of respondents citing a *Shigella* vaccine as low or not a priority							
		NS^2^	HP^3^	NS^2^	HP^3^	NS^2^	HP^3^	NS^2^	HP^3^
		n = 8	n = 2	n = 10	n = 1	n = 8	n = 0	n = 9	n = 0
Category	Reason	Number of respondents mentioning cited reason							
Vaccines have inadequate impact	Perceived low prevalence of *Shigella*	2	–	8	–	2	–	5	–
	Disease burden has already been reduced	–	–	–	–	–	–	1	–
	Inadequate impact of vaccine	–	–	2	–	1	–	–	–
	*Shigella* is not the only pathogen contributing to health issue	2	–	2	–	3	–	1	–
Other priorities	Non-vaccine preventative measures available	2	–	2	–	–	–	1	–
	More important disease priorities	2	–	4	–	1	–	–	–
Inadequate data available on *Shigella*		2	–	0	–	2	–	2	–

Note: reasons are coded from qualitative data and respondents were able to provide multiple reasons for their selection. As a result, sum of numbers in columns may exceed the total number of respondents.

1 AMR-Antimicrobial resistance.

2 NS-National stakeholders.

3 HP-Health provider.

Participants also cited more pressing health issues and concern with increasing the number of injections given to children:

*“As long as we have the onslaught of life-threatening diseases, Shigella is not a priority.” - A national stakeholder in Burkina Faso*

*“I have a reservation regarding our national vaccine schedule, which is already too full. We risk scaring [mothers] and consequently losing many women.“ - A health provider in Burkina Faso*

### 3.5 Preferred attributes of a *Shigella* vaccine

**Fig. 4:**
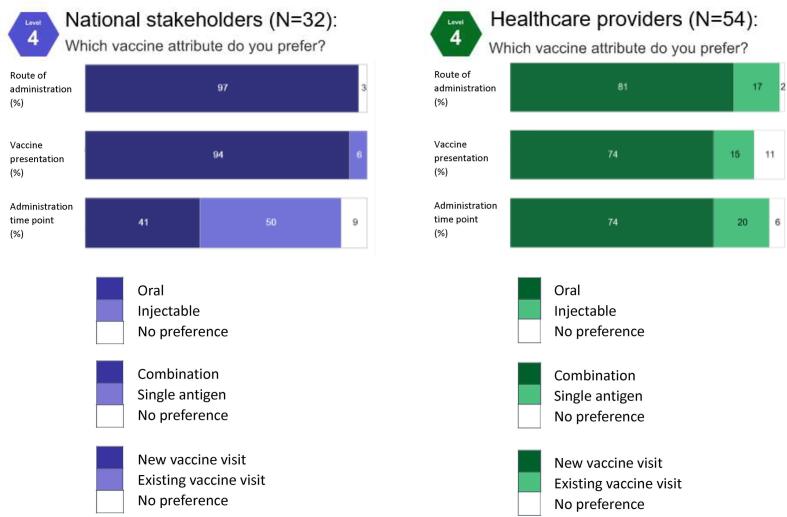
Preferred route of administration, vaccine presentation, and administration timing, by stakeholder group (%).

Participants were asked how specific vaccine delivery attributes would affect their willingness to consider introducing a *Shigella* vaccine. All national stakeholders citing a preferred route of administration selected an oral vaccine over an injectable (Fig. 4). Between 15% and 30% of healthcare providers across all countries preferred an injectable vaccine except in Nepal, where 100% preferred an oral vaccine (Supplemental material 5). National stakeholders expressed a strong preference for combination vaccines (Fig. 4); the only outlier was in Nepal, where 40% preferred a single-antigen presentation (Supplemental material 5). Healthcare providers were more mixed in their preferences for vaccine presentation (Fig. 4). When given the choice between administering the vaccine during an existing vaccination visit or creating a new visit, healthcare providers in all countries except Nepal were more likely to prefer a new visit, while national stakeholders were more balanced in their preference between new and existing timepoints (Fig. 4 and Supplemental material 5).

Across all attribute preferences, participant rationale focused on community and/or health worker acceptability, though framing varied depending on the attribute. When choosing between oral and injectable vaccines, participants felt oral vaccines would be more acceptable to the community because it would sidestep milder adverse reactions like injection pain:

*“As I said, injectable vaccines are painful for children, and they cause children to cry even when the mother holds them in their hands; It is hard for the mothers see their children suffering. The oral type is less painful therefore moms like it.” - A health provider in Burkina Faso*

When choosing a combination vaccine over a single-antigen presentation, many participants framed community acceptability around limiting the number of injections that a child receives in a single visit:

*“You will be giving multiple vaccines but one injection. The parents will be willing to bring their children because after all it is only a single shot.” - A health provider in Keny*a

A secondary theme for both oral and combination vaccines was increased acceptability to healthcare providers, described as easier to administer or minimizing workload:

*“It makes it easier for the population to adhere and for the health workers to do their job, and that is great.” – A national stakeholder in Burkina Faso*

Community acceptability was also the driver for choices around existing vaccination visits or new visits. Those preferring an existing visit cited greater convenience for parents and potentially improved vaccine uptake:

*“What we’ve learned from the introduction of malaria vaccine is that, when you add new visits […] communication around new visits can also be challenging and might affect optimal uptake.” - A national stakeholder in Ghana*

Responses from those preferring a new vaccine visit centered on a desire to minimize the number of injections given to a child in a single visit:

*“By the way, I do prefer the new contact because I hate when children take too many vaccines at the same time. Because it causes fevers and never-ending crying. I prefer it to be isolated at 6 months.” - A national stakeholder in Burkina Faso*

For all attribute comparisons (route of administration, presentation, and administration time point) participants were subsequently asked to consider availability of a vaccine with their non-preferred attribute. Preference for vaccine attributes was highly elastic, with approximately 10 percent of participants indicating that they would either be “much less willing to consider” or “would not consider” a vaccine with their non-preferred attribute. Most participants indicated that less-desirable vaccine attributes would not affect their interest if the potential impact of the vaccine justified the challenges of implementation.

**Fig. 5:**
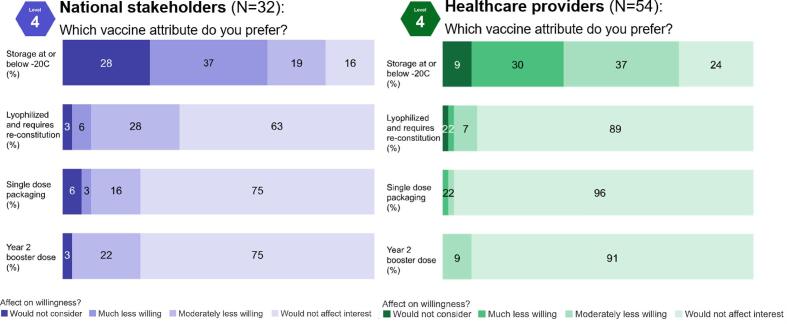
Vaccine delivery attributes on willingness to introduce vaccine, by stakeholder group (%).

Participants were asked to consider a −20 °C storage requirement, a lyophilized product, single dose packaging, and a requirement of a booster dose given in the second year of life (Fig. 5). Across both groups, the only attribute participants were consistently unwilling to consider was a vaccine requiring a −20 °C cold chain.

PAHO regional stakeholders unanimously preferred an oral and combination presentation for a *Shigella* vaccine compared to injection and single antigen presentations, citing themes of community acceptance, similar to country stakeholders. All three participants from PAHO cited the advantages of an oral vaccine in terms of perceived parent acceptability of fewer injections as well as the logistical ease of administration for healthcare workers:

*“If you have an oral vaccine, it is much easier for the child, but also in terms of the training that you do at local level, it is easier when you have an oral vaccine than when you have an injectable vaccine.” – A PAHO stakeholder*

The rationale for preferences of combination vaccines also centered around simplifying work for health workers:

*“If the biological possibility exists, I would always recommend having it in combination, because it is simpler, it is easier. You just tell people, ‘Look you are not only getting your child immunized against what used to be, but plus this.’” – A PAHO stakeholder*

Two of the three PAHO respondents preferred introducing a *Shigella* vaccine at a new immunization visit, citing community acceptability and safety concerns with an additional concomitant injection.

## 4 Discussion

Results from our study provide current insights into country and regional priorities for health and disease prevention for children under five years of age in LMICs and around *Shigella* vaccines, in particular, and support several of the findings from a multi-country study in Asia of policy-maker views on enteric vaccines conducted by DeRoeck *et al* in the early 2000s [30]. Across the five study countries, we found widespread participant recognition of childhood diarrhea, stunting, and AMR as “very serious” or “serious” health concerns. However, citing the relatively low disease burden estimates compared to other vaccine preventable diseases, most participants, especially national stakeholders, did not consider *Shigella* a very serious health concern and similar to findings by DeRoeck *et al*, many requested more country-specific data on both the burden of *Shigella* diarrhea and long-term health consequences [30]. This could, in part, be addressed by improving the dissemination of existing data, such as from pivotal diarrhea etiology studies such as GEMS or MAL-ED [9], [11], [31], as well as generating additional information by increasing the availability of *Shigella* diagnostics and expanding surveillance in additional LMICs.

While participants had an overall positive view of vaccines and indicated a willingness to consider new introductions, when provided global *Shigella* diarrhea and mortality estimates, just 15% of national stakeholders and 55% of healthcare providers cited a *Shigella* vaccine as a high priority. Even after given additional estimates of country-specific vaccine impact, roughly 40% fewer national stakeholders cited a *Shigella* vaccine as a high priority compared to healthcare providers, potentially reflecting greater uncertainty around the impact of a *Shigella* vaccine and wider awareness of the potential value of competing health interventions and/or national financial constraints.

We found important differences in priorities when data were disaggregated by country. Unlike perceptions of their peer groups in Burkina Faso, Ghana, Kenya, and Nepal, diarrhea was less of a concern in Vietnam, where approximately one-third of national stakeholders and healthcare providers perceived it as “not a very serious concern.” This lack of concern was also expressed about *Shigella,* which translated into a lower priority for a *Shigella* vaccine in Vietnam, particularly among national stakeholders. One potential factor behind this difference in response could be related to Vietnam’s lack of external immunization financing.

A country’s eligibility for Gavi financing for immunization depends on its gross national income (GNI); an eligible country’s GNI determines its Gavi “transition” phase, which corresponds to increasing co-financing requirements [32]. Burkina Faso and Nepal currently receive relatively more financial support, being in the lower GNI “initial self-financing” and “preparatory transition” Gavi phases, respectively, while Ghana and Kenya are in the mid-range “accelerated transition” phase, and the higher GNI of Vietnam deems it “fully self-financing.” We found that national stakeholders’ priority of a *Shigella* vaccine inversely correlated with their country’s Gavi transition phase: national stakeholders from Burkina Faso and Nepal were more likely to rate a *Shigella* vaccine a “medium” or “high” priority compared to those in Ghana and Kenya, and even more likely than their peers from Vietnam. This may reflect a different priority-setting calculus for introducing new vaccines in countries that are unable to rely on sustained external funding for their immunization programs. LMICs with limited resources are challenged by increasingly more complex and expensive immunization programs as new vaccines against priority pathogens are developed, forcing them to make difficult choices between competing priorities. Funders of *Shigella* vaccine research should take these considerations into account when assessing the relative merits of specific vaccine presentations. For example, the development of *Shigella*-containing combination vaccines that cover multiple pathogens, currently a secondary consideration, might actually be necessary to ensure vaccine adoption given countries’ increasingly crowded immunization schedules [24].

We found *Shigella* vaccine priority highest when linked to country-specific impacts on longer-term health issues, such as stunting and AMR. Similar concerns of increasing AMR related to *Shigella* were reported by DeRoeck *et a*l in Thailand, Bangladesh, Pakistan, and China [30]. While willing to consider introducing a *Shigella* vaccine, all groups, and particularly healthcare workers, expressed some reservations regardless of potential impact, again citing the relatively low burden of *Shigella* compared to other vaccine-preventable diseases and considered the diarrhea burden at least partially addressed through existing programs, including rotavirus vaccination and nutrition and water and sanitation programs [21], [22], [23]. Planned clinical efficacy trials of *Shigella* vaccine candidates might, in addition to providing evidence of the relative importance of *Shigella* on diarrhea, serve as vaccine probe studies if they also examined linear growth faltering, which has been feasibly assessed in multiple nutritional intervention studies [33], [34]. This would provide much needed evidence of the potential larger public health value of a *Shigella* vaccine—beyond acute diarrhea—which could result in more impactful messaging on *Shigella,* and in turn, may raise the priority of a *Shigella* vaccine.

Increased advocacy and communications about *Shigella* could also help build public priority and political will for a *Shigella* vaccine. The case for *Shigella* vaccination in general has not been well articulated among decision-makers, healthcare providers, and community members in the countries with the highest burden, regardless of their eligibility for Gavi support. A robust communication strategy to reach these groups, accounting for country-specific variations in perceptions (such as those shown here) could help improve awareness and understanding of the disease burden and drive demand for a *Shigella* vaccine.

Consideration of specific vaccine delivery attributes indicate that despite considerable elasticity, oral and combination vaccines were preferred over injectables and single-antigen presentations for a *Shigella* vaccine. We were surprised to find no indication of widespread concern over impaired effectiveness of oral presentations in low-income contexts as documented for rotavirus, cholera, and polio vaccines [35], [36], [37], [38], [39], [40]. Another unexpected finding was preferences around vaccination timing, which strayed from conventional wisdom of preferentially adding new vaccines to existing vaccination timepoints. While national stakeholders were evenly split, healthcare providers were more likely to prefer a new vaccine visit. While acknowledging that awareness-raising and uptake of new vaccines are challenging when novel immunization timepoints are introduced, we found widespread reluctance to adding additional injections onto current immunization schedules considered already too full. As new vaccines continue to be added to the immunization schedule, there is need for considering how to alleviate both overburdened providers and community concerns about multiple injections given at a single visit. These could be addressed through task shifting, similar to recommendations in human immunodeficiency (HIV) care [41], the addition of new immunization timepoints, or encouraging strong health provider recommendations and sharing information about the severity of target diseases, which have been associated with overcoming parental concerns with concomitant injections [42]. Of note, these attributes were considered in the context of a *Shigella* vaccine and should be generalized to the introduction of other antigens with caution; it is possible that there may be fewer attribute preferences for new vaccines for higher-burden diseases, such as malaria.

Strengths of our study approach include involving both national stakeholders and clinical practitioners and including five countries with a range of income levels and diarrheal disease and stunting burdens from both Africa and Asia. We also included stakeholders from the PAHO region. There are several limitations to our study. Without the availability of country-specific data, we used modeled estimates of *Shigella* diarrhea morbidity and mortality [8] and a hypothetical 60% vaccine impact across all outcomes; this may have biased our results towards higher prioritization if the numbers we provided over-estimated true disease burden and a *Shigella* vaccine’s effectiveness. We also estimated a relatively low vaccine cost of USD$1/dose based on estimates of typhoid conjugate vaccine. However, this, along with the assumption of support from Gavi for eligible countries, is uncertain and may have biased our results towards more favorable views of the vaccine. With an availability timeline as early as 2025, we do not know the full landscape of vaccine competitors, and several of our study countries may have transitioned out of Gavi support and be fully self-supporting of vaccine introduction costs by this time, which would likely impact vaccine introduction decisions. Also, participants did not represent all geographic areas of their respective countries and thus preferences and perspectives may not be representative of the country as a whole nor of other LMICs. In addition to limited participation from the PAHO region, we were unable to collect data in PAHO countries due to limitations on travel during the SARS CoV-2 pandemic. Similarly, restrictions on social interactions prevented us from interviewing community members, although their views on vaccine acceptability are critical for the success of vaccine uptake.

Beyond *Shigella*, historical efforts to gain insight into preferences around vaccine attributes have seldom involved systematic surveys of those most directly involved at the country level. Recent precedents by Price and Mooney *et al* considered novel rotavirus vaccines which found similar preferences for oral and combination presentations [43], [44]. Our study is timely, since *Shigella* vaccine candidates are still in development, providing manufacturers an opportunity to incorporate our findings into clinical trials plans, such as evaluating vaccine impact beyond diarrhea by integrating a stunting endpoint into Phase 3 clinical studies. It also provides perspectives from country and regional decision-makers and implementers from LMICs to inform key vaccine delivery attributes of candidates in earlier stages of the vaccine development process. While our study is valuable by itself, it is even more so when considered in conjunction with other analyses on *Shigella* vaccine cost-effectiveness, challenges and obstacles to *Shigella* combination vaccine development, and the economic loss of stunting [21], [22], [23], [24], [45]. If acted upon, our findings could result in *Shigella* vaccines that are more suitable—e.g., in the form of an oral formulation or a combination that includes a licensed vaccine given at the same age—and attractive to stakeholders in countries that need them the most.

## 5 Conclusions

Given numerous competing disease prevention priorities in LMICs, input from key stakeholders in countries most at risk is required to accurately understand and estimate country demand for any new vaccine candidate, but particularly for those targeted against pathogens that do not rank among the top causes of morbidity and mortality at the global level, such as *Shigella*. Now, while *Shigella* vaccines are still in clinical development, is a critical time to understand preferences from decision makers who, along with national governments, may have the final say in whether a vaccine is introduced, and from healthcare providers who will be essential in implementing *Shigella* vaccine roll-out. The results provided here highlight several key elements that we believe vaccine developers, funders, and advocates should take into account: (i) there is wide country-specific variability in the perceptions of *Shigella* disease and vaccines, which will require tailored approaches in education and communication; (ii) a shared perception that the prospects of ameliorating growth faltering and/or AMR might drive adoption of *Shigella* vaccines, and thus there is need to demonstrate, in clinical studies, that vaccines could indeed have those effects; (iii) a major barrier to adoption of a *Shigella* vaccine may lie in the necessity of additional injections, pointing to the need for investments in a combination formulation that simultaneously offers protection against other priority pathogens. Our study results add to the limited but growing knowledge base of how stakeholders in LMICs prioritize *Shigella* and a potential vaccine and can inform the design of more attractive vaccine formulations and clinical trial endpoints that address stakeholder priorities. Ultimately, if *Shigella* vaccine development is successful, our findings will aid in the development of appropriate communication and advocacy strategies for adoption and implementation of these important vaccines.

**Declarations**

Ethics approval and consent to participate

The study received exempt status from WIRB-Copernicus Group Institutional Review Board (IRB) (Olympia, WA, USA) and country approvals from Burkina Faso’s Comité d'Ethique pour la Recherche en Santé, the Ghana Health Service Ethics Review Committee, the Kenyatta National Hospital-University of Nairobi Research Ethics Committee, the Nepal Health Research Council and B.P. Koirala Institute of Health Sciences in Dharan, Nepal, and Vietnam’s National Institute of Hygiene & Epidemiology IRB. Informed consent was obtained from each participant prior to interviews.

Author’s contributions

Study concept and design: JAF, NG, SK1, WPH. Data collection: LK, PD, KA, CO1, SW, SU, LTPM, NTMH, LPD, WPH. Country leads: LK, JTB, PD, CO1, CO2, SK2, TT, WPH. Data analysis: JAF, NG, SK1. Drafting of manuscript: JAF, NG, SK1. Review and feedback of manuscript: All authors read and provided feedback on the manuscript and approved the final manuscript.

## Declaration of Competing Interest

The authors declare the following financial interests/personal relationships which may be considered as potential competing interests: Jessica A Fleming, Nikki Gurley, Sophia Knudson, Lassane Kabore, John Tanko Bawa, Patience Dapaah, Sandeep Kumar, Thang Tran, Chris Odero, Christopher Obong’o, Kofi Aburam, Stella Wanjiru, and William P Hausdorff report financial support was provided by Wellcome Trust and financial support and article publishing charges were provided by Bill & Melinda Gates Foundation..

## Data Availability

The datasets used and/or analyzed during the current study are available from the corresponding author on reasonable request.

## References

[1] IHME GBD 2016 Diarrhoeal Disease Collaborators (2018). Estimates of the global, regional, and national morbidity, mortality, and aetiologies of diarrhoea in 195 countries: a systematic analysis for the Global Burden of Disease Study 2016 - the Lancet Infectious Diseases. Lancet Infect Dis.

[2] Kotloff K. (1999). Global burden of Shigella infections: implications for vaccine development and implementation of control strategies. Bull World Health Organ.

[3] Research priorities for diarrhoeal disease vaccines: memorandum from a WHO meeting. Bull World Health Organ 1991;69(6):667–76. PMC2393325 1664785

[4] Huilan S., Zhen L.G., Mathan M.M., Mathew M.M., Olarte J., Espejo R. (1991). Etiology of acute diarrhoea among children in developing countries: a multicentre study in five countries. Bull World Health Organ.

[5] Butkeviciute E., Prudden H.J., Jit M., Smith P.G., Kang G., Riddle M.S. (2021). Global diarrhoea-associated mortality estimates and models in children: Recommendations for dataset and study selection. Vaccine.

[6] Kotloff K.L., Riddle M.S., Platts-Mills J.A., Pavlinac P., Zaidi A.K.M. (2018). Shigellosis. Lancet.

[7] Kotloff K.L., Platts-Mills J.A., Nasrin D., Roose A., Blackwelder W.C., Levine M.M. (2017). Global burden of diarrheal diseases among children in developing countries: Incidence, etiology, and insights from new molecular diagnostic techniques. Vaccine.

[8] Anderson J.D., Bagamian K.H., Muhib F., Amaya M.P., Laytner L.A., Wierzba T. (2019). Burden of enterotoxigenic Escherichia coli and shigella non-fatal diarrhoeal infections in 79 low-income and lower middle-income countries: a modelling analysis - the Lancet Global Health. Lancet Glob Health.

[9] Kotloff KL, Nataro JP. Burden and aetiology of diarrhoeal disease in infants and young children in developing countries (the Global Enteric Multicenter Study, GEMS): a prospective, case-control study - The Lancet. [cited 2022 Nov 21]. <https://www.thelancet.com/journals/lancet/article/PIIS0140-6736(13)60844-2/fulltext>. 10.1016/S0140-6736(13)60844-2 23680352

[10] Liu J., Platts-Mills J.A., Juma J., Kabir F., Nkeze J., Okoi C. (2016). Use of quantitative molecular diagnostic methods to identify causes of diarrhoea in children: a reanalysis of the GEMS case-control study. Lancet.

[11] Platts-Mills J.A., Babji S., Bodhidatta L., Gratz J., Haque R., Havt A. (2015). Pathogen-specific burdens of community diarrhoea in developing countries: a multisite birth cohort study (MAL-ED) - the Lancet Global Health. Lancet Glob Health.

[12] Tickell K.D., Brander R.L., Atlas H.E., Pernica J.M., Walson J.L., Pavlinac P.B. (2017). Identification and management of Shigella infection in children with diarrhoea: a systematic review and meta-analysis - the Lancet Global Health. Lancet Glob Health.

[13] Hosangadi D., Smith P.G., Giersing B.K. (2019). Considerations for using ETEC and Shigella disease burden estimates to guide vaccine development strategy. Vaccine.

[14] Guerrant R.L., DeBoer M.D., Moore S.R., Scharf R.J., Lima A.A.M. (2012). The impoverished gut—a triple burden of diarrhoea, stunting and chronic disease. Nat Rev Gastroenterol Hepatol.

[15] Li S., Saha D., Omore R., Stine O.C., Panchalingam S., Kotloff K. (2015). Association between shigella infection and diarrhea varies based on location and age of children. Am J Trop Med Hyg.

[16] Anderson J.D., Bagamian K.H., Muhib F., Baral R., Laytner L.A., Amaya M. (2019). Potential impact and cost-effectiveness of future ETEC and Shigella vaccines in 79 low- and lower middle-income countries. Vaccine: X.

[17] Puzari M., Sharma M., Chetia P. (2018). Emergence of antibiotic resistant Shigella species: a matter of concern. J Infect Public Health.

[18] Ranjbar R., Farahani A. (2019). Shigella: antibiotic-resistance mechanisms and new horizons for treatment. Infect Drug Resist.

[19] Niyogi S.K. (2007). Increasing antimicrobial resistance—an emerging problem in the treatment of shigellosis. Clin Microbiol Infect.

[20] WHO. WHO preferred product characteristics for vaccines against Shigella [Internet]. Geneva; 2021 [cited 2022 Dec 19]. Report No.: ISBN 978-92-4-003674-1. <https://www.who.int/publications-detail-redirect/9789240036741>.

[21] Bagamian K.H., Puett C., Anderson J.D., Muhib F., Pecenka C., Behrman J. (2022). Could a Shigella vaccine impact long-term health outcomes?: Summary report of an expert meeting to inform a Shigella vaccine public health value proposition, March 24 and 29, 2021. Vaccine: X.

[22] Anderson J.D., Bagamian K.H., Pecenka C.J., Muhib F., Puett C.A., Hausdorff W.P. (2023). Potential impact and cost-effectiveness of Shigella vaccination in 102 low-income and middle-income countries in children aged 5 years or younger: a modelling study. Lancet Global Health.

[23] Puett C., Anderson J.D., Bagamian K.H., Muhib F., Scheele S., Hausdorff W.P. (2023). Projecting the long-term economic benefits of reducing Shigella-attributable linear growth faltering with a potential vaccine: a modelling study. Lancet Global Health.

[24] Riddle M.S., Louis Bourgeois A., Clifford A., Jeon S., Giersing B.K., Jit M. (2023). Challenges and opportunities in developing a Shigella-containing combination vaccine for children in low- and middle-income countries: Report of an expert convening. Vaccine.

[25] Khalil I.A., Troeger C., Blacker B.F., Rao P.C., Brown A., Atherly D.E. (2018). Morbidity and mortality due to shigella and enterotoxigenic Escherichia coli diarrhoea: the Global Burden of Disease Study 1990–2016. Lancet Infect Dis.

[26] Anderson J. Personal communication; 2021.

[27] Bharat Biotech, PATH, Bill & Melinda Gates Foundation, The Clinton Health Access Initiative (CHAI), Oxford University. Typbar TCV® from Bharat Biotech, World’s First Typhoid Conjugate Vaccine Prequalified by WHO [Internet]. Bharat Biotech; 2018 [cited 2022 Dec 19]. <https://bharatbiotech.com/images/press/World's-First-Typhoid-Conjugate-Vaccine-Prequalified-by-WHO-Jan-2018.pdf>.

[28] Harris P.A., Taylor R., Thielke R., Payne J., Gonzalez N., Conde J.G. (2009). Research electronic data capture (REDCap)–a metadata-driven methodology and workflow process for providing translational research informatics support. J Biomed Inform.

[29] Best Qualitative Data Analysis Software for Researchers | NVivo [Internet]. [cited 2022 Nov 21]. <https://www.qsrinternational.com/nvivo-qualitative-data-analysis-software/home>.

[30] DeRoeck D., Clemens J.D., Nyamete A., Mahoney R.T. (2005). Policymakers’ views regarding the introduction of new-generation vaccines against typhoid fever, shigellosis and cholera in Asia. Vaccine.

[31] Kotloff K.L., Nasrin D., Blackwelder W.C., Wu Y., Farag T., Panchalingham S. (2019). The incidence, aetiology, and adverse clinical consequences of less severe diarrhoeal episodes among infants and children residing in low-income and middle-income countries: a 12-month case-control study as a follow-on to the Global Enteric Multicenter Study (GEMS). Lancet Glob Health.

[32] Gavi, The Vaccine Alliance. Gavi Application Process Guidelines [Internet]. 2023 Apr. <https://www.gavi.org/sites/default/files/support/ApplicationProcess_Guidelines.pdf>.

[33] Pavlinac P.B., Rogawski McQuade E.T., Platts-Mills J.A., Kotloff K.L., Deal C., Giersing B.K. (2022). Pivotal shigella vaccine efficacy trials—study design considerations from a shigella vaccine trial design working group. Vaccines.

[34] Park J.J.H., Harari O., Siden E., Dron L., Zannat N.-E., Singer J. (2019). Interventions to improve linear growth during complementary feeding period for children aged 6–24 months living in low- and middle-income countries: a systematic review and network meta-analysis. Gates Open Res.

[35] Madhi S.A., Cunliffe N.A., Steele D., Witte D., Kirsten M., Louw C. (2010). Effect of human rotavirus vaccine on severe diarrhea in African infants | NEJM. N Engl J Med.

[36] Suharyono, Simanjuntak C., Totosudirjo H., Witham N., Punjabi N., Burr D. (1992). Safety and immunogenicity of single-dose live oral cholera vaccine CVD 103-HgR in 5-9-year-old Indonesian children. Lancet.

[37] Gotuzzo E., Butron B., Seas C., Penny M., Ruiz R., Losonsky G. (1993). Safety, immunogenicity, and excretion pattern of single-dose live oral cholera vaccine CVD 103-HgR in Peruvian adults of high and low socioeconomic levels | Infection and Immunity. Infect Immun.

[38] Richie E., Punjabi N.H., Sidharta Y., Peetosutan K., Sukandar M., Wasserman S.S. (2000). Efficacy trial of single-dose live oral cholera vaccine CVD 103-HgR in North Jakarta, Indonesia, a cholera-endemic area. Vaccine.

[39] Patriarca P.A., Wright P.F., John T.J. (1991). Factors affecting the immunogenicity of oral poliovirus vaccine in developing countries: review. Rev Infect Dis.

[40] Parker E.PK., Ramani S., Lopman B.A., Church J.A., Iturriza-Gómara M., Prendergast A.J. (2018). Causes of impaired oral vaccine efficacy in developing countries. Future Microbiol.

[41] World Health Organization, PEPFAR, UNAIDS. Task shifting : rational redistribution of tasks among health workforce teams : global recommendations and guidelines. 2007;88.

[42] Wallace A.S., Mantel C., Mayers G., Mansoor O., Gindler J.S., Hyde T.B. (2014). Experiences with provider and parental attitudes and practices regarding the administration of multiple injections during infant vaccination visits: lessons for vaccine introduction. Vaccine.

[43] Mooney J., Price J., Bain C., Bawa J.T., Gurley N., Kumar A. (2022). Healthcare provider perspectives on delivering next generation rotavirus vaccines in five low-to-middle-income countries. PLoS One.

[44] Price J., Mooney J., Bain C., Bawa J.T., Gurley N., Kumar A. (2022). National stakeholder preferences for next-generation rotavirus vaccines: Results from a six-country study - ScienceDirect. Vaccine.

[45] Hausdorff W.P., Scheele S., Giersing B.K. (2022). What Drives the Value of a Shigella Vaccine?. Vaccines.

