# Supplementary material for: Exploring Shigella vaccine priorities and preferences: Results from a mixed-methods study in low- and middle-income settings
Source: Vaccine X. 2023 Aug 9;15:100368. doi: 10.1016/j.jvacx.2023.100368 (PMC10457597; doi:10.1016/j.jvacx.2023.100368)
Supplement: Supplementary data 1 [file mmc1.docx]

**Supplemental material 1: Study questionnaire**

*Exploring Shigella vaccine priorities and preferences: Results from a mixed-methods study in low- and middle-income settings;*

Fleming, Gurley, Knudson *et al.*

| # **Variable/ Field Name Field Label**  *Field Note* | | | | **Field Attributes (Field Type, Validation, Choices, Calculations, etc.)** | | | | |
| --- | --- | --- | --- | --- | --- | --- | --- | --- |
| Instrument: **INTERVIEW GUIDE - ENGLISH** (interview_guide_english) | | | | | | | | |
| 1 [unique_id] Unique ID | | | | text | | | | |
|  | 2 | [ recording_confirmation J | X.1 Have you turned on the audio recording? | required | | | | |
|  | 3 | [recording_start_script J | X.2 Speak into the recording:  "Starting the interview with respondent [unique_id]." | descriptive | | | | |
|  | 4 | [ interview_date J | X.3 Date interview conducted | text (date_dmy), Required Field Annotation: @TODAY | | | | |
|  | 5 | [ interview_location J | X.4 Location of interview | radio, Required | | | | |
|  |  |  |  | 1 | Burkina Faso |  | | |
|  |  |  |  | 2 | Ghana |  |  |  |
|  |  |  |  | 3 | Kenya |  |  |  |
|  |  |  |  | 4 | Nepal |  |  |  |
|  |  |  |  | 5  - | Vietnam |  |  |  |
| I 6 | | [ city_town_district] | X4.1 Name of City or Town and District | text, Required | | | | |
|  | 7 | [ interview_level J | X.5 Level of interview | radio, Required | | | | |
|  |  |  |  | 1 | National level | | |  |
|  |  |  |  | 2 | Health facility level | | |  |
|  | 8 |  |  |  | | | | |
|  |  | [ hcf_level J  Show the field ONLY if: [interview_level] = '2' | X.5.3 What level facility are you at? | radio, Required | | | | |
|  |  |  |  | 1 | Primary level | |  | |
|  |  |  |  | 2 | Secondary level | |  |  |
|  |  |  |  | 3 | Tertiary level | |  |  |
|  | 9 |  |  |  | | | | |
|  |  | [ hcf_publicvprivate J  Show the field ONLY if: [interview_level] = '2' | X.5.4 ls the health facility public or private? | radio, Required | | |  | |
|  |  |  |  | 1 | Public facility |  |  |  |
|  |  |  |  | 2 | Private facility | |  |  |
|  | 10 |  |  |  | | |  |  |
|  |  | [hcf_name]  Show the field ONLY if: [interview_level] = '2' | X.5.5 Name of health facility | text, Required, Identifier | | | | |
|  | 11 | [a_org] | Section Header: *Section A: Stakeholder profile*  1.1 What organization do you work for? *Do not need to type the answer - audio recording will capture. | text, Identifier | | | | |
| 12  I | | [a_title J | 1.2 What is your title/role? *Do not need to type the answer  - audio recording will capture. | text, Identifier | | | | |
|  | 13 | [ a_years_experience J | 1.3 How many years have you been in your current role?  *Do not need to type the answer - audio recording will capture. | text, Identifier | | | | |
|  | 14 | [a_nitag_icc]  Show the field ONLY if: [interview_level] = '1' or [hcf_l evel] = '3' | 1.4 Are you a member of the National Immunization Technical Advisory Group (NITAG) or Inter-agency Coordinating Committee (ICC), or do you regularly attend these meetings? | yesno, Required  0 | | | | |
|  | 15 | [ b_u5_health_concerns] | Section Header: *Section B: Health priorities and perceptions of diarrheal*  ***disease***  2.1 We are going to start by discussing the child health in [interview_location]. From your perspective, can you tell me about the 2-3 most important health or development concerns in children under-five years of age in [interview_location]? *Do not need to type the answer - audio recording will capture. | text | | | | |

I

I

|  | 16 | [ b_uS_health_concern] | 2.2 How important is diarrhea as a health concern for children under-five years of age in your setting? | radio, Required | | | |
| --- | --- | --- | --- | --- | --- | --- | --- |
|  |  |  |  | 1 | A very serious health concern | | |
|  |  |  |  | 2 | A serious problem, but not among the top health concern | | |
|  |  |  |  | 3  - | Not a very serious problem compared to other health concern | | |
|  | 17 | [ b_u5_health_concern_why] | 2.2.1 Why? *Do not need to type the answer - audio recording will capture.  *Probe: acute symptoms, sequelae, clinical, economic, or social impacts.* | text | | | |
|  | 18 | [ b_interv_priority] | 2.3 What interventions do you consider your first and second priorities for diarrhea prevention? *Do not need to type the answer - audio recording will capture.  ***Probe: vaccines (rotavirus, cholera), water/sanitation, nutrition, hand***  *washing.* | text | | | |
|  | 19 | [ b_importance_growth] | 2.4 How important is growth stunting as a health concern for children under-five years of age in [interview_location]? | radio, Required | | | |
|  |  |  |  | 1 | A very serious health concern | | |
|  |  |  |  | 2 | A serious problem, but not among the top health concern | | |
|  |  |  |  | 3 | Not a very serious problem compared to other health concern | | |
|  | 20 |  |  |  | | | |
|  |  | [b_importance_growth_why] | 2.4.1 Why? *Do not need to type the answer - audio recording will capture.  *Probe: acute symptoms, sequelae, clinical or social impacts.* | text | | | |
|  | 21 | [ b_heard_of_shigella] | 2.5 Have you heard of Shigella? | radio, Required  0 | | | |
|  | 22 | [ shigella_awareness]  Show the field ONLY if: [b_heard_of_shigella] = '1' | 2.6 Tell me what you have heard about Shigella. *Do not need to type the answer - audio recording will capture. *Probe: burden/high-risk populations, source of transmission, diagnosis, symptoms, sequelae, and treatment* | text | | | |
|  | 23 | [ b_shigella_importance]  Show the field ONLY if: [b_heard_of_shigella] = '1' | 2.7 How important is Shigella as a health concern for children under-five years of age in your setting? | radio, Required | | | |
|  |  |  |  | 1 | | A very serious health concern | |
|  |  |  |  | 2 | | A serious problem, but not among the top health concerns | |
|  |  |  |  | 3 | | Not a very serious problem compared to other health concerns | |
|  |  |  |  | 99 | | Don't know | |
|  | 24 |  |  |  | | | |
|  |  | [ b_shigella_aspects]  Show the field ONLY if: [b_heard_of_shigella] = '1' | 2.8 What aspects of Shigella are the most concerning to you? *Do not need to type the answer - audio recording will capture.  *Probe: importance of diarrhea (acute symptoms}, stunting (sequelae}, and*  *clinical or social impacts.* | text | | | |
|  | 25 | [ b_shigella_priority]  Show the field ONLY if: [b_heard_of_shigella] = '1' | 2.9 Consider that a vaccine against Shigella is available. What is the priority you would place on introducing a vaccine against Shigella for young children in [interview_location]? | radio, Required | | | |
|  |  |  |  | 1 | High priority | |  |
|  |  |  |  | 2 | Medium priority | |  |
|  |  |  |  | 3 | Low priority | |  |
|  |  |  |  | 4 | Not a priority | |  |
|  | 26 |  |  |  | | | |
|  |  | [ c_amr_importance] | Section Header: *Section C: Anti-Microbial Resistance One of the* ***interventions used to treat diarrhea is antibiotics. We are aware that "anti-*** *microbial resistance" is a growing concern for many pathogens globally.*  3.1 How important is anti-microbial resistance as a health concern in your setting? | radio, Required | | | |
|  |  |  |  | 3 | A very serious health issue | | |
|  |  |  |  | 2 | A serious problem, but not among the top health issues | | |
|  |  |  |  | 1 | Not a very serious problem compared to other health issues | | |
|  |  |  |  | 0 | Don't know | | |
| 27  I | | [ c_amr_importance_why] | 3.1.1 Why? *Do not need to type the answer - audio recording will capture. | text | | | |

I

| 1 | Yes |
| --- | --- |
| 0 | No |
| 99  - | Don't know |

| 1 | Yes |
| --- | --- |
| 0 | No |
| 99 | Don't know |

| 28 | [ c_amr_treatment_costs]  Show the field ONLY if: [interview_level] = '2' | 3.2 Have treatment practices in health facilities changed due to increases in anti-microbial resistance over the last five years in [interview_location]? | radio, Required |
| --- | --- | --- | --- |
| 29 | [ c_amr_treatment_costs_yes]  Show the field ONLY if: [c_amr_treatment_costs] = '1' | 3.2.1 How? *Do not need to type the answer - audio recording will capture. | text |
| 30 | [ c_amr_ns_priority]  Show the field ONLY if: [interview_level] = '1' | 3.3 Is information on anti-microbial resistance of a target pathogen taken into consideration when prioritizing the introduction of a new vaccine against that pathogen in [interview_location]? | radio, Required |
| 31 | [ c_amr_ns_priority_yes]  Show the field ONLY if: [c_amr_ns_priority] = '1' and [i nterview_level] = '1' | 3.3.1 How? *Do not need to type the answer - audio recording will capture. | text |
| 32 | [ c_amr_ns_priority_no_dn]  Show the field ONLY if: [c_amr_ns_priority] = 'O' or [c_ amr_ns_priority] = '99' | 3.3.1 Is it criteria that needs to be considered? *Do not need to type the answer - audio recording will capture. | text |
| 33 | [visual_aid_1 ] | Section Header: *Section D: Shigello Vaccine Impact For investigators only: Please refer to visual aid# 1 for preamble section. Shigella is a bacterial pathogen that can cause severe diarrhea, including bloody diarrhea or 'dysentery; and it is a significant cause of morbidity and mortality in children. The global burden of Shigella diarrhea in children less than five years of age has been estimated at* 75 *million cases and 64,000 deaths annually, making it potentially the second leading cause of diarrhea mortality.(1) Vaccines against Shigella are currently in development and may be available in 2025-2030. A hypothetical Shigella vaccine is expected to be injectable and require one or two doses for the primary series, given mid- to late in the first year of life. Consider the vaccine costs around US$1ldose and would initially be supported by Gavi. Assume that your country will be considering multiple vaccines for introduction in 2025- 2030, when a Shigella vaccine is likely to be available. (1) Khalil, The Lancet, 2018.* | descriptive |
| 34 | [d_immun_sched] | 4.1 Do you think [interview_location] has room in its routine immunization schedule to consider adding a vaccine against Shigella? | quired |
| 35 | [ d_immun_sched_yes] | 4.1.1 What reservations, if any, do you have about adding a new vaccine to [interview_location]'s rountine immunization schedule? *Do not need to type the answer - audio recording will capture. | text |
| 36 | [visual_aid_2_bk]  Show the field ONLY if: [interview_location] = '1' | For investigators only: Please refer to visual aid #2 for question 4.2. | descriptive |
| 37 | [visual_aid_2_ghana]  Show the field ONLY if: [interview_location] = '2' | For investigators only: Please refer to visual aid #2 for question 4.2. | descriptive |
| 38 | [visual_aid_2_kenya]  Show the field ONLY if: [interview_location] = '3' | For investigators only: Please refer to visual aid #2 for question 4.2. | descriptive |
| 39 | [visual_aid_2_nepal]  Show the field ONLY if: [interview_location] = '4' | For investigators only: Please refer to visual aid #2 for question 4.2. | descriptive |
| 40 | [visual_aid_2_vietnam]  Show the field ONLY if: [interview_location] = 'S' | For investigators only: Please refer to visual aid #2 for question 4.2. | descriptive |

1. [ d_bk_vaccine_priority]

Show the field ONLY if: [interview_location] = 1

1. [ d_ghana_vaccine_priority J

Show the field ONLY if: [interview_location] = '2'

1. [ d_kenya_vaccine_priorityJ

Show the field ONLY if: [interview_location] = '3'

1. [ d_nepal_vaccine_priority J

Show the field ONLY if: [interview_location] = '4'

1. [ d_vietnam_vaccine_priority J

Show the field ONLY if: [interview_location] = '5'

1. [ d_vaccine_priority_why_bk J

Show the field ONLY if: [interview_location] = 1

1. [ d_vaccine_priority_why_ghJ

Show the field ONLY if: [interview_location] = '2'

1. [ d_vaccine_priority_why_ke J

Show the field ONLY if: [interview_location] = '3'

4.2 In [interview_location], it is estimated that Shigella is responsible for 95,400 cases of moderate to severe diarrhea and 530 deaths in children under five each year. Estimates are that a Shigella vaccine would be 60% effective and could prevent 52,075 moderate to severe diarrhea cases and 289 deaths due to Shigella diarrhea annually in [interview_location]. Given this burden and vaccine impact and knowing that this will not be the only vaccine your country considers in 2025-2030, what is the priority you would place on introducing a vaccine against Shigella in [interview_location]?

4.2 In [interview_location], it is estimated that Shigella is responsible for 115,500 cases of moderate to severe diarrhea and 220 deaths in children under five each year. Estimates are that a Shigella vaccine would be 60% effective and could prevent 67,231 moderate to severe diarrhea cases and 124 deaths due to Shigella diarrhea annually in [interview_location]. Given this burden and vaccine impact and knowing that this will not be the only vaccine your country considers in 2025-2030, what is the priority you would place on introducing a vaccine against Shigella in [interview_location]?

4.2 In [interview_location], it is estimated that Shigella is responsible for 209,000 cases of moderate to severe diarrhea and 590 deaths in children under five each year. Estimates are that a Shigella vaccine would be 60% effective and could prevent 115,342 moderate to severe diarrhea cases and 315 deaths due to Shigella diarrhea annually in [interview_location]. Given this burden and vaccine impact and knowing that this will not be the only vaccine your country considers in 2025-2030, what is the priority you would place on introducing a vaccine against Shigella in [interview_location]?

4.2 In [interview_location], it is estimated that Shigella is responsible for 48,400 cases of moderate to severe diarrhea and 85 deaths in children under five each year. Estimates are that a Shigella vaccine would be 60% effective and could prevent 27,008 moderate to severe diarrhea cases and 44 deaths due to Shigella diarrhea annually in [interview_location]. Given this burden and vaccine impact and knowing that this will not be the only vaccine your country considers in 2025-2030, what is the priority you would place on introducing a vaccine against Shigella in [interview_location]?

- 1. In [interview_location], it is estimated that Shigella is responsible for 6,800 cases of moderate to severe diarrhea and 5 deaths in children under five each year. Estimates are that a Shigella vaccine would be 60% effective and could prevent 3,925 moderate to severe diarrhea cases and 3 deaths due to Shigella diarrhea annually in [interview_location]. Given this burden and vaccine impact and knowing that this will not be the only vaccine your country considers in 2025-2030, what is the priority you would place on introducing a vaccine against Shigella in [interview_location]?
     1. Why did you select '[d_bk_vaccine_priority]'?*Do not need to type the answer - audio recording will capture.
     2. Why did you select '[d_ghana_vaccine_priority]'? *Do not need to type the answer - audio recording will capture.
     3. Why did you select '[d_kenya_vaccine_priority]'?*Do not need to type the answer - audio recording will capture.

radio, Required

| 1 | High priority |
| --- | --- |
| 2 | Medium priority |
| 3 | Low priority |
| 4  - | Not a priority |

radio, Required

| 1 | High priority |
| --- | --- |
| 2 | Medium priority |
| 3 | Low priority |
| 4 | Not a priority |

radio, Required

| 1 | High priority |
| --- | --- |
| 2 | Medium priority |
| 3 | Low priority |
| 4 | Not a priority |

radio

| 1 | High priority |
| --- | --- |
| 2 | Medium priority |
| 3 | Low priority |
| 4 | Not a priority |

radio, Required

| 1 | High priority |
| --- | --- |
| 2 | Medium priority |
| 3 | Low priority |
| 4 | Not a priority |

text

text

text

I

|  | 49 | [ d_vaccine_priority_why_neJ  Show the field ONLY if: [interview_location] = '4' | 4.2.1 Why did you select '[d_nepal_vaccine_priority]'?*Do not need to type the answer - audio recording will capture. | text | | |
| --- | --- | --- | --- | --- | --- | --- |
|  | 50 | [ d_vaccine_priority_why_viet J  Show the field ONLY if: [interview_location] = '5' | 4.2.1 Why did you select '[d_vietnam_vaccine_priority]'?*Do not need to type the answer - audio recording will capture. | text | | |
|  | 51 | [ shigella_priority_slow_amr J | 4.3 If it was available, what is the priority you would place on a Shigella vaccine for young children that had the ability to slow the pace or prevent antibiotic resistance for Shigella in [interview_location]? | radio, Required | | |
|  |  |  |  | 1 | High priority |  |
|  |  |  |  | 2 | Medium priority |  |
|  |  |  |  | 3 | Low priority |  |
|  |  |  |  | 4  - | Not a priority |  |
| 52  I | | [ shigella_prioirity_amr_why J | 4.3.1 Why did you select '[shigella_priority_slow_amr]'? *Do not need to type the answer - audio recording will capture. | text | | |
|  | 53 | [visual_aid_3_bk]  Show the field ONLY if: [interview_location] = '1' | For investigator only: Please use visual aids #3 and #4 for question 4.4 preamble. | descriptive | | |
|  | 54 | [visual_aid_3_ghana J  Show the field ONLY if: [interview_location] = '2' | For investigator only: Please use visual aids #3 and #4 for question 4.4 preamble. | descriptive | | |
|  | 55 | [visual_aid_3_kenya J  Show the field ONLY if: [interview_location] = '3' | For investigator only: Please use visual aids #3 and #4 for question 4.4 preamble. | descriptive | | |
|  | 56 | [visual_aid_3_nepal J  Show the field ONLY if: [interview_location] = '4' | For investigator only: Please use visual aids #3 and #4 for question 4.4 preamble. | descriptive | | |
|  | 57 | [visual_aid_3_vietnam J  Show the field ONLY if: [interview_location] = '5' | For investigator only: Please use visual aids #3 and #4 for question 4.4 preamble. | descriptive | | |
| I 58 | | [visual_aid_4J |  | descriptive | | |
|  | 59 | [ d_description_3] | There is growing evidence that Shigella has some unique negative health and life consequences on individuals beyond causing an acute diarrhea episode. Shigella diarrhea early in childhood can impede the absorption of nutrients in the gut, leading to malnutrition. Shigella has been linked to growth stunting in children. It is also a known risk factor for other deaths due to infectious diseases in children and stunted children have been shown to have a higher risk of chronic diseases as adults. In addition, stunting has been associated with impaired physical and cognitive development and linked to lower educational attainment and ultimately lower earning power as adults. Consider that a vaccine that prevents Shigella could also help prevent some of these other negative health and life consequences. In this part of the interview, we want to understand how important the potential additional health and life benefits of a Shigella vaccine, beyond preventing acute diarrhea and associated deaths, is to you when considering introducing a vaccine against Shigella compared to other vaccines that [interview_location] may consider in 2025-2030, when a Shigella vaccine is likely to be available. | descriptive | | |

I

I

|  | 60 | [ d_bk_priority_multi]  Show the field ONLY if: [interview_location] = 1 | 4.4 In Burkina Faso, Shigella is responsible for an estimated 15,400 cases of moderate or severe stunting each year in children under five. Earlier, you considered Shigella vaccine a [d_bk_vaccine_priority] when you considered the burden of Shigella disease and impact of a vaccine. Now consider that in addition to the impact on moderate to  severe Shigella diarrhea deaths, a Shigella vaccine could prevent 8,400 cases of stunting in children under five each year in Burkina Faso. With this added benefit, what is the priority you would place on introducing  this Shigella vaccine in Burkina Faso, knowing that it will not be the only vaccine your country considers in 2025- 2030? | radio, Required | | |
| --- | --- | --- | --- | --- | --- | --- |
|  |  |  |  | 1 | High priority |  |
|  |  |  |  | 2 | Medium priority |  |
|  |  |  |  | 3 | Low priority |  |
|  |  |  |  | 4  - | Not a priority |  |
|  |  |  |  |  | | |
|  | 61 | [ d_ghana_priority_multi]  Show the field ONLY if: [interview_location] = '2' | 4.4 In Ghana, Shigella is responsible for an estimated 12,700 cases of moderate or severe stunting each year in children under five. Earlier, you considered Shigella vaccine a [d_ghana_vaccine_priority] when you considered the burden of Shigella disease and impact of a vaccine. Now consider that in addition to the impact on moderate to severe Shigella diarrhea deaths, a Shigella vaccine could prevent 7,000 cases of stunting in children under five each year in Ghana. With this added benefit, what is the priority you would place on introducing this Shigella vaccine in Ghana, knowing that it will not be the only vaccine your country considers in 2025-2030? | radio, Required | | |
|  |  |  |  | 1 | High priority |  |
|  |  |  |  | 2 | Medium priority |  |
|  |  |  |  | 3 | Low priority |  |
|  |  |  |  | 4 | Not a priority |  |
|  |  |  |  |  | | |
|  | 62 | [ d_kenya_priority_multi]  Show the field ONLY if: [interview_location] = '3' | 4.4 In Kenya, Shigella is responsible for an estimated 28,800 cases of moderate or severe stunting each year in children under five. Earlier, you considered Shigella vaccine a [d_kenya_vaccine_priority] when you considered the burden of Shigella disease and impact of a vaccine. Now consider that in addition to the impact on moderate to severe Shigella diarrhea deaths, a Shigella vaccine could prevent 15,400 cases of stunting in children under five each year in Kenya. With this added benefit, what is the priority you would place on introducing this Shigella vaccine in Kenya, knowing that it will not be the only vaccine your country considers in 2025-2030? | radio, Required | | |
|  |  |  |  | 1 | High priority |  |
|  |  |  |  | 2 | Medium priority |  |
|  |  |  |  | 3 | Low priority |  |
|  |  |  |  | 4 | Not a priority |  |
|  |  |  |  |  | | |
|  | 63 | [ d_nepal_priority_multi]  Show the field ONLY if: [interview_location] = '4' | 4.4 In Nepal, Shigella is responsible for an estimated 9,200 cases of moderate or severe stunting each year in children under five. Earlier, you considered Shigella vaccine a [d_nepal_vaccine_priority] when you considered the burden of Shigella disease and impact of a vaccine. Now consider that in addition to the impact on moderate to severe Shigella diarrhea deaths, a Shigella vaccine could prevent 4,800 cases of stunting in children under five each year in Nepal. With this added benefit, what is the priority you would place on introducing this Shigella vaccine in Nepal, knowing that it will not be the only vaccine your country considers in 2025-2030? | radio, Required | | |
|  |  |  |  | 1 | High priority |  |
|  |  |  |  | 2 | Medium priority |  |
|  |  |  |  | 3 | Low priority |  |
|  |  |  |  | 4 | Not a priority |  |
|  |  |  |  |  | | |
|  | 64 | [ d_vietnam_priority_multi]  Show the field ONLY if: [interview_location] = '5' | 4.4 In Vietnam, Shigella is responsible for an estimated 900 cases of moderate or severe stunting each year in children under five. Earlier, you considered Shigella vaccine a [d_vietnam_vaccine_priority] when you considered the burden of Shigella disease and impact of a vaccine. Now consider that in addition to the impact on moderate to severe Shigella diarrhea deaths, a Shigella vaccine could prevent 530 cases of stunting in children under five each year in Vietnam. With this added benefit, what is the priority you would place on introducing this  Shigella vaccine in Vietnam, knowing that it will not be the only vaccine your country considers in 2025-2030? | radio, Required | | |
|  |  |  |  | 1 | High priority |  |
|  |  |  |  | 2 | Medium priority |  |
|  |  |  |  | 3 | Low priority |  |
|  |  |  |  | 4 | Not a priority |  |
|  |  |  |  |  | | |
| 65  I | | [ d_priority_multi_why] | 4.4.1 Why? *Do not need to type the answer - audio recording will capture. | text | | |

|  | 66 | [ d_bk_priority_wageJ  Show the field ONLY if: [interview_location] = 1 | 4.5 Earlier, you considered Shigella vaccine a [d_bk_priority_multi] when you considered its impact on preventing mild to severe diarrhea, Shigella deaths, and grow1h stunting. Now consider that in addition to this, the vaccine could have a positive impact on wage earnings of adults. With this added benefit, what is the priority you would place on introducing this Shigella vaccine in [interview_location], knowing that it will not be the only vaccine your country considers in 2025-2030? | radio, Required | | | |
| --- | --- | --- | --- | --- | --- | --- | --- |
|  |  |  |  | 1 | High priority |  | |
|  |  |  |  | 2 | Medium priority |  |  |
|  |  |  |  | 3 | Low priority |  |  |
|  |  |  |  | 4  - | Not a priority |  |  |
|  |  |  |  |  | | | |
|  | 67 | [ d_ghana_priority_wage]  Show the field ONLY if: [interview_location] = '2' | 4.5 Earlier, you considered Shigella vaccine a [d_ghana_priority_multi] when you considered its impact on preventing mild to severe diarrhea, Shigella deaths, and grow1h stunting. Now consider that in addition to this, the vaccine could have a positive impact on wage earnings of adults. With this added benefit, what is the priority you would place on introducing this Shigella vaccine in [interview_location], knowing that it will not be the only vaccine your country considers in 2025-2030? | radio, Required | | | |
|  |  |  |  | 1 | High priority |  | |
|  |  |  |  | 2 | Medium priority |  |  |
|  |  |  |  | 3 | Low priority |  |  |
|  |  |  |  | 4 | Not a priority |  |  |
|  |  |  |  |  | | | |
|  | 68 | [ d_kenya_priority_wage]  Show the field ONLY if: [interview_location] = '3' | 4.5 Earlier, you considered Shigella vaccine a [d_kenya_priority_multi] when you considered its impact on preventing mild to severe diarrhea, Shigella deaths, and grow1h stunting. Now consider that in addition to this, the vaccine could have a positive impact on wage earnings of adults. With this added benefit, what is the priority you would place on introducing this Shigella vaccine in [interview_location], knowing that it will not be the only vaccine your country considers in 2025-2030? | radio, Required | | | |
|  |  |  |  | 1 | High priority |  | |
|  |  |  |  | 2 | Medium priority |  |  |
|  |  |  |  | 3 | Low priority |  |  |
|  |  |  |  | 4 | Not a priority |  |  |
|  |  |  |  |  | | | |
|  | 69 | [ d_nepal_priority_wage]  Show the field ONLY if: [interview_location] = '4' | 4.5 Earlier, you considered Shigella vaccine a [d_nepal_priority_multi] when you considered its impact on preventing mild to severe diarrhea, Shigella deaths, and grow1h stunting. Now consider that in addition to this, the vaccine could have a positive impact on wage earnings of adults. With this added benefit, what is the priority you would place on introducing this Shigella vaccine in [interview_location], knowing that it will not be the only vaccine your country considers in 2025-2030? | radio, Required | | | |
|  |  |  |  | 1 | High priority |  | |
|  |  |  |  | 2 | Medium priority |  |  |
|  |  |  |  | 3 | Low priority |  |  |
|  |  |  |  | 4 | Not a priority |  |  |
|  |  |  |  |  | | | |
|  | 70 | [ d_vietnam_priority_wage]  Show the field ONLY if: [interview_location] = '5' | 4.5 Earlier, you considered Shigella vaccine a [d_vietnam_priority_multi] when you considered its impact on preventing mild to severe diarrhea, Shigella deaths, and grow1h stunting. Now consider that in addition to this, the vaccine could have a positive impact on wage earnings of adults. With this added benefit, what is the priority you would place on introducing this Shigella vaccine in [interview_location], knowing that it will not be the only  vaccine your country considers in 2025-2030? | radio, Required | | | |
|  |  |  |  | 1 | High priority |  | |
|  |  |  |  | 2 | Medium priority |  |  |
|  |  |  |  | 3 | Low priority |  |  |
|  |  |  |  | 4 | Not a priority |  |  |
|  |  |  |  |  | | | |
| 171 | | [why_wage_earning_priority] | 4.5.1 Why?*Do not need to type the answer - audio recording will capture. | text, Required | | | |
|  | 72 | [ d_description_4 J  Show the field ONLY if: [interview_level] = '1' | Shigella is only one cause of diarrhea and if the vaccine's impact on preventing diarrhea is considered alone, it may be less cost-effective as a diarrhea control measure compared to rotavirus vaccine and other nutritional interventions such as Vitamin A supplementation.  However, if the additional long-term health, development, and economic benefits of a potential Shigella vaccine are included, the overall cost effectiveness of the vaccine improves and, in some instances, the vaccine might be cost saving. | descriptive | | | |
|  | 73 | [ d_vaccine_benefits]  Show the field ONLY if: [interview_level] = '1' | 4.6 To what degree do these additional benefits of a Shigella vaccine and their potential to improve the cost effectiveness or cost savings of the vaccine make you more likely to prioritize the vaccine? | radio, Required | | | |
|  |  |  |  | 2 | Much more likely | |  |
|  |  |  |  | 1 | Moderately more likely | |  |
|  |  |  |  | 0 | Would not affect interest | |  |
|  | 74 |  |  |  |  |  |  |
|  |  | [ d_vaccine_benefits_why]  Show the field ONLY if: [interview_level] = '1' | 4.6.1 Why? *Do not need to type the answer - audio recording will capture. | text | | | |

I

- - - 1. [ d_specific_price]

Show the field ONLY if: [interview_level] = '1'

- - - 1. [visual_aid_5_bk]

Show the field ONLY if: [interview_location] = 1

- - - 1. [ e_bk_admin_time]

Show the field ONLY if: [interview_location] = 1

- - - 1. [visual_aid_5_ghana]

Show the field ONLY if: [interview_location] = '2'

- - - 1. [ e_ghana_admin_time]

Show the field ONLY if: [interview_location] = '2'

- - - 1. [visual_aid_5_kenya]

Show the field ONLY if: [interview_location] = '3'

- - - 1. [ e_kenya_admin_time]

Show the field ONLY if: [interview_location] = 3

- - - 1. [visual_aid_5_nepal]

Show the field ONLY if: [interview_location] = '4'

- - - 1. [ e_nepal_admin_time]

Show the field ONLY if: [interview_location] = 4

- - - 1. [visual_aid_5_vietnam]

Show the field ONLY if: [interview_location] = '5'

- - - 1. [ e_vietnam_admin_time]

Show the field ONLY if: [interview_location] = 5

4.7 Is there a specific price point, as in cost per dose or fully vaccinated child, beyond which you would not be in favor of adding a Shigella vaccine to the routine immunization schedule? If so, what is the price point? What factors do you take into consideration when you are making such a decision? *Do not need to type the answer - audio recording will capture.

Section Header: *Section E: Vaccine Attributes I wont you* to *imagine* thot *[interview_/ocotion] is considering introducing* o *Shigel/o vaccine. As* o *reminder, the vaccine is expected* to *require 1 or* 2 *doses for the primary series, given mid-* to /ate- *in the first year of life. For these next questions, I am interested in your preference for specific vaccine attributes, with a* ***focus on the vaccine schedule, single antigen versus combination vaccines,*** *and the route of administration.*

For investigators only: Please refer to visual aid #5 for question 5.1.

5.1 I want to start by asking you about the vaccine schedule. Assume Shigella vaccine is a single antigen vaccine given by injection, that requires ONE DOSE for the primary series at either 6 MONTHS, which requires a new vaccine visit, or 9 MONTHS when MR and yellow fever vaccines are already administered. Operationally, what administration time point do you prefer?

For investigators only: Please refer to visual aid #5 for question 5.1.

5.1 I want to start by asking you about the vaccine schedule. Assume Shigella vaccine is a single antigen vaccine given by injection, that requires ONE DOSE for the primary series at either 6 MONTHS, which requires a new vaccine visit, or 9 MONTHS when MR and yellow fever vaccines already administered. Operationally, what administration time point do you prefer?

For investigators only: Please refer to visual aid #5 for question 5.1.

5.1 I want to start by asking you about the vaccine schedule. Assume Shigella vaccine is a single antigen vaccine given by injection, that requires ONE DOSE for the primary series at either 6 MONTHS, which requires a new vaccine visit, or 9 MONTHS when **MR** and yellow fever vaccines already administered. Operationally, what administration time point do you prefer?

For investigators only: Please refer to visual aid #5 for question 5.1.

5.1 I want to start by asking you about the vaccine schedule. Assume Shigella vaccine is a single antigen vaccine given by injection, that requires ONE DOSE for the primary series at either 6 MONTHS, which requires a new vaccine visit, or 9 MONTHS when **MR** vaccine already administered. Operationally, what administration time point do you prefer?

For investigators only: Please refer to visual aid #5 for question 5.1.

5.1 I want to start by asking you about the vaccine schedule. Assume Shigella vaccine is a single antigen vaccine given by injection, that requires ONE DOSE for the primary series at either 6 MONTHS, which requires a new vaccine visit, or 9 MONTHS when measles vaccine is already administered. Operationally, what administration time point do you prefer?

text

descriptive

radio, Required

| 1 | 6 months of age/ new vaccine visit |
| --- | --- |
| 2 | 9 months of age/ existing vaccine visit |
| 3  - | Either 6 or 9 months of age |

descriptive

radio, Required

| 1 | 6 months of age/ new vaccine visit |
| --- | --- |
| 2 | 9 months of age/ existing vaccine visit |
| 3 | Either 6 or 9 months of age |

descriptive

radio, Required

| 1 | 6 months of age/ new vaccine visit |
| --- | --- |
| 2 | 9 months of age/ existing vaccine visit |
| 3 | Either 6 or 9 months of age |

descriptive

radio, Required

| 1 | 6 months of age/ new vaccine visit |
| --- | --- |
| 2 | 9 months of age/ existing vaccine visit |
| 3 | Either 6 or 9 months of age |

descriptive

radio, Required

| 1 | New vaccine visit |
| --- | --- |
| 2 | Existing vaccine visit |
| 3 | Either visit |

| 1 | Would not affect interest |
| --- | --- |
| 2 | Moderately less willing to consider this vaccine |
| 3 | Much less willing to consider this vaccine |
| 4  - | Would not consider this vaccine |

| 1 | Would not affect interest |
| --- | --- |
| 2 | Moderately less willing to consider this vaccine |
| 3 | Much less willing to consider this vaccine |
| 4 | Would not consider this vaccine |

| 86 | [ e_bk_admin_time_why]  Show the field ONLY if: [e_bk_admin_time]=1 or [e_bk  _admin_time]=2 or [e_bk_adm in_time]=3 and [interview_loc ation] = 1 | 5.1.1 Why did you select '(e_bk_admin_time]'? *Do not need to type the answer - audio recording will capture. | text |
| --- | --- | --- | --- |
| 87 | [ e_ghana_admin_time_why J  Show the field ONLY if: [e_ghana_admin_time]=1 or [e_ghana_admin_time]=2 or [e_ghana_admin_time] = 3 an d [interview_location] = 2 | 5.1.1 Why did you select '[e_ghana_admin_time]'?*Do not need to type the answer - audio recording will capture. | text |
| 88 | [ e_kenya_admin_time_why]  Show the field ONLY if: [e_kenya_admin_time]=1 or [e  _kenya_admin_time]=2 or [e_k enya_admin_time]=3 and [int erview_location]=3 | 5.1.1 Why did you select '(e_kenya_admin_time]'?*Do not need to type the answer - audio recording will capture. | text |
| 89 | [ e_nepal_admin_time_why]  Show the field ONLY if: [e_nepal_admin_time]=1 or [e  _nepal_admin_time]=2 or [e_n epal_admin_time]=3 and [inte rview_location] = 4 | 5.1.1 Why did you select '[e_nepal_admin_time]'?*Do not need to type the answer - audio recording will capture. | text |
| 90 | [e_vietnam_admin_time_why  l  Show the field ONLY if: [e_vietnam_admin_time]=1 or [e_vietnam_admin_time]=2 or [e_vietnam_admin_time]=3 an d [interview_location]=5 | 5.1.1 Why did you select '[e_vietnam_admin_time]'? *Do not need to type the answer - audio recording will capture. | text |
| 91 | [ e_admin_time_6mo J  Show the field ONLY if: [e_bk_admin_time] = '2' or [e_ ghana_admin_time] = '2' or [e  _kenya_admin_time] = '2' or [e_nepal_admin_time] = '2' or [e_vietnam_admin_time] = '2' | 5.2 I now want you to imagine that the single antigen, injectable vaccine can only be offered at a new vaccine visit. How would this affect your willingness to consider introducing a Shigella vaccine as part of the routine immunization schedule? | radio, Required |
| 92 | [ e_admin_time_9mo]  Show the field ONLY if: [e_bk_admin_time] = '1' or [e_ ghana_admin_time] = '1' or [e  _kenya_admin_time] = '1' or [e_nepal_admin_time] = '1' or [e_vietnam_admin_time] = '1' | 5.2 I now want you to imagine that the single antigen, injectable vaccine can only be offered at an existing vaccine visit. How would this affect your willingness to consider introducing a Shigella vaccine as part of the routine immunization schedule? | radio, Required |
| 93 | [ e_admin_time_notpref_why]  Show the field ONLY if: [e_admin_time_6mo] = '1' or [e_admin_time_6mo] = '2' or [e_admin_time_6mo] = '3' or [e_admin_time_6mo] = '4' | 5.2.2 Why did you select '(e_admin_time_6mo]'7*Do not need to type the answer - audio recording will capture. | text |
| 94 | [ e_admin_time_notpref_why2  J  Show the field ONLY if: [e_admin_time_9mo] = '1' OR [e_admin_time_9mo] = '2' OR [e_admin_time_9mo] = '3' OR [e_admin_time_9mo] = '4' | 5.2.2 Why did you select '[e_admin_time_9mo]'?*Do not need to type the answer - audio recording will capture. | text |

I

|  | 95 | [ e_two_doses] | 5.3 Let's now suppose that the single antigen, injectable Shigella vaccine requires TWO DOSES for the primary series. How would the requirement of a TWO-DOSE primary series schedule affect your willingness to consider introducing a Shigella vaccine as part of the routine immunization schedule? | radio, Required | | | |
| --- | --- | --- | --- | --- | --- | --- | --- |
|  |  |  |  | 1 | Would not affect interest | |  |
|  |  |  |  | 2 | Moderately less willing to consider this vaccine | |  |
|  |  |  |  | 3 | Much less willing to consider this vaccine | |  |
|  |  |  |  | 4  - | Would not consider this vaccine | |  |
| 96  I | | [ e_two_doses_why] | 5.3.1 Why did you select '[e_two_doses]'? *Do not need to type the answer - audio recording will capture. | text | | | |
|  | 97 | [visual_aid_6_bk]  Show the field ONLY if: [interview_location] = 1 | For investigators only: Please refer to visual aid #6 for question 5.4. | descriptive | | | |
|  | 98 | [visual_aid_6_ghana]  Show the field ONLY if: [interview_location] = '2' | For investigators only: Please refer to visual aid #6 for question 5.4. | descriptive | | | |
|  | 99 | [visual_aid_6_kenya]  Show the field ONLY if: [interview_location] = '3' | For investigators only: Please refer to visual aid #6 for question 5.4. | descriptive | | | |
|  | 100 | [visual_aid_6_nepal]  Show the field ONLY if: [interview_location] = '4' | For investigators only: Please refer to visual aid #6 for question 5.4. | descriptive | | | |
|  | 101 | [visual_aid_6_vietnam]  Show the field ONLY if: [interview_location] = '5' | For investigators only: Please refer to visual aid #6 for question 5.4. | descriptive | | | |
|  | 102 | [ e_single_dose_inject] | 5.4 Let's suppose that [interview_location] has decided to provide an injectable, single dose primary series Shigella vaccine at 9 months of age into the routine immunization schedule. The vaccine is available in either a single antigen presentation or as part of a combination vaccine, in which Shigella is paired with another antigen in a single injection. Which vaccine presentation would you prefer? | radio, Required | | | |
|  |  |  |  | 1 | A single antigen vaccine (Shigella only) | | |
|  |  |  |  | 2 | A combination vaccine (Shigella plus another antigen) | | |
|  |  |  |  | 0 | No preference between single and combination vaccines | | |
| 1103 | | [ e_single_dose_inject_why] | 5.4.1 Why did you select '[e_single_dose_inject]'? *Do not need to type the answer - audio recording will capture. | text | | | |
|  | 104 | [ stand_alone_cascade]  Show the field ONLY if: [e_single_dose_inject] = 1 | 5.5 Which of the following attributes would change your willingness to consider introducing a single antigen presentation Shigella vaccine. Select all that apply. | checkbox, Required | | | |
|  |  |  |  | 1 | stand_alone_cascade- 1 | The single antigen vaccine is moderately less effective at preventing moderate to severe diarrhea and/or deaths compared to the combination vaccine | |
|  |  |  |  | 2 | stand_alone_cascade- 2 | The single antigen vaccine requires moderately more 2- 8°C cold chain space compared to the combination vaccine | |
|  |  |  |  | 3 | stand_alone_cascade_3 | The single antigen vaccine is moderately more expensive compared to the  combination vaccine | |
|  |  |  |  | stand_alone_cascade_4 | | None of the above | |

I

| 1 | Would not affect interest |
| --- | --- |
| 2 | Moderately less willing to consider this vaccine |
| 3 | Much less willing to consider this vaccine |
| 4  - | Would not consider this vaccine |

| 1 | Would not affect interest |
| --- | --- |
| 2 | Moderately less willing to consider this vaccine |
| 3 | Much less willing to consider this vaccine |
| 4 | Would not consider this vaccine |

| 105 | [ combo_cascade]  Show the field ONLY if: [e_single_dose_inject] = 2 | 5.5 Which of the following attributes would change your willingness to consider introducing a combination presentation Shigella vaccine? Select all that apply. | checkbox, Required | | |
| --- | --- | --- | --- | --- | --- |
|  |  |  |  | combo_cascade | The combination vaccine is moderately less effective at preventing moderate to severe diarrhea and/or deaths compared to the single antigen vaccine |
|  |  |  | 2 | combo_cascade 2 | The combination vaccine requires moderately more 2-8°( cold chain space compared to the single antigen vaccine |
|  |  |  | 3 | combo_cascade_3 The combination vaccine is | |
|  |  |  |  |  | moderately more expensive compared to  the single antigen vaccine |
|  |  |  | 4 combo_cascade_4 | | None of the above |
| 106 | [ stand_alone_cascade_why]  Show the field ONLY if: [stand_alone_cascade(1)] = '1' or [stand_alone_cascade(2)] = '1' or [stand_alone_cascade  (3)] = '1' or [stand_alone_casc ade(4)] = '1' or [combo_casca de(1)] = '1' or [combo_cascad e(2)] = '1' or [combo_cascade (3)] = '1' or [combo_cascade  (4)] = '1' | 5.5.1 Why? *Do not need to type the answer - audio recording will capture. | text | | |
| 107 | [ e_combo_pref]  Show the field ONLY if: [e_single_dose_inject] = '2' | 5.6 Now imagine that the Shigella vaccine is only offered as single antigen vaccine (Shigella only). How would this affect your willingness to consider introducing a Shigella vaccine as part of the routine immunization schedule? | radio, Required | | |
| 108 | [ e_combo_pref_2]  Show the field ONLY if: [e_single_dose_inject] = '1' | 5.6 Now imagine that the Shigella vaccine is only offered as a combination vaccine (Shigella plus another antigen). How would this affect your willingness to consider introducing  a Shigella vaccine as part of the routine immunization schedule? | radio, Required | | |
| 109 | [ e_combo_pref_why]  Show the field ONLY if: [e_combo_pref] = '1' or [e_co mbo_pref] = '2' or [e_combo_ pref] = '3' or [e_combo_pref] =  '4' | 5.6.1 Why did you select '(e_combo_pref]'? *Do not need to type the answer - audio recording will capture. | text | | |
| 110 | [ e_combo_pref_2_why]  Show the field ONLY if: [e_combo_pref_2] = '1' or [e_c ombo_pref_2] = '2' or [e_com bo_pref_2] = '3' or [e_combo_  pref_2] = '4' | 5.6.1 Why did you select '[e_combo_pref_2]'? *Do not need to type the answer - audio recording will capture. | text | | |
| 111 | [visual_aid_7_bk]  Show the field ONLY if: [interview_location] = 1 | For investigators only: Please refer to visual aid #7 for question 5.7. | descriptive | | |
| 112 | [visual_aid_7_ghana]  Show the field ONLY if: [interview_location] = 2 | For investigators only: Please refer to visual aid #7 for question 5.7. | descriptive | | |
| 113 | [visual_aid_7_kenya]  Show the field ONLY if: [interview_location]=3 | For investigators only: Please refer to visual aid #7 for question 5.7. | descriptive | | |

| 114 | [visual_aid_7_nepal]  Show the field ONLY if: [interview_location] = 4 | For investigators only: Please refer to visual aid #7 for question 5.7. | descriptive | | | |
| --- | --- | --- | --- | --- | --- | --- |
| 115 | [visual_aid_7_vietnam]  Show the field ONLY if: [interview_location]=5 | For investigators only: Please refer to visual aid #7 for question 5.7. | descriptive | | | |
| 116 | [ e_route_admin_only] | 5.7 Let's suppose that [interview_location] has decided to provide a single dose primary series Shigella vaccine at 9 months of age. The vaccine can either be administered orally or parenterally through injection. Which route of administration would you prefer? | radio, Required | | | |
| 117 | [ e_route_admin_only_why]  Show the field ONLY if: [e_route_admin_only] = '1' or [e_route_admin_only] = '2' or [e_route_admin_only] = '3' | 5.7.1 Why did you select '[e_route_admin_only]'?*Do not need to type the answer - audio recording will capture. | text | | | |
| 118 | [ e_oral_cascade]  Show the field ONLY if: [e_route_admin_only]=1 | 5.8 Which of the attributes below would change your willingness to consider an oral Shigella vaccine? Select all that apply. | checkbox, Required | | | |
|  |  |  |  | e_oral_cascade_1 | The oral vaccine is moderately less effective at preventing moderate to severe diarrhea and/or deaths compared to the injectable vaccine | |
|  |  |  | 2 | e_oral_cascade_2 | The oral vaccine requires moderately more 2-8°C cold chain space compared to the injectable vaccine | |
|  |  |  | 3 | e_oral_cascade_3 The oral vaccine is | | |
|  |  |  |  |  | moderately more expensive  compared to the injectable vaccine | |
|  |  |  | 4 e_oral_cascade_4 | | None of the above | |
| 119 | [ e_injectable_cascade]  Show the field ONLY if: [e_route_admin_only]=2 | 5.8 Which of the attributes below would change your willingness to consider an injectable Shigella vaccine? Select all that apply. | checkbox, Required | | | |
|  |  |  | 1 | e_injectable_cascade_1 | | The injectable vaccine is moderately less effective at preventing moderate to severe diarrhea and/or deaths compared to the oral vaccine |
|  |  |  | 2 | e_injectable_cascade_2 | | The injectable vaccine requires moderately more 2-8°C cold chain space compared to the oral vaccine |
|  |  |  | 3 | e_injectable_cascade_3 | | The injectable vaccine is moderately more expensive compared to the oral vaccine |
|  |  |  | 4 e_injectable_cascade_4 | | | None of the above |
| 120 | [ e_oral_injectable_cascade_w hy]  Show the field ONLY if: [e_oral_cascade(1)] = '1' or [e_ oral_cascade(2)] = '1' or [e_ora l_cascade(3)] = '1' or [e_oral_c ascade(4)] = '1' or [e_injectabl e_cascade(1)] = '1' or [e_inject able_cascade(2)] = '1' or [e_inj ectable_cascade(3)] = '1' or [e  _injectable_cascade(4)] = '1' | 5.8.1 Why?*Do not need to type the answer- audio recording will capture. | text | | | |

| 1 | Oral vaccine |
| --- | --- |
| 2 | Injectable vaccine |
| 3  - | No preference between oral and injectable vaccines |

|  | 121 | [ e_oral_only]  Show the field ONLY if: [e_route_admin_only] = 2 | 5.9 Now imagine that the Shigella vaccine is only offered as an oral vaccine. How would this affect your willingness to consider introducing a Shigella vaccine as part ofthe routine immunization schedule? | radio, Required | | |
| --- | --- | --- | --- | --- | --- | --- |
|  |  |  |  | 1 | Would not affect interest |  |
|  |  |  |  | 2 | Moderately less willing to consider this vaccine |  |
|  |  |  |  | 3 | Much less willing to consider this vaccine |  |
|  |  |  |  | 4  - | Would not consider this vaccine |  |
|  | 122 | [ e_injectable_only]  Show the field ONLY if: [e_route_admin_only] = 1 | 5.9 Now imagine that the Shigella vaccine is only offered as an injectable vaccine. How would this affect your willingness to consider introducing a Shigella vaccine as part of the routine immunization schedule? | radio, Required | | |
|  |  |  |  | 1 | Would not affect interest |  |
|  |  |  |  | 2 | Moderately less willing to consider this vaccine |  |
|  |  |  |  | 3 | Much less willing to consider this vaccine |  |
|  |  |  |  | 4 | Would not consider this vaccine |  |
|  | 123 |  |  |  | | |
|  |  | [e_route_admin_only_2_why]  Show the field ONLY if: [e_oral_only] = '1' or [e_oral_o nly] = '2' or [e_oral_only] = '3' or [e_oral_only] = '4' | 5.9.1 Why did you select '[e_oral_only]'? *Do not need to type the answer - audio recording will capture. | text | | |
|  | 124 | [ e_route_admin_only_2_why_ 2]  Show the field ONLY if: [e_injectable_only] = '1' or [e_i njectable_only] = '2' or [e_inje ctable_only] = '3' or [e_injecta ble_only] = '4' | 5.9.1 Why did you select '[e_injectable_only]'? *Do not need to type the answer - audio recording will capture. | text | | |
|  | 125 | [ e_description_2] | There are several other attributes of a hypothetical Shigella vaccine that could have operational implications to its delivery. Imagine that [interview_location] is considering introducing a hypothetical Shigella vaccine that is a single antigen, injectable vaccine, with a primary series given as a single dose, delivered at either 6 or 9 months of age. | descriptive | | |
|  | 126 | [ e_lyophilized l | 5.10 The vaccine is lyophilized and requires re-constitution before use. How would this affect your willingness to consider introducing a Shigella vaccine as part of the routine immunization schedule? | radio, Required | | |
|  |  |  |  | 1 | Would not affect interest |  |
|  |  |  |  | 2 | Moderately less willing to consider this vaccine |  |
|  |  |  |  | 3 | Much less willing to consider this vaccine |  |
|  |  |  |  | 4 | Would not consider this vaccine |  |
| 1127 | | [ e_lyophilized_why] | 5.10.1 Why did you select '[e_lyophilized]'? *Do not need to type the answer - audio recording will capture. | text | | |
|  | 128 | [ e_packaged_single_dose] | 5.11 The vaccine is only available packaged in single dose presentation (i.e., pre-filled syringes). How would this affect your willingness to consider introducing a Shigella vaccine as part of the routine immunization schedule? | radio, Required | | |
|  |  |  |  | 1 | Would not affect interest |  |
|  |  |  |  | 2 | Moderately less willing to consider this vaccine |  |
|  |  |  |  | 3 | Much less willing to consider this vaccine |  |
|  |  |  |  | 4 | Would not consider this vaccine |  |
| 1129 | | [ e_packaged_single_dose_wh y] | 5.11.1 Why did you select '[e_packaged_single_dose]'? *Do not need to type the answer - audio recording will capture. | text | | |
|  | 130 | [ e_booster _year_2] | 5.12 The vaccine may require a booster dose given in the 2nd year of life. How would this affect your willingness to consider introducing a Shigella vaccine as part of the routine immunization schedule? | radio, Required | | |
|  |  |  |  | 1 | Would not affect interest |  |
|  |  |  |  | 2 | Moderately less willing to consider this vaccine |  |
|  |  |  |  | 3 | Much less willing to consider this vaccine |  |
|  |  |  |  | 4 | Would not consider this vaccine |  |
| 1131 | | [ e_booster _year_2_why] | 5.12.1 Why did you select '[e_booster_year_2]'? *Do not need to type the answer - audio recording will capture. | text | | |
|  | 132 | [ e_storage_neg_20] | 5.13 The vaccine requires storage at or below -20°C. How would this affect your willingness to consider introducing a Shigella vaccine as part of the routine immunization schedule? | radio, Required | | |
|  |  |  |  | 1 | Would not affect interest |  |
|  |  |  |  | 2 | Moderately less willing to consider this vaccine |  |
|  |  |  |  | 3 | Much less willing to consider this vaccine |  |
|  |  |  |  | 4 | Would not consider this vaccine |  |
|  |  |  |  |  |  |  |

I

I

I

1133

I

134

135

I 1136

137

138

139

140

[ e_storage_neg_20_why] [ e_other_attributes]

[f_sub_nat_intro]

[why_do_not_need_to_type_t h]

[ f_sub_nat_acce ptability]

[f_sub_nat_feasibility]

[g_other_comments]

[ interview_guide_english_co mplete]

5.13.1 Why did you select '[e_storage_neg_20]'? *Do not need to type the answer - audio recording will capture.

5.14 Are there any other attributes that would affect your willingness to consider a Shigella vaccine as part of the routine immunization schedule? What attributes? Why?

*Do not need to type the answer - audio recording will capture.

Section Header: *Section F: Sub-notional NV/ Preferences This is the lost section of our interview. For the last three questions, imagine that [interview_location] is found to have widely different Shigello incidence across geographies or is prone to annual Shigello outbreaks in certain areas of the country. Consider the possibility of introducing* o *Shigel/o vaccine sub-nationally, for instance in high-incidence areas only or as an outbreak control measure.*

- 1. Would you favor subnational introduction of a Shigella vaccine?
     1. Why?*Do not need to type the answer - audio recording will capture.
  2. What sort of acceptability issues do you foresee with providing a Shigella vaccine sub-nationally? *Do not need to type the answer - audio recording will capture.

*Probe: for policy makers, for health workers, for community members?*

- 1. What operational or delivery feasibility issues do you foresee with providing a Shigella vaccine sub-nationally?

*Do not need to type the answer - audio recording will capture.

*Probe: for policy makers, for health workers, for community members?*

Section Header: *Section G: Interview Closure*

- 1. Other than the topics already covered in this interview, is there anything else you'd like to say that would affect your interest in introducing a Shigella vaccine as part of the routine immunization schedule in your setting? *Do not need to type the answer - audio recording will capture.

Section Header: *Form Status*

Complete?

text text

radio, Required

0

text text

text

text

dropdown

| 0 | Incomplete |
| --- | --- |
| 1 | Unverified |
| 2 | Complete |

Ins-trument: **INTERVIEW GUIDE - FRENCH** (interview_guide_french)

- - 1. [ unique_id_french] X.0 Numero d'identification unique text, Required

- -- ---

- - 1. [ recording_confirmation_fren X.1 Avez-vous active l'enregistrement audio? radio, Required ch]

n

143

1144

I

145

I I 146

147

[ recording_sta rt_script_french

# l

[ interview_date_french]

[ interview_location_french]

[ city_town_district_french] [ interview_level_french]

- 1. Parlez dans l'appareil d'enregistrement: "Commencer l'entretien avec le repondant ayant l'ID unique [unique_id_french]"
  2. Date de l'entretien: jJ/MM/AAAA
  3. Pays dans lequel l'entretien est realise

X4.1 Norn de la ville/localite

- 1. lndiquez a quel niveau cet entretien a ete conduit

descriptive

text (date_dmy), Required Field Annotation: @TODAY

radio, Required

- - 1. Burkina Faso
    2. Ghana
    3. Kenya
    4. Nepal
    5. Vietnam

-

text, Required

radio, Required

1. Niveau national
2. Niveau de l'etablissement sanitaire (continuer ci- dessous)

|  | 148 | [ hcf_level_french]  Show the field ONLY if: [interview_level_french] = '2' | X.5.3 A quel niveau de structure sanitaire etes-vous? | radio, Required | | | |
| --- | --- | --- | --- | --- | --- | --- | --- |
|  |  |  |  | 1 | Niveau primaire |  | |
|  |  |  |  | 2 | Niveau secondaire |  |  |
|  |  |  |  | 3  - | Niveau tertiaire |  |  |
|  | 149 | [ hcf_publicvprivate_french]  Show the field ONLY if: [interview_level_french] = '2' | X.5.4 L'etablissement de sante est-ii public ou prive? | radio, Required | | | |
|  |  |  |  | 1 | Etablissement public | |  |
|  |  |  |  | 2 | Etablissement prive | |  |
|  | 150 |  |  |  | | | |
|  |  | [ hcf_name_french]  Show the field ONLY if: [interview_level_french] = '2' | X.5.5 Norn de la structure sanitaire | text, Required, Identifier | | | |
|  | 151 | [ a_org_french] | Section Header: *Section A: Profit des intervenants*  1.1 Pour quelle organisation travaillez-vous? *Do not need to type the answer - audio recording will capture. | text, Identifier | | | |
| 1152 | | [ a_title_french] | 1.2 Quel est votre titre/role? *Do not need to type the answer - audio recording will capture. | text, Identifier | | | |
|  | 153 | [ a_years_experience_french] | 1.3 Depuis combien d'annees occupez-vous votre paste actuel? *Do not need to type the answer - audio recording will capture. | text, Identifier | | | |
|  | 154 | [ a_nitag_icc_french]  Show the field ONLY if: [interview_level_french] = '1' o r [hcf_level_french] = '3' | 1.4 Etes-vous membre du NITAG (Groupe consultatif technique national sur la vaccination) ou du CCIA (Comite  de Coordination lnter-Agences) ou bien prenez-vous regulierement part a leurs reunions? | radio, Required  n | | | |
|  | 155 | [ b_u5_health_concerns_frenc h] | Section Header: *Section B: Priorites en matiere de sante et perception sur*  */es maladies diarrheiques*  2.1 Nous allons commencer par discuter de la sante des enfants au Burkina Faso. Pouvez-vous me parler des 2-3 principaux problemes de sante affectant les enfants de mains de cinq ans au [interview_location_french]? *Do not need to type the answer - audio recording will capture. | text | | | |
|  | 156 | [ b_u5_health_concern_french  l | 2.2 Quel est l'impact de la diarrhee sur les enfants de mains de cinq ans dans votre contexte/localite? | radio, Required | | | |
|  |  |  |  | 1 | Un probleme de sante tres serieux | | |
|  |  |  |  | 2 | Un probleme serieux, mais pas parmi les problemes majeurs de sante | | |
|  |  |  |  | 3  - | Pas aussi tres grave par rapport aux autres problemes de sante | | |
|  | 157 | [ b_u5_health_concern_why_fr ench] | 2.2.1 Pourquoi? *Do not need to type the answer - audio recording will capture.  *Question portant sur /es: sympt6mes aigus, sequel/es, impacts c/iniques,*  ***economiques ou sociaux*** | text | | | |
|  | 158 | [ b_interv_priority_french] | 2.3 Quelles sont les interventions que vous considerez comme vos premiere et deuxieme priorites en matiere de prevention de la diarrhee? *Do not need to type the answer - audio recording will capture.  *Question portant sur* le *vaccin (Rotavirus, Cholera}, l'eaulassainissement,*  *fa nutrition, le lavage des mains* | text | | | |
|  | 159 | [ b_importance_growth_frenc h] | 2.4 Quelle est !'importance du retard de croissance en tant que probleme de sante pour les enfants de mains de cinq ans au [interview_location_french]? | radio, Required | | | |
|  |  |  |  | 1 | Un probleme de sante tres serieux | | |
|  |  |  |  | 2 | Un probleme serieux, mais pas parmi les problemes majeurs de sante | | |
|  |  |  |  | 3 | Pas aussi tres grave par rapport aux autres problemes de sante | | |
|  | 160 |  |  |  | | | |
|  |  | [ b_importance_growth_why_f rench] | 2.4.1 Pourquoi? *Do not need to type the answer - audio recording will capture.  *Question portant sur /es: sympt6mes aigus, sequel/es, impacts cliniques*  ***au sociaux.*** | text | | | |
|  | 161 | [ b_heard_of_shigella_french] | 2.5 Avez-vous entendu parler de Shigella? | radio, Required  n | | | |

I

|  | 162 | [shigella_awareness_french J  Show the field ONLY if: [b_heard_of_shigella_french]  = '1' | 2.6 Dites-moi ce que vous avez appris sur Shigella. *Do not need to type the answer - audio recording will capture.  *Question portant sur: fardeau de la maladielpopulation a haut risque;*  ***source de transmission, diagnostic, sympt6mes, sequel/es et traitement*** | text | | | | |
| --- | --- | --- | --- | --- | --- | --- | --- | --- |
|  | 163 | [ b_shigella_importance_frenc h]  Show the field ONLY if: [b_heard_of_shigella_french]  = '1' | 2.7 Comment voyez-vous !'importance de Shigella comme probleme de sante pour les enfants de mains de 5 ans dans votre contexte/localite? | radio, Required | | | | |
|  |  |  |  | 1 | | Un tres serieux probleme de sante | | |
|  |  |  |  | 2  -  3 | | Un serieux probleme de sante, mais pas parmi les problemes les plus importants | | |
|  |  |  |  |  |  | Pas un serieux probleme de sante comparativement aux autres problemes de sante | | |
|  |  |  |  | Ne sait pas | | | | |
|  | 164 | [ b_shigella_aspects_french J  Show the field ONLY if: [b_heard_of_shigella_french]  = '1' | 2.8 Quels sont les aspects de Shigella qui vous preoccupent le plus? *Do not need to type the answer - audio recording will capture.  *Question portant sur /'importance de la diarrhee (sympt6mes aigus}, du*  *retard de croissance (sequel/es) et des impacts cliniques ou sociaux* | text | | | | |
|  | 165 | [ b_shigella_priority_frenchJ  Show the field ONLY if: [b_heard_of_shigella_french]  = '1' | 2.9 Considerons qu'un vaccin anti Shigella est disponible, quelle priorite accorderiez-vous a !'introduction d'un vaccin  anti Shigella au [interview_location_french] au profit des jeunes enfants? | radio, Required | | | | |
|  |  |  |  | 1 | Priorite absolue | | |  |
|  |  |  |  | 2 | Priorite moyenne | | |  |
|  |  |  |  | 3 | Priorite faible | | |  |
|  |  |  |  | 4 | Pas une priorite | | |  |
|  | 166 |  |  |  | | | | |
|  |  | [ c_amr_importance_french J | Section Header: *Section C: Resistance antimicrobienne Nous savons que*  ***la "resistance aux antimicrobiens" est une preoccupation croissante par***  *rapport a de nombreux agents pathogenes dons le monde.*  3.1 Quelle est !'importance de la resistance aux antimicrobiens en tant que probleme de sante dans votre contexte/localite? | radio, Required | | | | |
|  |  |  |  | 3 | Un probleme de sante tres serieux | | | |
|  |  |  |  | 2 | Un probleme serieux, mais pas parmi les problemes majeurs de sante | | | |
|  |  |  |  | 1 | Pas un probleme tres grave par rapport aux autres problemes de sante | | | |
|  |  |  |  | 0 | Ne sait pas | | | |
| 1167 | | [ c_amr_importance_why_fren ch] | 3.1.1 Pourquoi? *Do not need to type the answer - audio recording will capture. | text | | | | |
|  | 168 | [ c_amr_treatment_costs_fren ch]  Show the field ONLY if: [interview_level_french] = '2' | 3.2 La resistance aux antimicrobiens affecte-t-elle ou interfere-t-elle sur le traitement administre par les agents de sante contre la diarrhee sanglante / dysenterie au [interview_location_french]? | radio, Required | | | | |
|  |  |  |  | 1 | | Oui |  | |
|  |  |  |  | 0 | | Non |  |  |
|  |  |  |  | 99 | | Ne sais pas |  |  |
|  | 169 |  |  |  | | | | |
|  |  | [ c_amr_treatment_costs_yes_ french J  Show the field ONLY if:  [ c_amr_treatment_costs_frenc h] = '1' | 3.2.1 Comment? *Do not need to type the answer - audio recording will capture. | text | | | | |
|  | 170 | [ c_amr_ns_priority_french J  Show the field ONLY if: [interview_level_french] = '1' | 3.3 Les informations sur la resistance antimicrobienne d'un agent pathogene cible sont-elles prises en compte lors de la formulation des priorites en matiere d'introduction d'un nouveau vaccin contre cet agent pathogene au [interview_location_french]? | radio, Required | | | | |
|  |  |  |  | 1 | | Qui |  | |
|  |  |  |  | 0 | | Non |  |  |
|  |  |  |  | 99 | | Ne sais pas |  |  |
|  | 171 |  |  |  |  |  |  |  |
|  |  | [ c_amr_ns_priority_yes_frenc h]  Show the field ONLY if: [c_amr_ns_priority_french] = '1' and [interview_level_frenc  h] = '1' | 3.3.1 Comment? *Do not need to type the answer - audio recording will capture. | text | | | | |
|  | 172 | [ c_amr_ns_priority_no_dn_fre nch]  Show the field ONLY if: [c_amr_ns_priority_french] = 'O' or [c_amr _ns_priority_frenc h] = '99' | 3.3.1 S'agit-il de criteres a prendre en compte? *Do not  need to type the answer - audio recording will capture. | text | | | | |

I

|  | 173 | [visual_aid_1_french] | Section Header: *Section D: Impact du vaccin anti Shigella Informations 6*  */'endroit des enqueteurs uniquement: Veuillez-vous referer ou support visuel n'I (page suivonte) pour lo section preambule. Shigello est une bocterie pothogene qui peut provoquer des diarrhees severes, y compris des diarrhees sang/antes ou "dysenterie", et constitue une cause majeure de morbidite et de mortolite chez /es enfonts. Le Jardeau mondial de lo diarrhee causee par Shigella chez /es enfants de mains de cinq ans a ete*  *estimee a 75 millions de cos et 64 000 deces par an,* ce *qui en Jait*  *potentiellement la deuxieme cause de mortalite par diarrhee[1]. Des voccins contre Shigello sont actuellement en cours de developpement et pourraient etre disponib/es 6 /'horizon 2025-2030. Un hypothetique voccin contre Shigello pourrait etre odministre sous Jorme injectable et necessiter* ***une au deux doses /ors de la premiere serie, administrees au milieu ou 0***  *la Jin de la premiere onnee de vie. Le cout du vaccin pourrait se situer a*  *environ d'1 dollar US/dose et qu'i/ serait initio/ement soutenu par Gavi. Supposons que votre pays envisage /'introduction de plusieurs vaccins a*  */'horizon 2025-2030, dote a laquelle un vaccin contre Shigella sera*  *probablement disponible. [1] Khalil, The Lancet, 2018.* | descriptive | | | |
| --- | --- | --- | --- | --- | --- | --- | --- |
|  | 174 | [ d_immun_sched_french] | 4.1 Pensez-vous que le [interview_location_french] a encore la possibilite de considerer l'ajout d'un vaccin anti-Shigella dans son calendrier vaccinal de routine? | radio, Required  n | | | |
|  | 175 | [ d_immun_sched_yes_french] | 4.1.1 Quelles eventuelles reserves avez-vous concernant l'ajout d'un nouveau vaccin dans le calendrier vaccinal de routine du [interview_location_french]? *Do not need to type the answer - audio recording will capture. | text | | | |
|  | 176 | [visual_aid_2_bk_french] | Informations a l'endroit des enqueteurs uniquement: | descriptive | | | |
|  |  | Show the field ONLY if: [interview_location_french] = | Veuillez-vous referer au support visuel n°2 (page suivante) pour la question 4.2 |  | | | |
|  |  | '1' |  |  | | | |
|  | 177 | [ d_bk_vaccine_priority_french | 4.2 Au Burkina Faso, on estime que Shigella est responsable chaque annee de 95 400 cas de diarrhee  moderee a severe et de 530 deces chez les enfants de  mains de cinq ans. On estime qu'un vaccin anti Shigella serait efficace a 60% contre les diarrhees moderees a severes a Shigella et pourrait prevenir 52 075 cas de diarrhee moderee a severe et 289 deces dus a la diarrhee a  Shigella chaque annee au Burkina Faso. Compte tenu de ce  fardeau et de l'impact du vaccin et sachant que ce ne sera pas le seul vaccin envisage par votre pays a l'horizon 2025- 2030, quelle priorite accorderiez-vous a !'introduction d'un  vaccin anti Shigella au Burkina Faso? | radio, Required | | | |
|  |  | l |  | 1 | | Priorite absolue |  |
|  |  | Show the field ONLY if: [interview_location_french] = |  |  |  |  |  |
|  |  |  |  | 2 | | Priorite moyenne |  |
|  |  |  |  | 3 | | Priorite faible |  |
|  |  | 1 |  |  |  |  |  |
|  |  |  |  | 4  - | | Pas une priorite |  |
|  |  |  |  |  |  | | |
|  | 178 | [ d_vaccine_priority_why_bk_fr | 4.2.1 Pourquoi avez-vous choisi | text | | | |
|  |  | ench] | [d_bk_vaccine_priority_french]'?*Do not need to type the |  | | | |
|  |  | Show the field ONLY if: | answer - audio recording will capture. |  | | | |
|  |  | [interview_location_french] = |  |  | | | |
|  |  | 1 |  |  | | | |
|  | 179 | [ shigella_priority_slow_amr_fr ench] | 4.3 S'il etait disponible, quelle priorite accorderiez-vous a  un vaccin contre les Shigella pour les jeunes enfants qui aurait la capacite de ralentir le rythme ou de prevenir la resistance aux antibiotiques des Shigella au [interview_location_french]? | radio, Required | | | |
|  |  |  |  | 1 | | Priorite absolue |  |
|  |  |  |  | 2 | | Priorite moyenne |  |
|  |  |  |  | 3 | | Priorite faible |  |
|  |  |  |  | 4 | | Pas une priorite |  |
|  | 180 |  |  |  | | | |
|  |  | [ shigella_prioirity_amr_why_fr ench] | 4.3.1 Pourquoi avez-vous choisi '[shigella_priority_slow_amr_french]'? *Do not need to type the answer - audio recording will capture. | text | | | |
|  | 181 | [visual_aid_3_bk_french] | Informations a l'endroit des enqueteurs uniquement: | descriptive | | | |
|  |  | Show the field ONLY if: [interview_location_french] = | Veuillez utiliser les supports visuels n°3 et n°4 (deux pages suivantes) pour le preambule de la question 4.4. |  | | | |
|  |  | '1' |  |  | | | |
| I 182 | | [visual_aid_4_french] |  | descriptive | | | |

I

|  | 183 | [ d_description_3_french] | II est de plus en plus evident que Shigella a des consequences nefastes specifiques sur la sante et la vie des individus, au-dela de l'episode de diarrhee aigue. La diarrhee causee par la Shigella au cours de la petite enfance peut entraver !'absorption des nutriments dans l'intestin, ce qui entraine la malnutrition. La Shigella a ete associee a un retard de croissance chez les enfants. C'est egalement un facteur de risque connu pour d'autres deces dus a des maladies infectieuses chez les enfants et ii a ete demontre que les enfants souffrant d'un retard de croissance presentent un risque plus eleve de maladies chroniques a l'age adulte. En outre, le retard de croissance a ete associe a une alteration du developpement physique et cognitif et lie a un niveau d'education inferieur et, en fin de compte, a une capacite de gain moindre a l'age adulte. II faut noter qu'un vaccin anti Shigella pourrait egalement contribuer a prevenir certaines de ces autres consequences negatives sur la sante et la vie. Dans cette partie de !'interview, nous voulons comprendre  !'importance que vous accordez aux avantages supplementaires potentiels pour la sante et la vie d'un vaccin anti Shigella, au-dela de la prevention de la diarrhee aigue et des deces associes, lorsque vous envisagez d'introduire un vaccin anti Shigella par rapport a d'autres vaccins que le Burkina Faso pourrait envisager a !'horizon 2025-2030, lorsqu'un vaccin contre Shigella sera probablement disponible. | descriptive | | |
| --- | --- | --- | --- | --- | --- | --- |
|  | 184 | [ d_bk_priority_multi_french]  Show the field ONLY if: [interview_location_french] =  1 | 4.4 Au Burkina Faso, Shigella est responsable d'environ 15 400 cas de retard de croissance modere au grave chaque annee chez les enfants de mains de cinq ans. Precedemment, vous avez considere le vaccin anti Shigella comme une priorite [d_bk_vaccine_priority_french] au regard du fardeau de la Shigella et l'impact que pourrait avoir un vaccin contre la maladie. Maintenant, considerez qu'en plus de l'impact sur les diarrhees moderees a severes a Shigella et sur les deces, un vaccin contre Shigella pourrait prevenir 8 400 cas de retard de croissance chez les enfants de mains de cinq ans chaque annee au Burkina Faso. Avec cet avantage supplementaire, quelle priorite accorderiez-vous a !'introduction de ce vaccin anti Shigella au Burkina Faso, sachant qu'il ne sera pas le seul vaccin envisage par votre pays a !'horizon 2025-2030? | radio, Required | | |
|  |  |  |  | 1 | Priorite absolue |  |
|  |  |  |  | 2 | Priorite moyenne |  |
|  |  |  |  | 3 | Priorite faible |  |
|  |  |  |  | 4  - | Pas une priorite |  |
|  |  |  |  |  | | |
| 1185 | | [ d_priority_multi_why_french  l | 4.4.1 Pourquoi ?*Do not need to type the answer - audio recording will capture. | text | | |
|  | 186 | [ d_bk_priority_wage_french]  Show the field ONLY if: [interview_location_french] = 1 | 4.5 Precedemment, vous avez considere le vaccin anti- Shigella comme une priorite [d_bk_priority_multi_french] au regard de son impact sur la prevention des diarrhees legeres a severes, des deces causes par la shigella et sur le retard de croissance des enfants. Admettons maintenant qu'en plus de ce qui precede, le vaccin pourrait avoir un impact positif sur les revenus des adultes. Avec cet avantage supplementaire, quelle priorite accorderiez-vous a !'introduction de ce vaccin contre la Shigella au [interview_location_french], sachant qu'il ne sera pas le seul vaccin envisage par votre pays a !'horizon 2025-2030? | radio, Required | | |
|  |  |  |  | 1 | Priorite absolue |  |
|  |  |  |  | 2 | Priorite moyenne |  |
|  |  |  |  | 3 | Priorite faible |  |
|  |  |  |  | 4 | Pas une priorite |  |
|  |  |  |  |  | | |
| 1187 | | [why_wage_earning_priority_f rench] | 4.5.1 Pourquoi ?*Do not need to type the answer - audio recording will capture. | text, Required | | |
|  | 188 | [ d_description_4_french]  Show the field ONLY if: [interview_level_french] = '1' | Shigella n'est qu'une des causes de diarrhee et si l'on considere uniquement l'impact du vaccin sur la prevention de la diarrhee, ii peut etre mains rentable comme moyen de lutte contre la diarrhee par rapport au vaccin contre le rotavirus et a d'autres interventions nutritionnelles telles que la supplementation en vitamine A. Toutefois, si l'on tient compte des avantages supplementaires a long terme d'un eventuel vaccin anti Shigella en termes de sante, de developpement et d'economie, le rapport cout-efficacite global du vaccin devient important et, dans certains cas, le vaccin pourrait etre rentable. | descriptive | | |

I

I

| 189 | [ d_vaccine_benefits_french]  Show the field ONLY if: [interview_level_french] = '1' | 4.6 Dans quelle mesure ces avantages supplementaires d'un vaccin anti Shigella et leur potentiel d'amelioration de la rentabilite ou des gains economiques realisables grace au vaccin vous rendent-ils plus susceptibles de donner la priorite au vaccin? | radio, Required |
| --- | --- | --- | --- |
| 190 | [ d_vaccine_benefits_why_fren ch]  Show the field ONLY if: [interview_level_french] = '1' | 4.6.1 Pourquoi? *Do not need to type the answer - audio recording will capture. | text |
| 191 | [ d_specific_price_french]  Show the field ONLY if: [interview_level_french] = '1' | 4.7 Existe-t-il un plafond specifique en termes de prix, comme le coCit par dose ou par enfant entierement vaccine, au-dela duquel vous ne seriez pas favorable a l'ajout d'un vaccin contre Shigella au calendrier de vaccination systematique? Si oui, quel est ce prix? Quels facteurs prenez-vous en consideration lorsque vous prenez une telle decision? *Do not need to type the answer - audio recording will capture. | text |
| 192 | [visual_aid_S_bk_french]  Show the field ONLY if: [interview_location_french] = 1 | Section Header: *Section E: Caracteristiques des vaccins}e veux que vous imaginiez que le Burkina Faso envisage d'introduire un vaccin contre la Shigella. Pour rappel, le vaccin devrait necessiter 1 ou 2 doses pour la premiere serie, administrees entre le milieu et la fin de la premiere annee* ***de vie. Pour ces prochaines questions, je voudrais m'interesser b votre***  *preference pour des attributs specifiques du vaccin, avec un accent sur le calendrier vaccinal, /es vaccins a antigene unique ou combines, et la voie d'administration.*  A l'endroit des enqueteurs uniquement: Veuillez-vous referer au support visuel n°5 (page suivante) pour la question 5.1. | descriptive |
| 193 | [ e_bk_admin_time_french]  Show the field ONLY if: [interview_location_french] = 1 | 5.1 Je voudrais commencer par vous interroger sur le calendrier vaccinal. Admettons que le vaccin anti Shigella est un vaccin a antigene unique administre par injection, qui necessite UNE DOSE pour la premiere serie soit a 6 MOIS, ce qui necessite un nouveau contact de vaccination, soit a 9 MOIS lorsque le vaccin contre la rougeole et la rubeole et le vaccin contre la fievre jaune sont deja administres. De maniere operationnelle, quelle quel contact vaccinal preferez-vous? | radio, Required |
| 194 | [ e_bk_admin_time_why_frenc  h]  Show the field ONLY if: [e_bk_admin_time_french]=1 or [e_bk_admin_time_french]  =2 or [e_bk_admin_time_frenc h]=3 and [interview_location_f rench] = 1 | 5.1.1 Pourquoi avez-vous choisi '[e_bk_admin_time_french]'? *Do not need to type the answer - audio recording will capture. | text |
| 195 | [ e_admin_time_6mo_french]  Show the field ONLY if: [e_bk_admin_time_french] = '2' or [e_ghana_admin_time_fr ench] = '2' or [e_kenya_admin  _time_french] = '2' or [e_nepal  _admin_time_french] = '2' or [e_vietnam_admin_time_frenc h] = '2' | 5.2 Je veux maintenant que vous imaginiez que le vaccin injectable a antigene unique ne peut etre propose qu'a un noveau contact vaccinal. Comment cela affecterait-il votre volonte d'envisager !'introduction d'un vaccin anti Shigella dans le calendrier de vaccination de routine? | radio, Required |
| 196 | [ e_admin_time_9mo_french]  Show the field ONLY if: [e_bk_admin_time_french] = '1' or [e_ghana_admin_time_fr ench] = '1' or [e_kenya_admin  _time_french] = '1' or [e_nepal  _admin_time_french] = '1' or [e_vietnam_admin_time_frenc h] = '1' | 5.2 Je veux maintenant que vous imaginiez que le vaccin injectable a antigene unique ne peut etre propose qu'a un contact vaccinal deja existant. Comment cela affecterait-il votre volonte d'envisager !'introduction d'un vaccin anti Shigella dans le calendrier de vaccination de routine? | radio, Required |

| 2 | Beaucoup plus susceptible |
| --- | --- |
| 1 | Moderement plus susceptible |
| 0  - | N'affecterait pas ma decision |

| 1 | un nouveau contact vaccinal |
| --- | --- |
| 2 | un contact vaccinal deja existant |
| 3 | l'un ou l'autre |

| 1 | Cela n'affecterait pas ma decision |
| --- | --- |
| 2 | Je serai moderement mains en faveur de  !'introduction de ce vaccin |
| 3 | Je serais beaucoup mains en faveur de  !'introduction de ce vaccin |
| 4 | Je ne serais pas en faveur de !'introduction de ce vaccin |

| 1 | Cela n'affecterait pas ma decision |
| --- | --- |
| 2 | Je serai moderement mains en faveur de  !'introduction de ce vaccin |
| 3 | Je serais beaucoup mains en faveur de  !'introduction de ce vaccin |
| 4 | Je ne serais pas en faveur de l'introduction de ce vaccin |

| 197 | [ e_admin_time_notpref_why_ french]  Show the field ONLY if: [e_admin_time_6mo_french]  = '1' or [e_admin_time_6mo_fr ench] = '2' or [e_admin_time_ 6mo_french] = '3' or [e_admin  _time_6mo_french] = '4' | 5.2.2 Pourquoi avez-vous choisi '[e_admin_time_6mo_french]'?*Do not need to type the answer - audio recording will capture. | text |
| --- | --- | --- | --- |
| 198 | [ e_admin_time_notpref_why2  _french]  Show the field ONLY if: [e_admin_time_9mo_french]  = '1' OR [e_admin_time_9mo_f rench] = '2' OR [e_admin_time  _9mo_french] = '3' OR [e_adm in_time_9mo_french] = '4' | 5.2.2 Pourquoi avez-vous choisi '[e_admin_time_9mo_french]'?*Do not need to type the answer - audio recording will capture. | text |
| 199 | [ e_two_doses_french] | 5.3 Supposons maintenant que le vaccin antigenique unique injectable contre Shigella necessite DEUX DOSES  lors de la premiere serie. Comment cette exigence d'une premiere serie a DEUX DOSES affecterait-elle votre volonte  d'envisager !'introduction d'un vaccin anti Shigella dans le calendrier de vaccination de routine? | radio, Required |
| 200 | [ e_two_doses_why_french] | 5.3.1 Pourquoi avez-vous choisi '[e_two_doses_french]'?  *Do not need to type the answer - audio recording will capture. | text |
| 201 | [visual_aid_6_bk_french]  Show the field ONLY if: [interview_location_french] = 1 | A l'endroit des enqueteurs uniquement: Veuillez-vous referer au support visuel n°6 (page suivante) pour la question 5.4. | descriptive |
| 202 | [ e_single_dose_inject_french] | 5.4 Supposons que le Burkina Faso ait decide d'inclure dans le calendrier vaccinal de routine un vaccin injectable a dose unique lors de la premiere serie contre Shigella a l'age  de 9 mois. Le vaccin est disponible soit sous la forme  d'antigene unique, soit dans le cadre d'un vaccin combine, dans lequel Shigella est associe a un autre antigene en une  seule injection. Sous quelle forme souhaiterez-vous avoir le vaccin? | radio, Required |
| 203 | [ e_single_dose_inject_why_fre nch] | 5.4.1 Pourquoi avez-vous choisi '[e_single_dose_inject_french]'? *Do not need to type the answer - audio recording will capture. | text |

| 1 | Cela n'affecterait pas ma decision |
| --- | --- |
| 2 | Je serai moderement mains en faveur de  !'introduction de ce vaccin |
| 3 | Je serais beaucoup mains en faveur de  !'introduction de ce vaccin |
| 4  - | Je ne serais pas en faveur de !'introduction de ce vaccin |

| 1 | Un vaccin a antigene unique (Shigella  uniquement) |
| --- | --- |
| 2 | Un vaccin combine (Shigella plus un autre antigene) |
| 0 | Aucune preference entre les vaccins uniques et combines |

t

|  | 204 | [ stand_alone_cascade_french  l  Show the field ONLY if: [e_single_dose_inject_french]  = 1 | 5.5 Lequel de ces attributs pourrait changer votre intention de considerer !'introduction d'un vaccin a antigene unique  (Shigella uniquement) presentation de vaccin anti-Shigella. Selectionnez toutes les reponses pertinentes. | checkbox, Required | | | |
| --- | --- | --- | --- | --- | --- | --- | --- |
|  |  |  |  |  | | stand_alone_cascade_french | Le vaccin a  antigene unique (Shigella uniquement) est  moderement |
|  |  |  |  |  |  |  | mains efficace |
|  |  |  |  |  |  |  | dans la |
|  |  |  |  |  |  |  | prevention de la |
|  |  |  |  |  |  |  | diarrhee  moderee a |
|  |  |  |  |  |  |  | severe et/au les |
|  |  |  |  |  |  |  | deces |
|  |  |  |  |  |  |  | comparativemen |
|  |  |  |  |  |  |  | au vaccin |
|  |  |  |  |  |  |  | combine |
|  |  |  |  | 2 | | stand_alone_cascade_french_2 | Le vaccin a |
|  |  |  |  |  | |  | antigene unique |
|  |  |  |  |  | |  | (Shigella |
|  |  |  |  |  | |  | uniquement) |
|  |  |  |  |  | |  | demande |
|  |  |  |  |  | |  | moderement un |
|  |  |  |  |  | |  | peu plus de |
|  |  |  |  |  | |  | capacite de |
|  |  |  |  |  | |  | stockage a 2-8°C |
|  |  |  |  |  | |  | comparativemen |
|  |  |  |  |  | |  | au vaccin |
|  |  |  |  |  | |  | combine |
|  |  |  |  |  | 3 | stand_alone_cascade_french- 3 | Le vaccin a  antigene unique |
|  |  |  |  |  |  |  | (Shigella |
|  |  |  |  |  |  |  | uniquement) est |
|  |  |  |  |  |  |  | moderement un |
|  |  |  |  |  |  |  | peu plus cher |
|  |  |  |  |  |  |  | comparativemen |
|  |  |  |  |  |  |  | au vaccin |
|  |  |  |  |  |  |  | combine |
|  |  |  |  |  | stand_alone_cascade_french_4 | | Aucune de ces |
|  |  |  |  |  |  | | reponses |

t

t

| 1 | Cela n'affecterait pas ma decision |
| --- | --- |
| 2 | Je serai moderement mains en faveur de  !'introduction de ce vaccin |
| 3 | Je serais beaucoup mains en faveur de  !'introduction de ce vaccin |
| 4  - | Je ne serais pas en faveur de !'introduction de ce vaccin |

|  | 205 | [ combo_cascade_french]  Show the field ONLY if: [e_single_dose_inject_french]  =2 | 5.5 Lequel de ces attributs pourrait changer votre intention de considerer !'introduction d'un vaccin combine presentation de vaccin anti-Shigella. Selectionnez toutes les reponses pertinentes. | checkbox, Required | | | |
| --- | --- | --- | --- | --- | --- | --- | --- |
|  |  |  |  |  | | combo_cascade_french | Le vaccin combine (Shigella plus un autre antigene) est moderement mains efficace dans la prevention  de la diarrhee moderee a severe  eUou les deces  comparativement au vaccin a  antigene unique (Shigella uniquement). |
|  |  |  |  | 2 | | combo_cascade_french- 2 | Le vaccin combine (Shigella plus un autre antigene) demande moderement un peu plus de capacite de stockage a 2-8°(  comparativement au vaccin a  antigene unique (Shigella uniquement). |
|  |  |  |  |  | 3 | combo_cascade_french- 3 | Le vaccin combine (Shigella plus un autre antigene) est moderement un peu plus cher  comparativement au vaccin a  antigene unique (Shigella  uniquement). |
|  |  |  |  |  | combo_cascade_french_4 | | Aucune de ces reponses |
|  | 206 | [ stand_alone_cascade_why_fr ench]  Show the field ONLY if: [stand_alone_cascade_french (1)] = '1' or [stand_alone_casc ade_french(2)] = '1' or [stand_ alone_cascade_french(3)] = '1' or [stand_alone_cascade_fren ch(4)] = '1' or [combo_cascade  _french(1 )] = '1' or [combo_ca scade_french(2)] = '1' or [com bo_cascade_french(3)] = '1' or [combo_cascade_french(4)] = '1' | 5.5.1 Pourquoi? *Do not need to type the answer - audio recording will capture. | text | | | |
|  | 207 | [ e_combo_pref_french]  Show the field ONLY if: [e_single_dose_inject_french]  = '2' | 5.6 lmaginez maintenant que le vaccin contre Shigella ne soit propose que sous la forme de a antigene unique  (Shigella uniquement). Comment cela affecterait-il votre volonte d'envisager !'introduction d'un vaccin contre Shigella dans le calendrier de vaccination de routine? | radio, Required | | | |

| 1 | Cela n'affecterait pas ma decision |
| --- | --- |
| 2 | Je serai moderement mains en faveur de  !'introduction de ce vaccin |
| 3 | Je serais beaucoup mains en faveur de  !'introduction de ce vaccin |
| 4  - | Je ne serais pas en faveur de !'introduction de ce vaccin |

| 1 | Vaccin oral |
| --- | --- |
| 2 | Vaccin injectable |
| 3 | Pas de preference entre les vaccins oraux et injectables |

| 208 | [ e_combo_pref_2_french]  Show the field ONLY if: [e_single_dose_inject_french]  = '1' | 5.6 lmaginez maintenant que le vaccin contre Shigella ne soit propose que sous la forme devaccin combine (Shigella plus un autre antigene). Comment cela affecterait-il votre volonte d'envisager !'introduction d'un vaccin contre Shigella dans le calendrier de vaccination de routine? | radio, Required | | | |
| --- | --- | --- | --- | --- | --- | --- |
| 209 | [ e_combo_pref_why_french]  Show the field ONLY if: [e_combo_pref_french] = '1' o r [e_combo_pref_french] = '2' or [e_combo_pref_french] = '3' or [e_combo_pref_french]  = '4' | 5.6.1 Pourquoi avez-vous choisi '[e_combo_pref_french]'?  *Do not need to type the answer - audio recording will capture. | text | | | |
| 210 | [ e_combo_pref_2_why_french  l  Show the field ONLY if: [e_combo_pref_2_french] = '1' or [e_combo_pref_2_french] = '2' or [e_combo_pref_2_frenc h] = '3' or [e_combo_pref_2_fr ench] = '4' | 5.6.1 Pourquoi avez-vous choisi '[e_combo_pref_2_french]'?  *Do not need to type the answer - audio recording will capture. | text | | | |
| 211 | [visual_aid_7_bk_french]  Show the field ONLY if: [interview_location_french] = 1 | A l'endroit des enqueteurs uniquement: Veuillez-vous referer au support visuel n°7 (page suivante) pour la question 5.7. | descriptive | | | |
| 212 | [ e_route_admin_only_french] | 5.7 Supposons que le Burkina Faso ait decide de fournir  une dose unique du vaccin anti-shigella lors de la premiere serie a l'age 9 mois. Le vaccin peut etre administre par voie  orale au parenterale par injection. Quelle voie d'administration prefereriez-vous? | radio, Required | | | |
| 213 | [ e_route_admin_only_why_fre nch]  Show the field ONLY if: [e_route_admin_only_french]  = '1' or [e_route_admin_only_f rench] = '2' or [e_route_admin  _only_french] = '3' | 5.7.1 Pourquoi avez-vous choisi '[e_route_admin_only_french]'?*Do not need to type the answer - audio recording will capture. | text | | | |
| 214 | [ e_oral_cascade_french J  Show the field ONLY if: [e_route_admin_only_french]  =1 | 5.8 Lequel des attributs ci-dessous pourrait changer votre intention de considerer !'introduction d'un vaccin anti­ Shigella oral. Selectionnez toutes les reponses pertinentes. | checkbox, Required | | | |
|  |  |  |  | | e_oral_cascade_french | Le vaccin oral est moderement mains efficace dans la prevention des diarrhees et/au des deces comparativement au vaccin injectable |
|  |  |  | 2 | | e_oral_cascade_french- 2 | Le vaccin oral demande moderement un peu plus d'espace de stockage a 2-8°C comparativement au vaccin injectable |
|  |  |  |  | 3 | e_oral_cascade_french- 3 | Le vaccin oral est moderement plus cher comparativement  au vaccin injectable |
|  |  |  |  | e_oral_cascade_french_4 | | Aucune de ces reponses |

|  | 215 | [ e_injectable_cascade_french  l  Show the field ONLY if: [e_route_admin_only_french]  =2 | 5.8 Lequel des attributs ci-dessous pourrait changer votre intention de considerer !'introduction d'un vaccin anti­ Shigella injectable. Selectionnez toutes les reponses pertinentes. | checkbox, Required | | |
| --- | --- | --- | --- | --- | --- | --- |
|  |  |  |  | 1 | e_injectable_cascade_french_1 | Le vaccin injectable est moderement mains efficace dans la prevention des diarrhees et/ou des deces comparativemen au vaccin oral |
|  |  |  |  | 2 | e_injectable_cascade_french_2 | Le vaccin injectable demande moderement un peu plus d'espace de stockage a 2-  3oc  comparativemen au vaccin oral |
|  |  |  |  | 3 | e_injectable_cascade_french_3 | Le vaccin injectable est moderement plus cher comparativemen  au vaccin oral |
|  |  |  |  | 4 e_injectable_cascade_french_4 | | Aucune de ces reponses |
|  | 216 | [ e_oral_injectable_cascade_w hy_french]  Show the field ONLY if: [e_oral_cascade_french(1)] = '1' or [e_oral_cascade_french  (2)] = '1' or [e_oral_cascade_fr ench(3)] = '1' or [e_oral_casca de_french(4)] = '1' or [e_inject able_cascade_french(1)] = '1' or [e_injectable_cascade_fren ch(2)] = '1' or [e_injectable_ca scade_french(3)] = '1' or [e_inj ectable_cascade_french(4)] = '1' | 5.8.1 Pourquoi? *Do not need to type the answer- audio recording will capture. | text | | |
|  | 217 | [ e_oral_only_french]  Show the field ONLY if: [e_route_admin_only_french]  =2 | 5.9 lmaginez maintenant que le vaccin contre Shigella ne soit propose que sous la forme de oral. Comment cela affecterait-il votre volonte d'envisager l'introduction d'un vaccin contre Shigella dans le calendrier de vaccination de routine? | radio, Required | | |
|  | 218 | [ e_injectable_only_french]  Show the field ONLY if: [e_route_admin_only_french]  = 1 | 5.9 lmaginez maintenant que le vaccin contre Shigella ne soit propose que sous la forme de injectable. Comment cela affecterait-il votre volonte d'envisager !'introduction d'un vaccin contre Shigella dans le calendrier de vaccination de routine? | radio, Required | | |
|  | 219 | [ e_route_admin_only_2_why_f rench]  Show the field ONLY if: [e_oral_only_french] = '1' or [e  _oral_only_french] = '2' or [e_ oral_only_french] = '3' or [e_or al_only_french] = '4' | 5.9.1 Pourquoi avez-vous choisi '[e_oral_only_french]'? *Do not need to type the answer - audio recording will capture. | text | | |

t

t

t

| 1 | N'affecterait pas ma decision |
| --- | --- |
| 2 | Moderement mains disposes a envisager ce vaccin |
| 3 | Beaucoup mains disposes a envisager ce vaccin |
| 4  - | Je ne considererais pas ce vaccin |

| 1 | N'affecterait pas ma decision |
| --- | --- |
| 2 | Moderement mains disposes a envisager ce vaccin |
| 3 | Beaucoup mains disposes a envisager ce vaccin |
| 4 | Je ne considererais pas ce vaccin |

| 1 | N'affecterait pas ma decision |
| --- | --- |
| 2 | Moderement mains disposes a envisager ce vaccin |
| 3 | Beaucoup mains disposes a envisager ce vaccin |
| 4  - | Je ne considererais pas ce vaccin |

| 1 | N'affecterait pas ma decision |
| --- | --- |
| 2 | Moderement mains disposes a envisager ce vaccin |
| 3 | Beaucoup mains disposes a envisager ce vaccin |
| 4 | Je ne considererais pas ce vaccin |

| 1 | N'affecterait pas ma decision |
| --- | --- |
| 2 | Moderement mains disposes a envisager ce vaccin |
| 3 | Beaucoup mains disposes a envisager ce vaccin |
| 4 | Je ne considererais pas ce vaccin |

| 1 | N'affecterait pas ma decision |
| --- | --- |
| 2 | Moderement mains disposes a envisager ce vaccin |
| 3 | Beaucoup mains disposes a envisager ce vaccin |
| 4 | Je ne considererais pas ce vaccin |

| 220 | [ e_route_admin_only_2_why_ 2_french l  Show the field ONLY if: [e_injectable_only_french] = '1' or [e_injectable_only_frenc h] = '2' or [e_injectable_only_f rench] = '3' or [e_injectable_o nly_french] = '4' | 5.9.1 Pourquoi avez-vous choisi '[e_injectable_only_french]'? *Do not need to type the answer - audio recording will capture. | text |
| --- | --- | --- | --- |
| 221 | [ e_description_2_french] | II existe plusieurs autres attributs d'un hypothetique vaccin contre Shigella qui pourraient avoir des implications operationnelles pour son administration. lmaginez que le Burkina Faso envisage d'introduire un vaccin hypothetique contre Shigella qui est un vaccin injectable a antigene  unique, avec une serie primaire administree en une seule dose, a l'age de 6 ou 9 mois. | descriptive |
| 222 | [ e_lyophilized_french] | 5.1O Le vaccin est lyophilise et necessite une reconstitution avant utilisation. Comment cela affecterait-il votre volonte d'envisager !'introduction d'un vaccin contre Shigella dans le calendrier de vaccination de routine? | radio, Required |
| 223 | [ e_lyophilized_why_french] | 5.10.1 Pourquoi avez-vous choisi '[e_lyophilized_french]'?  *Do not need to type the answer - audio recording will capture. | text |
| 224 | [ e_packaged_single_dose_fre nch] | 5.11 Le vaccin est disponible en dose unique conditionne (c'est-a-dire en seringues preremplies). Comment cela affecterait-il votre volonte d'envisager !'introduction d'un vaccin contre Shigella dans le calendrier de vaccination routine? | radio, Required |
| 225 | [ e_packaged_single_dose_wh y_french] | 5.11.1 Pourquoi avez-vous choisi '[e_packaged_single_dose_french]'? *Do not need to type the answer - audio recording will capture. | text |
| 226 | [ e_booster_year_2_french] | 5.12 Le vaccin peut necessiter une dose de rappel administree au cours de la deuxieme annee de vie. Quel effet cela aurait-ii sur votre volonte d'envisager  !'introduction d'un vaccin contre Shigella dans le calendrier de vaccination de routine? | radio, Required |
| 227 | [ e_booster _year_2_why_frenc  h] | 5.12.1 Pourquoi *avez-vous* choisi  '[e_booster _year_2_french]'7 *Do not need to type the answer - audio recording will capture. | text |
| 228 | [ e_storage_neg_20_french] | 5.13 Le vaccin doit etre conserve a une temperature inferieure ou egale a -20°C. Comment cela affecterait-il votre volonte d'envisager !'introduction d'un vaccin anti Shigella dans le cadre du calendrier de vaccination de routine? | radio, Required |
| 229 | [ e_storage_neg_20_why_frenc  h] | 5.13.1 Pourquoi avez-vous choisi '[e_storage_neg_20_french]'? *Do not need to type the answer - audio recording will capture. | text |
| 230 | [ e_other_attributes_french] | 5.14 Y a-t-il d'autres attributs qui pourraient affecter votre volonte d'envisager un vaccin contre Shigella dans le cadre du calendrier de vaccination de routine? Quels attributs? Pourquoi? *Do not need to type the answer - audio recording will capture. | text |

231

1232

I

233

234

235

236

[f_sub_nat_intro_french]

[why_do_not_need_to_type_t h_french]

[ f_sub_nat_acceptability _fren ch]

[f_sub_nat_feasibility_french]

[g_other_comments_french]

[ interview_guide_french_com plete J

Section Header: *Section F: Preferences sous notionole en matiere* ***d'introduction de nouveaux vaccins C'est la dernif!re partie de notre entretien. Pour /es trois dernif!res questions de notre entretien, supposons***

*que le Burkina Faso presente une incidence de Shigella tres difterente selon /es zones geographiques ou qu'il soit confronte a des epidemies*

*annuelles de Shigella dons certaines regions. Je voudrais maintenant vous interroger sur la possibilite d'introduire un vaccin contre Shigella a l'echel/e sous nationale, par exemple dons /es zones a forte incidence*

*uniquement, comme approche de /utter contre /es epidemies.*

- 1. Etes-vous en faveur de !'introduction d'un vaccin anti Shigella a l'echelle sous nationale?
     1. Pourquoi ?*Do not need to type the answer - audio recording will capture.
  2. Selan vous, quels peuvent etre les problemes d'acceptabilite que vous entrevoyez par rapport a la mise en ceuvre d'un programme de vaccination contre Shigella a l'echelle sous nationale? *Do not need to type the answer - audio recording will capture.

*Considerez des difticultes au niveau des groupes suivants: /es decideurs*

*politiques, /es agents de sante, /es membres de la communaute ?*

- 1. Selan vous, quels peuvent etre les problemes de faisabilite operationnelle au de fourniture de service que vous entrevoyez par rapport a la mise en ceuvre d'un programme de vaccination contre la Shigella a l'echelle sous nationale? *Do not need to type the answer - audio recording will capture.

*Considerez des difticultes au niveau des groupes suivants: !es decideurs*

*politiques, /es agents de sante, /es membres de la communaute ?*

Section Header: *Section G: Fin de l'entretien*

7.1 Outre les sujets deja abordes dans cet entretien, y a-t-il autre chose que vous aimeriez ajouter et qui aurait un impact sur votre regard sur !'introduction d'un vaccin contre Shigella dans le calendrier vaccinal de routine dans votre contexte? *Do not need to type the answer - audio recording will capture.

*Terminez l'entretien; remerciez /es repondants de leur participation*

Section Header: *Form Status*

Complete?

radio, Required

n

text

text

text

text

dropdown

| 0 | Incomplete |
| --- | --- |
| 1 | Unverified |
| 2  - | Complete |

Ins-trument: **INTERVIEW GUIDE - VIETNAMESE** (interview_guide_vietnamese)

237 [ unique_id_vn J X.0 Ma nghien ci'.lu: text, Required

- -- ---

238 [ recording_confirmation_vnJ X.1 Anh/chi c6 dong y cha chung toi ghi am cuoc ph6ng van radio, Required

nay khong? 1 C6

0 Khong

239 [ recording_start_script_vn J X.2 Phan gi6"i thieu bat dau ghi am: descriptive

"Bat dau CUQC ph6ng van v6"i ngu'oi cung cap thong tin [unique_id_vn]"

1240 [ interview_date_vn J X.3 Ngay ph6ng van: text (date_dmy), Required

I Field Annotation: @TODAY

241 [ interview_location_vn J X.4 Dia diem ph6ng van radio, Required

1. Burkina Faso
2. Ghana
3. Kenya
4. Nepal
5. Viet Nam

-

I I

| 242 [ city_town_district_vnJ X4.1 Tinh/Thanh ph6 - Quan/ Huyen/ Thi xa text, Required | | | |
| --- | --- | --- | --- |
| 243 | [ interview_level_vn J | X.5 Tuyen ph6ng van | radio, Required |

1. Tuyen Trung u'ang
2. Tuyen *co* s6" (Tiep t1,1c phfa du'6"i)

244

245

246

247

248

249

250

251

252

253

254

255

256

257

[ hcf_level_vn J

Show the field ONLY if: [interview_level_vn] = '2'

[ hcf_publicvprivate_vn J

Show the field ONLY if: [interview_level_vn] = '2'

[ hcf_name_vn J

Show the field ONLY if: [interview_level_vn] = '2'

[a_org_vn J

[a_title_vn J [a_years_experience_vn J [a_nitag_icc_vn J

Show the field ONLY if: [interview_level_vn] = '1' or [h cf_level_vn] = '3'

[ b_u5_health_concerns_vn]

[ b_u5_health_concern_vn]

[ b_u5_health_concern_why_v n]

[ b_interv_priority_vn J

[ b_importance_growth_vn J

[b_importance_growth_why_v n]

[ b_heard_of_shigella_vn]

- - 1. Cd sci cua anh chi thuc;ic tuyen nao?
    2. Cd sci nay la cua nha nl16'c hay tlf nhan?
    3. Ten Cd *sd* y te

Section Header: *Phan A: Thong tin chung ctia doi tlll;mg awe phong va·n*

- 1. Anh/chi dang lam viec cho Cd quan/to chuc nao? *Do not need to type the answer - audio recording will capture.
  2. Chuc vu/vi trf cua anh/chi t9i cd quan/to chuc d6? *Do not need to type the answer - audio recording will capture.
  3. Thai gian Anh/chi da c6ng tac *c1* vi trf nay? *Do not need to type the answer - audio recording will capture.
  4. Anh/chi c6 dang la thanh vien cua Uy Ban Tu van Quoc gia ve tiem chung (NITAG) ho c Uy ban dieu phoi lien nganh ve tiem chung (ICC) ho c dang thudng xuyen tham gia cac cuc;ic hop cua hai Uy ban nay kh6ng?

Section Header: *Phan B: Uu tien sue khoe tre* em *vii nhqn thuc vi! benh tieu chay*

- 1. Sau day, chung ta se cung trao doi ve van de suc kh6e tre em t9i Viet Nam. Du6'i g6c dc;i chuyen mon cua mlnh, Anh/Chi hay dlfa ra 2-3 van de quan trong hang dau lien quan den suc kh6e va Sl/ phat trien *Cl* tre dl16'i 5 tuoi t9i Viet Nam hien nay? *Do not need to type the answer - audio recording will capture.
  2. Anh/chi danh gia ve muc dc;i nghiem trong cua tieu chay nhlf the nao doi v6'i tre em dl16'i 5 tuoi trong linh Vl/C c6ng tac cua anh/chi?
     1. T<;1i sao? *Do not need to type the answer - audio recording will capture.

*G<;li y: cac trieu chung cop tinh, di chung, tac d(ing lam sang hoqc tac d(ing*

*xii h(ii.*

- 1. Nhil'ng bien phap can thiep dlfc;lc coi la lfu tien hang dau

va Llu tien thll hai cua Anh/Chi de ngan ngua tieu chay? *Do not need to type the answer - audio recording will capture.

*G(li y:voe xin (Rota vi rut, ta), nude sochlve sinh, dinh duang, th6i quen rua toy*

- 1. Suy dinh dlfong the thap c6i c6 mllc dc;i nghiem trong nhlf the nao doi v6'i suc kh6e tre em dl16'i 5 tuoi *Cl* Viet Nam?
     1. T9i sao? *Do not need to type the answer - audio recording will capture.

*G<;1i y: cac trieu chung cop tinh, di chung, tac d(ing lam sang hol)c tac <1¢ng*

*xiih(ii.*

- 1. Anh/chi da tung nghe thong tin gl ve IY. trl,fc trung/Shigella chua?

radio, Required

| 1 | Tuyen tinh |
| --- | --- |
| 2 | Tuyen huyen |
| 3  - | Tuyen xa |

radio, Required

| 1 | Cd Sci Nha nl16'c |
| --- | --- |
| 2 | Cd sci y te tlf nhan |

text, Required, Identifier

text, Identifier

text, Identifier

text, Identifier

radio, Required

|  | C6 |
| --- | --- |
| 2 Khong | |

text

radio, Required

| 1 | Mc;it van de SLIC kh6e rat nghiem trong |
| --- | --- |
| 2 | Mot van de suc kh6e nghiem trong, nhung khong nam trong so cac van de suc kh6e hang dau |
| 3  - | Khong phai la van de nghiem trong so v6'i cac van de suc kh6e khac |

text

text

radio, Required

| 1 | Mc;it van de suc kh6e rat nghiem trong |
| --- | --- |
| 2 | Mc;it van de SLIC kh6e nghiem trong, nhung khong nam trong so cac van de sl/c kh6e hang dau |
| 3 | Khong phai la van de nghiem trong so v6'i cac van  de SL/C kh6e khac |

text

radio, Required

1. C6

0 Khong

|  | 258 | [ shigella_awareness_vn]  Show the field ONLY if: [b_heard_of_shigella_vn] = '1' | 2.6 Anh/chi c6 the chia se cu the nhO'ng thong tin ma  anh/chi biet ve ly tn,rc trung/Shigella? *Do not need to type the answer - audio recording will capture.  *G(li y:g6nh ndnglqui'in* the *nguy Cd coo, nguon lay truyen, chiin do6n, tril}u chung, di ch(lng vd dieu tr/.* | text | | | |
| --- | --- | --- | --- | --- | --- | --- | --- |
|  | 259 | [ b_shigella_importance_vn]  Show the field ONLY if: [b_heard_of_shigella_vn] = '1' | 2.7 Anh/chi danh gia ly tn/c trung/Shigella la van de su'c khoe nghiem trong nhu' the nao d6i vdi tre em du'di 5 tuoi trong ITnh v1,1c cong tac cua anh/chi | radio, Required | | | |
|  | 260 | [ b_shigella_aspects_vn]  Show the field ONLY if: [b_heard_of_shigella_vn] = '1' | 2.8 NhO'ng van de nao cua benh ly trl,/c trung/Shigella khien anh/chi quan tam nhat? *Do not need to type the answer - audio recording will capture.  *G(li y: tam quan trong cua tieu ch6y (cac trieu chung cap tinhJ, suy dinh d11ang thap coi (di chung), va cac tac dong lam sang hodc xii h¢i.* | text | | | |
|  | 261 | [ b_shigella_priority_vn]  Show the field ONLY if: [b_heard_of_shigella_vn] = '1' | 2.9 Neu gia su' da c6 vac xin phong benh ly tn/c trung/Shigella, Anh/chi hay danh gia mu'c di;> u'U tien khi gidi thieu vac-xin phong benh ly tn/c trung/Shigella cha tre em *d* Viet Nam? | radio, Required | | | |
|  |  |  |  | 1 | Mu'c u'u tien cao |  | |
|  |  |  |  | 2 | Mu'c u'u tien trung blnh |  |  |
|  |  |  |  | 3 | Mu'c u'u tien thap |  |  |
|  |  |  |  | 4 | Khong phai u'U tien |  |  |
|  | 262 |  |  |  | | | |
|  |  | [ c_amr_importance_vn] | Section Header: *Phan C: Khang kh6ng sinh* M/jt *trong nhilng ph11ang ph6p dieu tri tieu chay la su' dung kh6ng sinh. C6 the thay tinh trang khang kh6ng sinh vdi nhieu vi khuan gay b/!nh kh6c nhau dang lam gia tang m6i lo ngqi tren toan the gidi.*  3.1 La mot van de y te can quan tam, Anh/Chi hay danh gia tlnh tr<;1ng nghiem trong cua khang khang sinh trong linh vl,/c cong tac cua anh/chi? | radio, Required | | |  |
|  |  |  |  | 3 | Mi;>t van de rat nghiem trong | |  |
|  |  |  |  | 2 | Mot van de nghiem trong, nhu'ng kh6ng n/im trong s6 cac van de su'c khoe hang dau | |  |
|  |  |  |  | 1 | Khong phai la van de nghiem trong so vdi cac van de su'c khoe khac | |  |
|  |  |  |  | 0 | Khong biet | |  |
| 1263 | | [ c_amr_importance_why_vn] | 3.1.1 T<;1i sao? *Do not need to type the answer - audio recording will capture. | text | | | |
|  | 264 | [ c_amr_treatment_costs_vn]  Show the field ONLY if: [interview_level_vn] = '2' | 3.2 Do SI/ gia tang tlnh tr<;1ng khang khang sinh, cac phu'dng phap dieu tri t<;1i cac *Cd* soy te c6 thay doi trong 5 nam qua 6 Viet Nam khong? Neu c6, thay doi nhu' the nao? | radio, Required | | | |
|  | 265 |  |  |  |  |  |  |
|  |  | [ c_amr_treatment_costs_yes_ vn]  Show the field ONLY if: [c_amr_treatment_costs_vn] = '1' | 3.2.1 Nhu' the nao? *Do not need to type the answer - audio recording will capture. | text | | | |
|  | 266 | [ c_amr_ns_priority_vn]  Show the field ONLY if: [interview_level_vn] = '1' | 3.3 Thong tin ve tlnh tr<;1ng khang khang sinh cua mi;>t tac nhan gay bi)nh c6 can du'Q"c xem xet khi u'u tien gidi thii)u lo<;1i vac xin mdi ch6ng l<;1i tac nhan gay benh d6 khong? | radio, Required | | | |
|  | 267 |  |  |  |  |  |  |
|  |  | [ c_amr_ns_priority_yes_vn]  Show the field ONLY if: [c_amr_ns_priority_vn] = '1' an d [interview_level_vn] = '1' | 3.3.1 Nhu' the nao? *Do not need to type the answer - audio recording will capture. | text | | | |
|  | 268 | [ c_amr_ns_priority_no_dn_vn  l  Show the field ONLY if: [c_amr_ns_priority_vn] = 'O' or [c_amr_ns_priority_vn] = '99' | 3.3.1 C6 phai la tieu chf can du'QC xem xet khong? *Do not need to type the answer - audio recording will capture. | text | | | |

| 1 | Mi;>t van de su'c khoe rat quan trong |
| --- | --- |
| 2 | Mi;>t van de su'c khoe quan trong nhu'ng khong phai la van de quan trong nhat |
| 3 | Khong phai van de quan trong so vdi cac van de khac |
| 99  - | Khong biet |

I

| 1 | C6 |
| --- | --- |
| 0 | Khong |
| 99 | Khong biet |

| 1 | C6 |
| --- | --- |
| 0 | Khong |
| 99 | Khong biet |

I

|  | 269 | [visual_aid_1_vn J | Section Header: *Phan D: Anh hudng cua voe xin ly* tn/c *trimg/Shigella Diinh cho ngudi thu thiip thong tin: Tham khao phan thong tin tong quan s6* 7 *(trang tiep theo) cha phan md dau niiy. Shigella Iii vi khu6n gay tieu*  *chay nghiem tr9ng, boo gom tieu chay ra mau hoijc "kiet ly'', day Iii nguyen nhan chinh gay ro benh t/it vii t,J vong d tre* em. *Ganh nijng to/in cau ctia benh tieu chay do Shigella d tre* em *dudi* 5 *tu6i dur;1c udc tinh Iii* 75 *trieu*  *trudng hr;1p mac viJ 64.ooo trudng hr;1p tu vong hang nam, dieu nay khien*  *ly tr,;c trimglshigella trd thiinh nguyen nhiin thu hai gay ra tu vong do tieu chay [1]. C6c loai vac-xin phong ngua ly tr,;c trimg/Shigella hien dang duqc phat trien viJ c6 the duqc ung dung viio nam 2025-2030. M(!t loai voc-xin gia dinh ngila ly true trimg/Shigella du kien se duac tiem vii con m(,t hoac hai lieu cha dqt chinh, duqc tiem til thdi diem giila den cu6i cua nam dou ddi. Chi phi d,; kien duqc xem xet cua voe xin kha6ng US$1llieu vii ban dau*  *se dUr;ic ho trr;I bdi Gavi. Gia su Viet Nam se xem xet nhieu laai vac-xin de*  *duo viio su dung viio nam 2025-2030, khi vac-xin Shigella c6 kha nang duqc cung cap. (1) Khalil, The Lancet, 2018.*  Danh cho ngLloi thu th p thong tin: Tham khao phan thong tin tong quan s6 1 (trang tiep theo) cho phan mo dau nay. | descriptive | | | | | |
| --- | --- | --- | --- | --- | --- | --- | --- | --- | --- |
|  | 270 | [ d_immun_sched_vn J | 4.1 Anh/chi c6 cho rang l[ch tiem chung cua ChLldng trlnh TCMR cua Viet Nam con khoang tr6ng trong de b6 sung m9t lo9i vac xin phong ngua ly tn/c trung/Shigella hay khong? | radio, Required | | | | | |
|  |  |  |  |  | 1 | C6 | |  | |
|  |  |  |  |  |  |  | |  |  |
|  |  |  |  | 0 | | | Khong |  |  |
|  | 271 | [ d_immun_sched_yes_vn J | 4.1.1 Anh/ch[ e ng9i dieu gl ve viec b6 sung m9t lo9i vac xin mdi vao l[ch tiem chung thong thLlong cua Viet Nam? *Do not need to type the answer - audio recording will capture. | text | | | | | |
|  | 272 | [visual_aid_2_vietnam_vn]  Show the field ONLY if: [interview_location_vn] = '5' | Danh cho nghien cuu vien: Tham khao ho trc;1 tn/c quan s6 2 (trang ke ben) cho cau h6i 4.2. | descriptive | | | | | |
|  | 273 | [ d_vietnam_vaccine_priority_v n]  Show the field ONLY if: [interview_location_vn] = '5' | 4.2 T9i Viet Nam, Lide tfnh ly trl,(c trung/Shigella la nguyen nhan gay ra 6.800 truong hc;Jp tieu chay *W* trung blnh den nang va 5 ca tu' vong 6' tre em dLldi 5 tu6i moi nam. *Ude* tfnh  vac-xin Shigella se d9t hieu qua 60% d6i vdi tieu chay do ly trl,(c trung/Shigella va c6 the ngan ngua 3.925 trLlong hc;Jp tieu chay muc dQ tu' vua den nang va 3 trLlong hc;Jp tu' vong do tieu chay gay ra b6'i ly trl/c trung/Shigella hang nam 6' Viet Nam. Trong b6i canh ganh nang benh t t va tac d9ng cua vac xin nhLI tren va day c0ng khong phai la vac xin duy nhat ma Viet Nam se xem xet vao nam 2025-2030, anh/ch[ cho rang muc d9 Liu tien cua vac xin phong ly trl,(c  trung/Shigella 6' Viet Nam nhLI the nao? | radio, Required | | | | | |
|  |  |  |  | 1 | | | Uu tien cao | |  |
|  |  |  |  | 2 | | | Uu tien trung blnh | |  |
|  |  |  |  | 3 | | | Uu tien thap | |  |
|  |  |  |  | 4 | | | Khong phai la LIU tien | |  |
|  |  |  |  | - | | | | | |
|  | 274 | [ d_vaccine_priority_why_viet_ vn]  Show the field ONLY if: [interview_location_vn] = '5' | 4.2.1 T9i sao? '[d_vietnam_vaccine_priority_vn]'?*Do not need to type the answer - audio recording will capture. | text | | | | | |
|  | 275 | [ shigella_priority_slow_amr_v n] | 4.3 Trong b6i canh neu vac xin ly trl,(c trung/shigella ciuc;Jc cung Ling, anh/chj cho rang muc ciQ LIU tien nhLI the nao ve viec tiem vac xin ly trl,(c trung/Shigella cho tre em vdi kha nang lam ch m t6c dQ hoac ngan ngua tlnh tr9ng khang khang sinh t9i Viet Nam? | radio, Required | | | | | |
|  |  |  |  | 1 | | | Uu tien cao | |  |
|  |  |  |  | 2 | | | Uu tien trung binh | |  |
|  |  |  |  | 3 | | | Uu tien thap | |  |
|  |  |  |  | 4 | | | Khong phai la Liu tien | |  |
|  | 276 |  |  |  | | | | | |
|  |  | [ shigella_prioirity_amr_why_v n] | 4.3.1 T;;ii sao anh/chi II/a chon phLldng an tra loi nay? '[shigella_priority_slow_amr_vn]'? *Do not need to type the answer - audio recording will capture. | text | | | | | |
|  | 277 | [visual_aid_3_vietnam_vn]  Show the field ONLY if: [interview_location_vn] = '5' | Danh cho nghien cu'u vien: Tham khao ho trc;1 trl,(c quan so 3 va 4 (2 trang ke ben) cho cau h6i 4.4. | descriptive | | | | | |
| I 278 | | [visual_aid_4_vn J |  | descriptive | | | | | |

|  | 279 | [ d_description_3_vn J | Ngay cang c6 nhieu bang cht'.ing cha thay IY. tn,tc trung/Shigella gay ra mot so h u qua nghiem trong cha st'.ic kh6e va cuoc song cua ngLJoi benh ngoai viec gay ra dot tieu chay cap tfnh. Tieu chay do IY. tn,tc trung/Shigella c6 the gay ra si,I can tro hap th1,1 chat dinh dLJong o ruot, dan den suy dinh duong. LY. tri,lc trung/Shigella c6 lien quan den tlnh  tr;;mg coi coc, ch m Ion 6 tre em. Day cOng la mot yeu to nguy *Cd* doi voi cac truong hop tu' vong do cac benh truyen nhiem khac o tre em va tre em thap coi da duoc cht'.ing minh la c6 nguy *Cd* mac cac benh man tfnh cao hon khi trlfong thanh. Ngoai ra, thap coi c6 lien quan den tlnh tr;;mg ch m phat trien the chat, nh n tht'.ic, trlnh do hoc van thap  va cuoi cung la kha nang kiem tien thap hon khi trlfong thanh. Hay xem xet kha nang mot lo;;ii vac-xin ngan ngua IY. tri,lc trung/Shigella c6 the giup ngan ngua mot so h u qua tieu Ci/C Ve Su'C kh6e Va CUOC song. Trang phan nay Cua CUOC ph6ng van, chung toi muon tlm hieu tam quan trong cua loi fch Su'C kh6e va CUOC song Cua vac-xin nglla IY. tri,IC trung/Shigella, ngoai viec ngan ngua tieu chay cap va cac trlfong hop tu vong lien quan khi gioi thieu vac-xin ngua IY. trljc trung/Shigella so voi cac vac-xin khac ma Viet Nam c6 the xem xet vao nam 2025-2030, khi vac xin Shigella c6 kha nang dLJQ'c cung Ctng. | descriptive | | | |
| --- | --- | --- | --- | --- | --- | --- | --- |
|  | 280 | [ d_vietnam_priority_multi_vn | 4.4 T;;ii Viet Nam, IY. tri,lc trung/Shigella la nguyen nhan gay | radio, Required | | | |
|  |  | l | ra khoang 900 trlfong hQ'p suy dinh duong thap coi moi | 1 | Uu tien cao |  | |
|  |  | Show the field ONLY if: [interview_location_vn] = '5' | nam 6 tre em duoi 5 tuoi 6 mt'.ic trung blnh hoac nang.  Truoc d6, anh/chi da xem xet mt'.ic do Lfu tien cua vac-xin IY. trvc trung/Shigella la [d_vietnam_vaccine_priority_vn] khi | 2 | Uu tien trung blnh |  |  |
|  |  |  |  | 3 | Uu tien thap |  |  |
|  |  |  | xem xet ganh nang cua benh Shigella va tac dong cua vac  xin. Bay gio, ngoai tac dong doi voi tieu chay va tu' vong do IY. trijc trung/Shigella o muc do trung blnh den nang, vac xin | 4 | Khong phai la Lfu tien |  |  |
|  |  |  |  | - | | | |
|  |  |  | Shigella c6 the ngan nglla 530 trlfong hop suy dinh dLJBng |  | | | |
|  |  |  | thap coi o tre em dLJoi 5 tuoi moi nam t(li Viet Nam. Trang |  | | | |
|  |  |  | boi canh loi fch gia tang nay, anh/chi cho rang muc do LJu |  | | | |
|  |  |  | tien nhlf the nao khi gioi thieu vac xin Shigella nay t;;ii Viet |  | | | |
|  |  |  | Nam, biet rang day se khong phai la lo;;ii vac xin duy nhat |  | | | |
|  |  |  | ma Viet Nam xem xet vao nam 2025-20307 |  | | | |
| 1281 | | [ d_priority_multi_why_vn J | 4.4.1 T;;ii sao7*Do not need to type the answer - audio recording will capture. | text | | | |
|  | 282 | [ d_vietnam_priority_wage_vn | 4.5 TrLJoc d6, anh/chi da coi vac xin Shigella la uu tien [d_vietnam_priority_multi_vn] khi xem xet tac dong trong viec ngan ngua tieu chay *W* nh den nang, tt'.i vong do IY. trljc trung/shigella va coi coc ch m Ion. Bay gio, hay xem xet rang ngoai dieu nay, vac xin shigella c6 the tac dong tfch ci,lc den viec tang thu nh p cua ngLJoi Ion. Voi IQ'i fch gia tang nay, Lfu tien dat ra la gl khi gioi thieu vac xin Shigella nay t;;ii Viet Nam, biet rang day se kh6ng phai la lo;;ii vac xin duy  nhat ma quoc gia b,m xem xet vao nam 2025-20307 | radio, Required | | | |
|  |  | l |  | 1 | Uu tien cao |  | |
|  |  | Show the field ONLY if: [interview_location_vn] = '5' |  | 2 | Uu tien trung blnh |  |  |
|  |  |  |  | 3 | Uu tien thap |  |  |
|  |  |  |  | 4 | Khong phai la LJu tien |  |  |
|  |  |  |  |  | | | |
| 1283 | | [why_wage_earning_priority_ vn] | 4.5. T;;ii sao7 *Do not need to type the answer - audio recording will capture. | text, Required | | | |
|  | 284 | [ d_description_4_vn J  Show the field ONLY if: [interview_level_vn] = '1' | Shigella la chi la mot nguyen nhan gay tieu chay va neu tac dong cua vac xin doi voi viec phong ngua tieu chay dLJQ'c can nhac don le, dieu d6 c6 the lam giam chi phf-hieu qua nhll mot phllelng tht'.ic kiem soat tieu chay so voi vac xin Rotavirus va cac can thiep dinh dLJong khac, chang h;;in nhLJ bo sung Vitamin A. Tuy nhien, neu bao gom ca loi fch bo sung ve mi;it st'.ic kh6e, phat trien va kinh te lau dai cua mot vac xin Shigella tiem nang, thl chi phf-hieu qua cua vac xin se dLJoc cai thien, trang mot so trllong help, vac xin c6 these tiet kiem chi phf. | descriptive | | | |
|  | 285 | [ d_vaccine_benefits_vn J  Show the field ONLY if: [interview_level_vn] = '1' | 4.6 NhC/ng loi fch bo sung nay cua vac xin Shigella va kha nang cai thien hieu qua chi phf hoac tiet kiem chi phi cua vac xin khien anh/chi LJu tien su dt,rng vac-xin hon o mt'.ic do nao7 | radio, Required | | | |
|  |  |  |  | 2 | Uu tien nhieu hon | |  |
|  |  |  |  | 1 | Uu tien hon muc trung blnh | |  |
|  |  |  |  | 0 | Khong anh hllong | |  |
|  | 286 |  |  |  |  |  |  |
|  |  | [ d_vaccine_benefits_why_vn]  Show the field ONLY if: [interview_level_vn] = '1' | 4.6.1 T;;ii sao7 *Do not need to type the answer - audio recording will capture. | text | | | |

I

I

| 1 | Lich tiem m6'i |
| --- | --- |
| 2 | Lich tiem trung v6'i vac xin dang c6 trong chudng trlnh tiem chung thLlong quy |
| 3  - | Mc;,t trong hai phudng an tren |

| 1 | Khong anh hl.long |
| --- | --- |
| 2 | Khong san sang |
| 3 | Rat khong san sang |
| 4 | Khong xem xet lo<;1i vac xin nay |

| 1 | Khong anh hLlong |
| --- | --- |
| 2 | Khong san sang |
| 3 | Rat khong san sang |
| 4 | Khong xem xet lo9i vac xin nay |

|  | 287 | [ d_specific_price_vn]  Show the field ONLY if: [interview_level_vn] = '1' | 4.7 Anh/chi c6 the dl.la ra mllc gia c6 the chap nh n dLl<;lc d6i v6'i vac xin phong IY. tn,tc trung (nhLI chi phi cho moi lieu ho c tre da dL1<;1c tiem chung day du) de vac xin c6 dL1<;1c SI/  ung h9 vi c khi dl.la vim lich tiem chung thong thLiang? MLie gia anh/ chi dua ra la bao nhieu? Anh/chi di/a tren nhG'ng yeu t6 nao de dua ra quyet dinh nhu v y? *Do not need to type the answer - audio recording will capture. | text |
| --- | --- | --- | --- | --- |
|  | 288 | [visual_aid_5_vietnam_vn]  Show the field ONLY if: [interview_location_vn] = '5' | Section Header: *Phon E: Phan phoi voe xin Anh/chi hay tu'img tur;mg rang Viet Nam dang xem xet al.fa vaa su di,mg mot laai voe xin Shigel/a. Xin nhac lai, voc-xin di/ kien se con 1 ho¢c 2 lieu cha a<;lt chinh, au<;1c* tiem *W giiJa aen cuoi niim aou aai. Doi vai nhiJng ciiu hoi tiep theo nay, chung toi quan tiim den mong muon cua anhlchi aoi vai tinh aijc* thu *cua voe xin boo gom*  *vaa I/ch voe xin, voe xin kh6ng nguyen* arm *gi6 so vai voe xin aa gi6 va*  *audngdimg.*  Danh cho nghien cu'u vien: Tham khao ho tr<;/ trlfc quan s6 5 (trang ke ben) cho cau h6i 5.1. | descriptive |
|  | 289 | [ e_vietnam_admin_time_vn]  Show the field ONLY if: [interview_location_vn] = 5 | 5.1 Chung toi muon bat dau bang cau hoi ve l[ch tiem chung. Gia SL/ vac xin IY. trl/C trung/ Shigella la vac-xin khang nguyen ddn du<;ic tiem MOT LIEU vao THANG THLI 6 nhLI lich tiem mc;it lo<;1i vac xin m6'i hoan toan ho c THANG THLI 9 cung v6'i vac-xin Soi dL1<;1c tiem. Anh/chi thay thoi diem tiem nao phu h<;lp hdn? | radio, Required |
|  | 290 | [ e_vietnam_admin_time_why  _vn]  Show the field ONLY if: [e_vietnam_admin_time_vn]= 1 or [e_vietnam_admin_time_ vn]=2 or [e_vietnam_admin_ti me_vn]=3 and [interview_loca tion_vn]=5 | 5.1.1 T<;1i sao anh/chi l<;1i II/a chon phudng an tra loi nay? '[e_vietnam_admin_time_vn]'? *Do not need to type the answer - audio recording will capture. | text |
|  | 291 | [ e_admin_time_6mo_vn]  Show the field ONLY if: [e_bk_admin_time_vn] = '2' or [e_ghana_admin_time_vn] = '2' or [e_kenya_admin_time_v n] = '2' or [e_nepal_admin_tim e_vn] = '2' or [e_vietnam_admi n_time_vn] = '2' | 5.2 Bay gio, anh/chj hlnh dung neu vac xin tiem ddn gia, chi c6 the dLl<;lc cung cap vao l[ch tiem m6'i. 0ieu nay se anh huong nhu the nao den muc dc;i san sang cua anh/chj trong viec dua vac xin IY. trl/c trung/ Shigella vao l[ch trlnh tiem chung dinh ky? | radio, Required |
|  | 292 | [ e_admin_time_9mo_vn]  Show the field ONLY if: [e_bk_admin_time_vn] = '1' or [e_ghana_admin_time_vn] = '1' or [e_kenya_admin_time_v n] = '1' or [e_nepal_admin_tim e_vn] = '1' or [e_vietnam_admi n_time_vn] = '1' | 5.2 Bay gio, anh/chj hlnh dung neu vac xin tiem ddn gia, chi c6 the dLl<;lc cung cap vao lich tiem trung v6'i vac xin dang c6 trong chudng trlnh tiem chung thuang quy. Dieu nay se anh huong nhLI the nao den muc d9 san sang cua anh/chi trong vi c dua vac xin IY. trl/c trung/ Shigella vao l[ch trlnh tiem chung djnh ky? | radio, Required |
|  | 293 | [ e_admin_time_notpref_why_ vn]  Show the field ONLY if: [e_admin_time_6mo_vn] = '1' or [e_admin_time_6mo_vn] = '2' or [e_admin_time_6mo_vn]  = '3' or [e_admin_time_6mo_v  n] = '4' | 5.2.2 T9i sao anh/chj l9i llfa chon phLldng an tra loi nay? '[e_admin_time_6mo_vn]'?*Do not need to type the answer  - audio recording will capture. | text |
|  | 294 | [ e_admin_time_notpref_why2  _vn]  Show the field ONLY if: [e_admin_time_9mo_vn] = '1' OR [e_admin_time_9mo_vn] = '2' OR [e_admin_time_9mo_v n] = '3' OR [e_admin_time_9m o_vn] = '4' | 5.2.2 T9i sao anh/chj l9i llfa chon phLldng an tra loi nay? '[e_admin_time_9mo_vn]'?*Do not need to type the answer  - audio recording will capture. | text |

| 1 | Khong anh huong |
| --- | --- |
| 2 | Khong san sang |
| 3 | Rat khong san sang |
| 4  - | Khong xem xet loQi vac xin nay |

| 1 | Vac xin don gia (chi c6 ly trt,J'c trung/Shigella) |
| --- | --- |
| 2 | Vac xin ph6i hop (ly trt,J'c trung/Shigella va mot khang nguyen khac) |
| 0 | Khong c6 st,/ uu tien giC/a vac xin don va vac xin ph6i hop |

|  | 295 | [ e_two_doses_vn J | 5.3 Gia su' vac xin ly tn,lc trung/ Shigella don gia, yeu cau HAI lieu tiem cha dot chfnh. Yeu cau cua HAI M0I TIEM trong dot tiem chung chfnh se anh hL16'ng nhll thenao den *mCtc* do san sang cua anh/chi trong viec dlla vac xin Shigella vao lich tiem chung dinh ky? | radio, Required | | |
| --- | --- | --- | --- | --- | --- | --- |
|  | 296 | [ e_two_doses_why_vn J | 5.3.1 TQi sao anh/chi IQi 11,J'a chon phuong an tra loi nay? '[e_two_doses_vn]'? *Do not need to type the answer - audio recording will capture. | text | | |
|  | 297 | [visual_aid_7_vietnam_vn J  Show the field ONLY if: [interview_location_vn]=5  I | Danh cha nghien cClu vien: Tham khao ho tro tn,lc quan s6 6 (trang ke ben) cha cau h6i 5.4. | descriptive | | |
|  | 298 | [ e_single_dose_inject_vn J | 5.4 Gia su' Viet Nam quyet dinh cung Ling vac xin ly tn,lc trung/ Shigella tiem mot lieu duy nhat cha tre vao 9 thang tuoi trong Itch tiem chung thllong quy. Chung Su' d\rng san xuat vac xin nay la don gia hoi;ic da gia, trong d6 vac xin ly trt,J'c trung/ Shigella duoc ket hop voi mot khang nguyen khac trong mot Ian tiem. Anh/chi thfch lo9i vac xin nao hon? | radio, Required | | |
|  | 299 | [ e_single_dose_inject_why_vn  l | 5.4.1 T,;1i sao anh/chi l,;1i 11,J'a chon phuong an tra loi nay? '[e_single_dose_inject_vn]'? *Do not need to type the answer - audio recording will capture. | text | | |
|  | 300 | [ stand_alone_cascade_vn J  Show the field ONLY if: [e_single_dose_inject_vn] = 1 | 5.5 Di;ic tfnh nao sau day cua vac xin ly trt,J'c trung/ Shigella se lam thay doi *mCtc* do san sang Su' d\Jng loQi vac xin don gia (chi c6 ly trt,(c trung/Shigella): | checkbox, Required | | |
|  |  |  |  |  | stand_alone_cascade_vn_1 | Lo,;1i vac xin don gia (chi c6 ly trt,J'c trung/Shigella) ft hieu qua hon trong viec ngan ngu'a tieu chay *W* trung b1nh den ni;ing hoi;ic tu' vong so voi vac xin ph6i hop (ly trt,J'c trung/Shigella va mot khang nguyen khac |
|  |  |  |  | 2 | stand_alone_cascade_vn_2 | Lo9i vac xin don gia (chi c6 ly trt,J'c trung/Shigella) doi  h6i nhieu khoang chCta trong day chuyen l,;1nh hon so voi vac xin ph6i hop (ly trt,J'c trung/Shigella va mot khang nguyen khac |
|  |  |  |  | 3 | stand_alone_cascade_vn_3 | Lo,;1i vac xin don gia (chi c6 ly tn,lc trung/Shigella) dat hon khong nhieu so voi vac xin ph6i hop (ly trt,J'c trung/Shigella va mot khang  nguyen khac |
|  |  |  |  | stand_alone_cascade_vn_4 | | Khong c6 cai nao 6' tren |

| 1 | Khong anh huong |
| --- | --- |
| 2 | Khong san sang |
| 3 | Rat khong san sang |
| 4  - | Khong xem xet lo;ii vac xin nay |

| 1 | Khong anh huong |
| --- | --- |
| 2 | Khong san sang |
| 3 | Rat khong san sang |
| 4 | Khong xem xet losii vac xin nay |

|  | 301 | [ combo_cascade_vn]  Show the field ONLY if: [e_single_dose_inject_vn] = 2 | 5.5 Dae tfnh nao sau day cua vac xin IY. tn/c trung/ Shigella se lam thay doi muc do san sang stl dt,mg lo9i vac xin ph6i hc;1p (IY. trt,fc trung/Shigella va mot khang nguyen khac): | checkbox, Required | | |
| --- | --- | --- | --- | --- | --- | --- |
|  |  |  |  |  | combo_cascade_vn_ | Lo;ii vac xin ph6i hc;1p (IY. trt,fc trung/Shigella va mot khang nguyen khac) ft hieu qua hon  trong viec ngan ngll'a tieu chay *W* trung binh den nang hoac tu' vong so voi vac xin don gia (chi c6 IY. trtjc  tru ng/Shigella) |
|  |  |  |  | 2 | combo_cascade_vn_2 | Lo;ii vac xin ph6i hc;1p (IY. trt,fc trung/Shigella va mot khang nguyen khac) doi h6i nhieu khoang chtla trong day chuyen 19nh hon so voi vac xin don gia (chi c6  IY. trt,fc trung/Shigella) |
|  |  |  |  | 3 | combo_cascade_vn_3 | Lo;ii vac xin ph6i hc;lp (IY. trt,fc trung/Shigella va mot khang nguyen khac) dat hon khong nhieu so voi vac xin  don gia (chi c6 IY. trt,fc  tru ng/Shigella) |
|  |  |  |  | 4 combo_cascade_vn_4 | | Khong c6 cai nao *o* tren |
|  | 302 | [ stand_alone_cascade_why_v  n]  Show the field ONLY if: [stand_alone_cascade_vn(1)]  = '1' or [stand_alone_cascade_ vn(2)] = '1' or [stand_alone_ca scade_vn(3)] = '1' or [stand_al one_cascade_vn(4)] = '1' or [c ombo_cascade_vn(1)] = '1' or [combo_cascade_vn(2)] = '1' o r [combo_cascade_vn(3)] = '1' or [combo_cascade_vn(4)] = '1' | 5.5.1 T;ii sao? *Do not need to type the answer - audio recording will capture. | text | | |
|  | 303 | [ e_combo_pref_vn]  Show the field ONLY if: [e_single_dose_inject_vn] = '2' | 5.6 Gia djnh vac xin IY. trt,fc trung/ Shigella chi dlic;lc cung cap duoi d9ng Vac xin don gia (chi c6 IY. trt,fc trung/Shigella).  £lieu nay se anh huong nhli thenao den muc do san sang  cua anh/chj trong viec dua vac xin IY. trt,fc trung/ Shigella vao ljch tiem chung djnh ky? | radio, Required | | |
|  | 304 | [ e_combo_pref_2_vn]  Show the field ONLY if: [e_single_dose_inject_vn] = '1' | 5.6 Gia djnh vac xin IY. trt,fc trung/ Shigella chi duc;1c cung cap duoi d;ing Vac xin ph6i hc;lp (IY. trt,fc trung/Shigella va mot khang nguyen khac). £lieu nay se anh huong nhu the nao den muc do san sang cua anh/chj trong viec dua vac xin IY. trt,fc trung/ Shigella vao ljch tiem chung djnh ky? | radio, Required | | |
|  | 305 | [ e_combo_pref_why_vn]  Show the field ONLY if: [e_combo_pref_vn] = '1' or [e_ combo_pref_vn] = '2' or [e_co mbo_pref_vn] = '3' or [e_comb  o_pref_vn] = '4'  I | 5.6.1 T;ii sao anh/chj l9i It/a '[e_combo_pref_vn]'? *Do not need to type the answer - audio recording will capture. | text | | |
|  | 306 | [ e_combo_pref_2_why_vn]  Show the field ONLY if: [e_combo_pref_2_vn] = '1' or [e_combo_pref_2_vn] = '2' or [e_combo_pref_2_vn] = '3' or [e_combo_pref_2_vn] = '4' | 5.6.1 T;ii sao anh/chj 19i lt,fa '(e_combo_pref_2_vn]'? *Do not need to type the answer - audio recording will capture. | text | | |

| 1 | Vac xin dudng u6ng |
| --- | --- |
| 2 | Vac xin dl.ldng tiem |
| 3  - | Khong c6 sl)' Liu tien giCta vac xin dl.ldng u6ng hay dl.ldngtiem |

| 1307 | | [visuaI_7] | Danh cho nghien cuu vien: Tham khao ho trc;I tn,(c quan s6 7 (trang ke ben) cho cau h6i 5.7. | descriptive | | | |
| --- | --- | --- | --- | --- | --- | --- | --- |
|  | 308 | [ e_route_admin_only_vn] | 5.7 Gia su vac xin Shigella dl.lc;lc tiem mot lieu duy nhat khi tre 9 thang tu6i. Vac xin c6 the dl.lc;lc dung bang dl.ldng u6ng hoac dl.ldng tiem. Anh/chi mong mu6n vac xin dL1c;1c cung cap theo dl.ldng nao? | radio, Required | | | |
|  | 309 | [ e_route_admin_only_why_vn  l  Show the field ONLY if: [e_route_admin_only_vn] = '1' or [e_route_admin_only_vn] = '2' or [e_route_admin_only_v  n] = '3' | 5.7.1 Ts1i sao anh/chi ls1i h,ta chon phLlong an tra ldi nay? '[e_route_admin_only_vn]'?*Do not need to type the answer - audio recording will capture. | text | | | |
|  | 310 | [ e_oral_cascade_vn J  Show the field ONLY if: [e_route_admin_only_vn]=1 | 5.8 Dae tfnh nao sau day cua vac xin ly true trung/ Shigella se lam thay d6i muc do san sang su dung lo9i vac xin dl.ldng u6ng: | checkbox, Required | | | |
|  |  |  |  | 1 | e_oral_cascade_vn_ | Lo9i vac xin dl.ldng u6ng ft hieu qua hon trong viec ngan ngua tieu chay  tu trung blnh den nang hoac tu vong so vdi vac xin dl.ldng tiem | |
|  |  |  |  | 2 | e_oral_cascade_vn_2 | Lo9i vac xin dl.ldng u6ng doi h6i nhieu khoang chua trong day chuyen l9nh hon so vdi vac xin dl.ldng tiem | |
|  |  |  |  | 3 | e_oral_cascade_vn_3 | Los1i vac xin dl.ldng u6ng dat hon khong nhieu so  vdi vac xin dLldng tiem | |
|  |  |  |  | 4 e_oral_cascade_vn_4 | | Khong c6 cai nao *o* tren | |
|  | 311 | [ e_injectable_cascade_vn]  Show the field ONLY if: [e_route_admin_only_vn]=2 | 5.8 Dae tfnh nao sau day cua vac xin ly true trung/ Shigella se lam thay doi muc do san sang su dung lo9i vac xin dl.ldng tiem: | checkbox, Required | | | |
|  |  |  |  | 1 | e_injectable_cascade_vn_1 | | Lo9i vac xin dl.ldng tiem ft hieu qua hon trong viec ngan ngua tieu chay tu trung blnh den nang hoac tu vong so vdi vac xin dl.ldng u6ng |
|  |  |  |  | 2 | e_injectable_cascade_vn_2 | | Los1i vac xin dl.ldng tiem doi h6i nhieu khoang chua trong day chuyen l9nh hon so vdi vac xin dl.ldng u6ng |
|  |  |  |  | 3 | e_injectable_cascade_vn_3 | | Loi;li vac xin dLldng tiem dat hon khong nhieu so vdi vac xin dl.ldng  u6ng |
|  |  |  |  | 4 e_injectable_cascade_vn_4 | | | Khong c6 cai nao *o*  tren |

| 1 | Khong anh huong |
| --- | --- |
| 2 | Khong san sang |
| 3 | Rat khong san sang |
| 4  - | Khong xem xet los1i vac xin nay |

| 1 | Khong anh huong |
| --- | --- |
| 2 | Khong san sang |
| 3 | Rat khong san sang |
| 4 | Khong xem xet los1i vac xin nay |

| 1 | Khong anh huong |
| --- | --- |
| 2 | Khong san sang |
| 3 | Rat khong san sang |
| 4 | Khong xem xet los1i vac xin nay |

| 1 | Khong anh huong |
| --- | --- |
| 2 | Khong san sang |
| 3 | Rat khong san sang |
| 4 | Khong xem xet los1i vac xin nay |

| 312 | [ e_oral_injectable_cascade_w hy_vn]  Show the field ONLY if: [e_oral_cascade_vn(1)] = '1' or [e_oral_cascade_vn(2)] = '1' or [e_oral_cascade_vn(3)] = '1' or [e_oral_cascade_vn(4)] = '1' or [e_injectable_cascade_vn(1)] = '1' or [e_injectable_cascade_v n(2)] = '1' or [e_injectable_cas cade_vn(3)] = '1' or [e_injectab le_cascade_vn(4)] = '1' | 5.8.1 T;ii sao? *Do not need to type the answer- audio recording will capture. | text |
| --- | --- | --- | --- |
| 313 | [ e_oral_only_vn]  Show the field ONLY if: [e_route_admin_only_vn] = 2 | 5.9 Gia su' vac xin ly tn,/c trung/Shigella chi duoc cung cap theo duong u6ng. Dieu nay se anh huong nhu the nao den muc do san sang cua anh/chi trong viec dua vac xin ly tn,/c trung/ Shigella vao l[ch tiem chung dinh ky? | radio, Required |
| 314 | [ e_injectable_only_vn]  Show the field ONLY if: [e_route_admin_only_vn] = 1 | 5.9 Gia *sa* vac xin ly tn,/c trung/Shigella chi duoc cung cap  theo duong tiem. Dieu nay se anh huong nhu the nao den muc do san sang cua anh/chi trong viec dua vac xin ly tn,/c trung/ Shigella vao lich tiem chung dinh ky? | radio, Required |
| 315 | [ e_route_admin_only_2_why_ vn]  Show the field ONLY if: [e_oral_only_vn] = '1' or [e_ora l_only_vn] = '2' or [e_oral_only  _vn] = '3' or [e_oral_only_vn] =  '4' | 5.9.1 T;ii sao anh/chi ls1i 11,/a ch<;>n phudng an tra loi nay? '[e_oral_only_vn]'? *Do not need to type the answer - audio recording will capture. | text |
| 316 | [ e_route_admin_only_2_why_ 2_vn]  Show the field ONLY if: [e_injectable_only_vn] = '1' or [e_injectable_only_vn] = '2' or [e_injectable_only_vn] = '3' or [e_injectable_only_vn] = '4' | 5.9.1 Ts1i sao anh/chi ls1i 11,/a chon phudng an tra loi nay? '[e_injectable_only_vn]'? *Do not need to type the answer - audio recording will capture. | text |
| 317 | [ e_description_2_vn] | Neu mot so d c tfnh khac cua vac xin ly trl,/c trung/Shigella c6 the tac dong den viec phan ph6i vac xin. Gia djnh Viet Nam dang xem xet de gioi thieu mot los1i vac xin ly trl,/c trung/ Shigella la vac xin khang nguyen ddn theo duong tiem, dl19c tiem mot lieu khi tre 6 thang tuoi ho c 9 thang tuoi. | descriptive |
| 318 | [ e_lyophilized_vn] | 5.10 Vac xin ly trl,/c trung/Shigella duoc dong kho va can dl1c;1c pha ls1i trl16'c khi su d1,mg. Dieu nay se anh huong nhl1 the nao den muc do san sang cua anh/chi trong viec dua vac xin ly trl,/c trung/ Shigella vao lich trlnh tiem chung dinh ky? | radio, Required |
| 319 | [ e_lyophilized_why_vn] | 5.10.1 T;ii sao anh/chi ls1i 11,/a chon phudng an tra loi nay? '[e_lyophilized_vn]'? *Do not need to type the answer - audio recording will capture. | text |
| 320 | [ e_packaged_single_dose_vn] | 5.11 Vac xin ly trl,/c trung/ Shigella gia djnh duoc dong g6i du6'i ds1ng mot lieu duy nhat *(We* la cac 6ng tiem duoc chua san vac xin trong xilanh de san sang tiem). Dieu nay se anh  huong nhu the nao den muc do san sang cua anh/chi trong viec dua vac xin ly trl,/c trung/ Shigella vao lich trlnh tiem chung djnh ky? | radio, Required |
| 321 | [ e_packaged_single_dose_wh y_vn] | 5.11.1 Ts1i sao anh/chi ls1i 11,/a chon phudng an tra loi nay? '[e_packaged_single_dose_vn]'? *Do not need to type the answer - audio recording will capture. | text |

|  | 322 | [ e_booster_year_2_vn] | 5.12 Vac xin ly tn/c trung/ Shigella gia djnh yeu cau tiem nhac l9i mot lieu trong nam thu' 2 cua cuoc doi. Dieu nay se anh huong nhu the nao den mu'c do san sang cua anh/chj  trong viec dua vac xin ly tn/c trung/ Shigella vao ljch trlnh tiem chung dinh ky? | radio, Required | | | | | |
| --- | --- | --- | --- | --- | --- | --- | --- | --- | --- |
|  |  |  |  | 1 | | | Khong anh huong | |  |
|  |  |  |  | **2** | | | Khong san sang | |  |
|  |  |  |  | 3 | | | Rat kh6ng san sang | |  |
|  |  |  |  | 4  - | |  | Khong xem xet lo9i vac xin nay | |  |
|  | 323 | [ e_booster _year_2_why_vn] | 5.12.1 T.,i sao anh/chj I9i I1,1a chon phucmg an tra loi nay? '[e_booster_year_2_vn]'? *Do not need to type the answer- audio recording will capture. | text | | | | | |
|  | 324 | [ e_storage_neg_20_vn] | 5.13 Vac xin yeu cau bao quan *a* nhiet do -20°C hoac thap  hon. Dieu nay se anh huong nhu the nao den mu'c do san sang cua anh/chi trong viec dua vac xin ly tr1,1c trung/ Shigella vao ljch trlnh tiem chung djnh ky? | radio, Required | | | | | |
|  |  |  |  | 1 | | | Khong anh huong | |  |
|  |  |  |  | 2 | | | Khong san sang | |  |
|  |  |  |  | 3 | | | Rat kh6ng san sang | |  |
|  |  |  |  | **4** | | | Khong xem xet lo9i vac xin nay | |  |
|  | 325 |  |  |  | | | | | |
|  |  | [ e_storage_neg_20_why_vn] | 5.13.1 T.,i sao anh/chi l.,i I1,ia chon phuong an tra loi nay? '[e_storage_neg_20_vn]'? *Do not need to type the answer - audio recording will capture. | text | | | | | |
|  | 326 | [ e_other_attributes_vn] | 5.14 C6 bat ky dac diem nao khac cua vac xin shigella gia djnh c6 the anh huong den mu'c do san sang cua anh/chj trong viec dua vac xin ly tr1,1c trung/ Shigella vao lich trlnh tiem chung dinh ky? Dae tfnh gl? Tai sao? *Do not need to type the answer - audio recording will capture. | text | | | | | |
|  | 327 | [f_sub_nat_intro_vn] | Section Header: *Phan F: C6c tlu tien gi6i thieu vac xin m6i 6 cop qu6c gia*  *Day Iii phan cu6i cilng cua cu6c ph6ng van. Doi v6i ba cau h6i cu6i cilng nay, hiiy tt16ng ttl<;tng rang Viet Nam c6 ty* le *nhiem Ix trt/c trilng!Shigelfa*  *kh6c nhau giiJa c6c khu v,;c hoiic c6 xu ht16ng bung ph6t Ix tr,;c triJng/Shigeffa hang nam 6 mot so khu Vt/C nhat dinh. Danh gi6 viec gi6i thieu vac xin Shigeffo tren phom vi toan qu6c, hay chi 6 cac khu v,;c c6 ty le mac benh cao hoijc nht1mot bien ph6p kiem soot 6 dich.*  6.1 Anh/chi c6 ung ho viec gioi thieu vac xin ly trl,lc trung/ Shigella *a* cap qu6c gia khong? | radio, Required | | | | | |
|  |  |  |  |  | 1 | C6 | |  | |
|  |  |  |  | 0 | | | Khong |  |  |
|  |  |  |  |  | | | | | |
| 1328 | | [why_do_not_need_to_type_t h_vn] | 6.1.1 T.,i sao?*Do not need to type the answer - audio recording will capture. | text | | | | | |
|  | 329 | [f_sub_nat_acceptability_vn] | 6.2 Anh/chi tien luong nhung van de gl ve kha nang chap nhan khi cung cap vac xin phong ly trl,lc trung/Shigella tren ph.,m vi toan qu6c? *Do not need to type the answer -  audio recording will capture.  *G<;ti y: cha c6c nha hooch dinh chinh s6ch, nh{m vi!!n y te, va c6c thanh vi!!n cong dong?* | text | | | | | |
|  | 330 | [f_sub_nat_feasibility_vn] | 6.3 Anh/chi tien ILJQ'ng nhiJng van de gl lien quan den tfnh kha thi khi van hanh, phan ph6i va cung Lfng vac xin phong ly trl,lc trung/Shigella tren ph.,m vi toan qu6c? *Do not need  to type the answer - audio recording will capture.  *G<;ti y: cha c6c nhli hooch dinh chinh s6ch, nhan vi!!n y te, vii c6c thlinh vien cong dong?* | text | | | | | |
|  | 331 | [g_other_comments_vn J | Section Header: *Phan G: Ket thuc ph6ng van*  7.1 Ngoai cac chu de da dLJQ'c ban luan trong cuoc ph6ng van nay, anh/chi c6 de cap den van de gl khac lien quan c6 the anh huong den mu'c do san sang trong viec dua vac xin  phong ly tr1,1c trung/Shigella vao ljch trlnh tiem chung djnh ky *a* Viet Nam kh6ng? *Do not need to type the answer -  audio recording will capture. | text | | | | | |
|  | 332 | [ interview_guide_vietnamese  _complete J | Section Header: *Form Status*  Complete? | dropdown | | | | | |
| Instrument: **INTERVIEW GUIDE - NEPALI** (interview_guide_nepali)  _3 [unique_id_nepal] 1 0.:: - text, Required | | | | | | | | | |
|  |  | | |  | | | | | |
|  | 334 | [recording_confirmation_nep al] | X.1 'lfm? | ired | | | | | |

I

| 0 | Incomplete |
| --- | --- |
| 1 | Unverified |
| 2  - | Complete |

335

1336

I

337

I I 338

339

[ recording_start_script_nepal

# l

[ interview_date_nepal]

[ interview_location_nepal]

[ city_town_district_nepal] [ interview_level_nepal]

- 1. ffl :

3ffcTT(,[ lfl <llcFf:!mi I [unique_lD_nepal]

- 1. :
  2. ftrcfi

X4.1 ;,n:f:

- 1. :

descriptive

text (date_dmy), Required Field Annotation: @TODAY

radio, Required

1 1:f>ffit

1. 'ilRT

3

4

5 1{(R]ll

text, Required

radio, Required

1:1= 1

340

341

[ hcf_level_nepal]

Show the field ONLY if: [interview_level_nepal] = '2'

[ hcf_publicvprivate_nepal]

Show the field ONLY if: [interview_level_nepal] = '2'

- - 1. cll:lT{W"'f llT
    2. i[fcl,f.ti;ft?

radio, Required 1

2 cam

3 -am

radio, Required

l:l ffi I

342

343

344

345

346

347

348

349

350

[ hcf_name_nepal l

Show the field ONLY if: [interview_level_nepal] = '2'

[a_org_nepal]

[ a_title_nepal]

[a_years_experience_nepal]

[a_nitag_icc_nepal] Show the field ONLY if:

[interview_level_nepal] = '1' or [hcf_level_nepal] = '3'

[ b_u5_health_concerns_nepal

# l

[ b_u5_health_concern_nepal]

[ b_u5_health_concern_why_n epal]

[ b_interv_priority_nepal]

- - 1. ;,n:f:

Section Header: *'l.fTTT A: wi.rrfi'rii'r <RgTT?f*

- 1. W"'f cf>llFTjg ? * Do not need to type the answer - audio recording will capture.
  2. / if? *Dnoneedtto type the answer - audio recording will capture.
  3. q,fct *Dnnoeed tto type the answer - audio recording will capture.
  4. NITAG err I CC cfTT err f.'rffeR! Ifi

ocfi$¥l-ll'l-f1Tf

Section Header: *'l.fTTTB: "f rr&ITfffitrr*

2.1 "$Jl'it cm iilIB o!RllTIR< *w* 1ff

1 £ ¢0I□1ollc, l@l{ llll\ ollciollf'acfilcfil N lj?,I$¥¢0Io!RllT<ilcffiR

*Do not need to type the answer - audio recording will capture.

- 1. llT l\ 'ljf.'rc!,1 oliil1$¥cf;I

tRsl@Tffi<ITT W?

- - 1. fw-:P. *Do not need to type the answer - audio recording will capture.

*3{lfw,,* g'f *i'f*

2.3 tR9@T OO"cfiT ffi1fl errffi1fl , mm

m Jfft./R 1Tj ? *Do not need to type the answer - audio recording will capture.

*"&r:r(itcri-trfm. . mrrRf I* flrf

text, Required, Identifier

text, Identifier

text, Identifier

text, Identifier

illuired

text

radio, Required

| 1 | lJUfu: |
| --- | --- |
| 2 | lJUfu: ir. ('Rrjti;f |
| 3  - | JPl lj?,1$¥¢1 lJUfu: |

text

text

|  | 351 | [ b_importance_growth_nepal  l | 2.4 ol"fil ofii:ITct>1 fc!ct>m < fl  mITT? | radio, Required | | |
| --- | --- | --- | --- | --- | --- | --- |
|  |  |  |  |  | 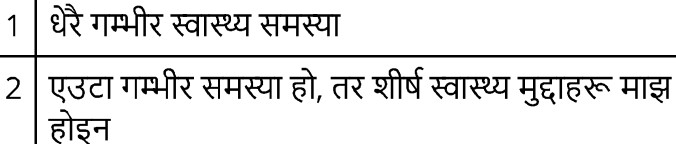 | |
|  |  |  |  |  | 3 ,3p:r lt;'(<sct,1 1Jl'l-!Rm  - | |
|  | 352 | [ b_importance_growth_why_ nepal] | 2.4.1 fc!,.,? *Do not need to type the answer - audio recording will capture.  *g?"if g"f ff "i'R/1tiT>WR; ill .wrm,g?"if*  *g"fff* | text | | |
|  | 353 | [ b_heard_of_shigella_nepal] | 2_5i);" o!RlTT £9? | 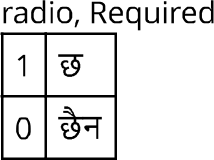 | | |
|  | 354 | [shigella_awareness_nepal]  Show the field ONLY if: [b_heard_of_shigella_nepal] = '1' | 2.6 isldl'3je;01 , fcl;- o!RlTT i);- 'l-fm? *Do notneed to type the answer - audio recording will capture. *Probe: burden/high-risk populations, source of transmission, diagnosis, symptoms, seque/ae, and treatment* | text | | |
|  | 355 | [ b_shigella_importance_nepal  l  Show the field ONLY if: [b_heard_of_shigella_nepal] = '1' | 2.7 <RI l$llTT ol"fil ?I isllciisi1Rlct>1$·M>l <.'Wlt lf)Tf m W"f  W"f £91? | radio, Required  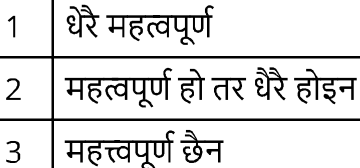  99 | | |
|  | 356 | [ b_shigella_aspects_nepal]  Show the field ONLY if: [b_heard_of_shigella_nepal] = '1' | 2.8 W"f cf "ciTfrr fcl1;m ITT?  *Do not need to type the answer - audio recording will capture.  *fR!!Tfff-<tit rrfttrcr , ml'<T'fF:J ill*  *I* | text | | |
|  | 357 | [ b_shigella_priority_nepal]  Show the field ONLY if: [b_heard_of_shigella_nepal] = '1' | 2.9 "@{[ I  isli'i:lI(l'(ict,I <.'Wlt W@T fcm;<914 ct>l<R1lHI-JI i);­  m ? | radio, Required  1"'3"i'i:l""ITT  2 lCf£.lflll=f"ITT 3"cf>Tl"ITT  4>11 | | |
|  | 358 | [ c_amr_importance_nepal] | Section Header: *'ffeT C: ef.t fR!!Tfff -<tit u1TrlT< -<tit 1Ffi iRT<PT ef.t*  *-<tit"i.JITlWTTR-<tit ffl1ff "itt lit ef.t efffi!Fr fFx{T[ft*  *1Ffi* [9"/  3.1 1-11 i1>1Fcl<1a c!?rm  "ITT? | radio, Required | | |
|  |  |  |  | 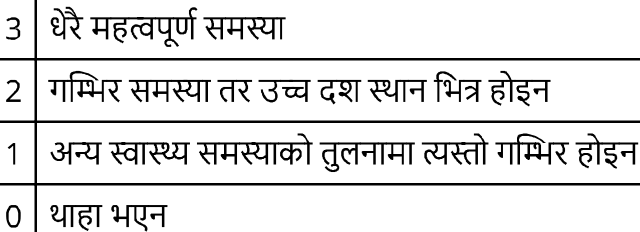 | |  |
| 359  '---1'--- | | [c_amr_importance_why_nep al]  I | 3.1.1 *Do not need to type the answer - audio recording will capture. | text | | |
|  | 36 | [0c_amr_treatment_costs_nep al]  Show the field ONLY if: [interview_level_nepal] = '2' | 3.2 ol"lillm 1-ll it>lRlllci RI-R el iizj'  / l:IR"cf>"tITT"ct>T ? | radio, Required  1 "ITTl:IR"cf>"  0  99 '1-[(f! | | |
|  | 361 | [ c_amr_treatment_costs_yes_ nepal]  Show the field ONLY if:  [ c_amr_treatment_costs_nepa I]= '1' | 3.2.1 <ITT-Rt? *Do not need to type the answer - audio recording will capture. | text | | |
|  | 362 | [ c_amr_ns_priority_nepal]  Show the field ONLY if: [interview_level_nepal] = '1' | 3.3 Is information on anti-microbial resistance of a target pathogen taken into consideration when prioritizing the introduction of a new vaccine against that pathogen in [interview_location]? | radio, Required | | |

I

| 1 Yes | |
| --- | --- |
| 0 | No |
| 99 | Don't know |

| 1 | m 3ff£ITTlTT |
| --- | --- |
| 2 | .:r&f!l'l'Im 3ff£ITTlTT |
| 3 | m 3ff£ITTlTT |
| 4 | m w,, |

| 1 | m |
| --- | --- |
| 2 | .:r&f!l'l'Im |
| 3 | m |
| 4 | m w,, |

| 363 | [ c_amr_ns_priority_yes_nepal  l  Show the field ONLY if: [c_amr _ns_priority_nepal] =  '1' and [interview_level_nepal]  = '1' | 3.3.1 How? *Do not need to type the answer - audio recording will capture. | text |
| --- | --- | --- | --- |
| 364 | [ c_amr_ns_priority_no_dn_ne pal]  Show the field ONLY if: [c_amr_ns_priority_nepal] = 'O' or [c_amr _ns_priority_nepa I]= '99' | 3.3.1 Is it criteria that needs to be considered? *Do not need to type the answer - audio recording will capture. | text |
| 365 | [visual_aid_1_nepal] | Section Header: *'l-fTlTD: 1Ii/rriitrr'Jffef 1Ef: p![[[{*  *3/ifilqufTff I 1T<JcTFuicrT1!J"flffiq'i6[*  *'Rs/Tffl,* ™ *,-,jt"flT'it "flT'/T </l<'l</Jff/¢Ji, ¢) ffl'ft* i/?"*Of*  *g'f.f I "lft p'f r:rf.'r i31R?Ji/ 'IJP/tii,Mli efitrn*  *mf I -,jtFf'Rs/Tffl "fTf 'fTTett <ll<'l</Jff/¢/i,MI{ Ff*  *%2 1 fij-flo[lllit <!Tcpr TR!fTffTifit* 19¼ *ftifcrlH,*  *.riff.;* & *l{ooo <fiT 1FJ ,f I </Tc '1/f!<liT 1FJ lRslTffl </Tc Ff*  *1FJ cft!!IT 'i7f.nFit* Ef *I , rfezrmT Ff 1FJ 'RT*  *<l"it"fl?l<i'R/W ,'1/jrq Jf:FIT<f/Rqif/Rqi,f* /  *&Tl '9,<*?*F*o?¼*f*- ?o o *,p:J:f pr {ij-1/ffe{*  Ef *I iT<JcT 1Ii/rrilft* W*fij-, 'l7'IT<liT* ' *fFti <!ef <l@ilff/¢Ji,Mli qf .Jf'ff* ffi *I iT<JcT 'l7'IT<liT 1Ii/rrilft1JflI fFti 6ffl'* r:r-fu",*'llftrcit '{F1!T 1R*Ef *I*  *u:irfcHR 'RslTffl¢"'1"1T'f"efrl<lil w 'gRT<lil 'T'f*  *°fl I "fll<f/; f ti"* ?o?t - ?o o fri?: *i#" <'lfi'rW<it*  W *<!I'f &Tl Ilffl<lil"fJ< '1JflI '*  *,if/cwh "ofl?ITT'fCP<'f,r,jrf.f 1(1) Khalil, The Lancet, 2018.* | descriptive |
| 366 | [ d_immun_sched_nepal] | 4.1 il,wn{ fcl, f.'rqfmf M' f.trimr  'l-Rc!Rill JfRl\fU[ *'l-R*  f.'r!:ffmf TRqiT -arfu | ooquired |
| 367 | [ d_immun_sched_yes_nepal] | 4,1,1 'l-Rc!Rill JfRl\fUT 'l-R mfmf TRq,1-arfu *Do not  need to type the answer - audio recording will capture. | text |
| 368 | [visual_aid_2_nepal_nepal]  Show the field ONLY if: [interview_location_nepal] = '4' | 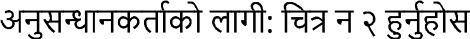 | descriptive |
| 369 | [ d_nepal_vaccine_priority_ne pal]  Show the field ONLY if: [interview_location_nepal] = '4' | 4.2 t:[]WlP1Tfi'rffi<TTc F Q?'5 I 1-sIQ@i:11¢1 d  *\!(,ooo,(,* iJR'lqTTlfcll: oflSf 1 cillcicillfacf51(';'6¢'1  I 3f:FlR fcl,f.trimr "<oo/o-mmxur "cf,sf  cpp:[1JITT I <RI *x\900(,*  'cilTcciiilk! 1.9'1,\!\!uRT<ITTlfcll:oNf.'r I  <RIi\Trct,) ' "cill  <A xox - xo o f.trimr 'l<l1fcRR *¥,* m 3ff£ITTlTT  "i:IPJTR ?f<A? | radio |
| 370 | [d_vaccine_priority_why_ne_n epal]  Show the field ONLY if: [interview_location_nepal] = '4' | 4.2.1mfiA<flR17:t 'l-!<TT: '[d_nepal_vaccine_priority_nepal]'?  *Do not need to type the answer - audio recording will capture. | text |
| 371 | [shigella_priority_slow_amr_n epal] | 4.3 lTT f.trimr m g-TTRGff@ Q-i!lqllflf2cf5 l-ll sf>lfclllci fmq,1"ffi1fiqtjm I? | radio, Required |
| 372 | [shigella_prioirity_amr_why_n epal] | 4.3.1 lTT fiA<flR17:t 'l-!<TT:  '[shigella_priority_slow_amr_nepal]'? *Do not need to type the answer - audio recording will capture. | text |
| 373 | [visual_aid_3_nepal_nepal]  Show the field ONLY if: [interview_location_nepal] = '4' | For investigator only: Please use visual aids #3 and #4 for question 4.4 preamble. | descriptive |

I

| I 374 | | [visual_aid_4_nepal] |  | descriptive | | | | |
| --- | --- | --- | --- | --- | --- | --- | --- | --- |
|  | 375 | [ d_description_3_nepal] | F -llRo[ -3Pi"  «lIBII 1:!f.'rfcRd'R 1.9 Iollciollfact>ll-11  Fl:Rg"@@ <ffiR. fctct>mq,i::rF, 31m1:!R 1.9 I7:R! "Affil  «lIBII 31mq»f TRi:rcrcl TR I 7:R[  fcp- llRo! "ci'JT!tq,fct 4'I$GIIJ1--lct> 1_9'l-f'R I <TTfi, $llslq@a cfmcl:RfolTc s'f m •fcl;!<TTc "1°1%3-ji:f,1 wi'.f Jf"--<l 4>1{GIIJ1--lct>cil{ J:l£ZR"R TR  -3Pi" "ct,T o l\- o o <'!'--l" <TT  °@"q "'fQlW!Hf4T '<ITR I <'!'--1" o l\ - o o  i)1tlffll F 3fcIB!Ts.-J  "@"q FITT I | descriptive | | | | |
|  | 376 | [ d_nepal_priority_multi_nepal  l  Show the field ONLY if: [interview_location_nepal] =  '4' | 4.4 fur@IBT[('j l\"ollST ollciollfact>ll-11 '<, oo J:l&jl:!1'.[ "ct,sf $1151q lcil I  3fcIBIT m "3"ii:f  1.9 I <TT {,oo l\ "ollST ollciollfact>l(l-!>cil{  s.-Jm I <TI 4'il{Gltl·<>cil{ "!£ZR"R <TT  W--1" m 3ff'ilWTT "ffilJF "(jjo[  o l\- o o <TT ofW<!,-3Pi" t!f.'r  FITT I | radio, Required | | | | |
|  |  |  |  | 1 | "3"ii:fm |  | | |
|  |  |  |  | 2 | J:l&jl:!1'.[>11 |  |  |  |
|  |  |  |  | 3 | m |  |  |  |
|  |  |  |  | -4 | m ITT |  |  |  |
|  |  |  |  |  | | | | |
| 1377 | | [ d_priority_multi_why_nepal] | 4.4.1 fcp-.-J? *Do not need to type the answer - audio recording will capture. | text | | | | |
|  | 378 | [ d_nepal_priority_wage_nepal  l  Show the field ONLY if: [interview_location_nepal] =  '4' | 4.5 F . '  mmmFcl>ftr 11m1m Tf<'J3!1Zr t!f.'r "CfTT$c:TF €t<'!'--l" o l\- o om-3Pi""@"q  m m s--1 | radio, Required | | | | |
|  |  |  |  | 1 | "3"ii:fm | |  | |
|  |  |  |  | 2 | J:l&jl:!1'.[>11 | |  |  |
|  |  |  |  | 3 | q,i::rm | |  |  |
|  |  |  |  | 4 | W "ls'f | |  |  |
| 1379 | | [why_wage_earning_priority_ nepal] | 4.5.1 fcp-.-J? *Do not need to type the answer - audio recording will capture. | text, Required | | | | |
|  | 380 | [ d_description_4_nepal]  Show the field ONLY if: [interview_level_nepal] = '1' | 'l-fIBT i::rT51cf>Rq,19-71:TT?;<TT  'l-f<TT 'l-R cR m "@"q tflTRl ,i'l-ITTft s--1 m  1-fl{TT! , -3P1" "@"q cR "ciT4T  «lIBII "CfTT$cIT Wci" tflTRl  >!'4lof 'i./R s'f I | descriptive | | | | |
|  | 381 | [ d_vaccine_benefits_nepal]  Show the field ONLY if: [interview_level_nepal] = '1' | 4.6 <TT "CfTT$cIT c!Zl! fc,;,ct,i 'l-f!fc@ tflTRl ,!'l{lo[,  m m f&, | radio, Required | | | | |
|  |  |  |  | 2 | i)1 i)1Wl-ffcRcTT |  | | |
|  |  |  |  | 1 | J:l&jl:!1'.[ Wl-ffcRcTT |  |  |  |
|  |  |  |  | 0 | 3RR |  |  |  |
|  | 382 |  |  |  | | | | |
|  |  | [ d_vaccine_benefits_why_nep al]  Show the field ONLY if: [interview_level_nepal] = '1' | 4.6.1 fcp-.-J? *Do not need to type the answer - audio recording will capture. | text | | | | |
|  | 383 | [ d_specific_price_nepal]  Show the field ONLY if: [interview_level_nepal] = '1' | 4.7 f.'rfua1F<l l.9"(jj"(lTlB"'cIloRfi'q@l.i::rT5[f"ct,T"@"q I gR f.'rfua ¥l gc:T<TT  "@"q4] TR IW--1" cf>Rq, d(q FITT cf>Rq, Tf<'J <TT TR cfT f.tom TR 1R *Do not  need to type the answer - audio recording will capture. | text | | | | |
|  | 384 | [visual_aid_5_nepal_nepal]  Show the field ONLY if: [interview_location_nepal] =  '4' | Section Header: *1-fl17 E: &l1T<lft e:rrft [[i3cf rrf«ffc'RT'Til Fc/,*  *fWflT &rl ,-,.f f/Ti/iit tt if.kt ll'rlilrrcit* f-? *1ff;ll1l! q-,f I ,3/qiT"f! #i""tft 'lf'f lffrft ,i/qq,) 'l)t@/1 ¢) ;if 6fRllT &T<r 7Rt &l1T<lft [[i3c{ ef.c;iR n'IT &rr* <*&rr"ff1lITT*  *6fRlffl*  "ci'JT\t: --ll\ | descriptive | | | | |
|  | 385 | [ e_nepal_admin_time_nepal]  Show the field ONLY if: [interview_location_nepal] = 4 | 5.1 I want to start by asking you about the vaccine schedule. Assume Shigella vaccine is a single antigen vaccine given by injection, that requires ONE DOSE for the primary series at either 6 MONTHS, which requires a new vaccine visit, or 9 MONTHS when MR vaccine already administered. Operationally, what administration time point do you prefer? | radio, Required | | | | |
|  |  |  |  | 1 | 6 months of age/ new vaccine visit | | |  |
|  |  |  |  | 2 | 9 months of age/ existing vaccine visit | | |  |
|  |  |  |  | 3 | Either 6 or 9 months of age | | |  |
|  |  |  |  |  | | | | |

I

I

|  | 386 | [ e_nepal_admin_time_why_n epal]  Show the field ONLY if: [e_nepal_admin_time_nepal]= 1 or [e_nepal_admin_time_ne pal]=2 or [e_nepal_admin_tim e_nepal]=3 and [interview_loc ation_nepal] = 4 | 5.1.1 1il,;p:rr \TIR 'l-/<fl:  '[e_nepal_admin_time_nepal]'?*Do not need to type the answer - audio recording will capture. | text | | |
| --- | --- | --- | --- | --- | --- | --- |
|  | 387 | [ e_admin_time_6mo_nepal]  Show the field ONLY if: [e_nepal_admin_time_nepal]  = '1' | 5.2 rrtrJi'l,m<91tf 1-{lfcl,T  m '[e_nepal_admin_time_nepal]' I  <91tr ffi1Jrf.f <91tr ,nf rcITT1::rnn::rmr1F! gi, 1t.;'<'a1{  JRR I | radio, Required | | |
|  |  |  |  | 1 | JRR |  |
|  |  |  |  | 2 | l'f£.llllll cl q,1'f 1-{lfcl,T |  |
|  |  |  |  | 3 | q,l'f i{lfcl,T |  |
|  |  |  |  | 4  - | m <91tr m ITT |  |
|  | 388 | [ e_admin_time_9mo_nepal]  Show the field ONLY if: [e_nepal_admin_time_nepal]  = '2' | 5.2 rrtrJi'l,m<91tf i{lfcl,T  m '[e_nepal_admin_time_nepal]' I  <91tf "c1TlJ1R <91tf 1FT gi<iti'<'ci1{  JRR I | radio, Required | | |
|  |  |  |  | 1 | JRR |  |
|  |  |  |  | 2 | l'f£.llllll cl q,1'f 1-{lfcl,T |  |
|  |  |  |  | 3 | q,l'f i{lfcl,T |  |
|  |  |  |  | 4 | m <91tr m ITT |  |
|  | 389 |  |  |  | | |
|  |  | [ e_admin_time_notpref_why_ nepal]  Show the field ONLY if: [e_admin_time_6mo_nepal] = '1' or [e_admin_time_6mo_ne pal] = '2' or [e_admin_time_6 mo_nepal] = '3' or [e_admin_ti me_6mo_nepal] = '4' | 5.2.1 fcl;-;r <IT\TIR'l-/<fl: '[e_admin_time_6mo_nepal]'?*Do not need to type the answer - audio recording will capture. | text | | |
|  | 390 | [ e_admin_time_notpref_why2  _nepal]  Show the field ONLY if: [e_admin_time_9mo_nepal] = '1' or [e_admin_time_9mo_ne pal] = '2' or [e_admin_time_9 mo_nepal] = '3' or [e_admin_ti me_9mo_nepal] = '4' | 5.2.1 fcl;-;r <IT\TIR'l-/<fl: '[e_admin_time_9mo_nepal]'?*Do not need to type the answer - audio recording will capture. | text | | |
|  | 391 | [ e_two_doses_nepalJ | 5.3 m m <91tr 1-{lfcl,T,  lllWffiR rt <91tf °ITT I rt  <91tr 111¢  111 ? | radio, Required | | |
|  |  |  |  | 1 | JRR |  |
|  |  |  |  | 2 | l'f£.llllll q,1'f 1-{lfcl,T |  |
|  |  |  |  | 3 | q,l'f i{lfcl,T |  |
|  |  |  |  | 4 | m <91tr m ITT |  |
| 1392 | | [ e_two_doses_why_nepal] | 5.3.1 fcl;-;rm\TIR 'l-/<fl: '[e_two_doses_nepal]'? *Do not need to type the answer - audio recording will capture. | text | | |
|  | 393 | [visual_aid_6_nepal_nepal J  Show the field ONLY if: [interview_location_nepal] = '4' | ffilfl:fcr::r"'f r.. | descriptive | | |
|  | 394 | [ e_single_dose_inject_nepal J | 5.4 Let's suppose that [interview_location_nepal] has decided to provide an injectable, single dose primary series Shigella vaccine at 9 months of age into the routine immunization schedule. The vaccine is available in either a single antigen presentation or as part of a combination vaccine, in which Shigella is paired with another antigen in a single injection. Which vaccine presentation would you prefer? | radio, Required | | |
|  |  |  |  | 1 | A single antigen vaccine (Shigella only) | |
|  |  |  |  | 2 | A combination vaccine (Shigella plus another antigen) | |
|  |  |  |  | 0 | No preference between single and combination vaccines | |
|  |  |  |  |  | | |
|  | 395 | [ e_single_dose_inject_why_ne pal] | 5.4.1 fcl;-;rm\TIR 'l-l<fl: '[e_single_dose_inject_nepal]'? *Do not need to type the answer - audio recording will capture. | text | | |

I

|  | 396 | [ stand_alone_cascade_nepal]  Show the field ONLY if: [e_single_dose_inject_nepal] = 1 | 5.5 Which of the following attributes would change your willingness to consider introducing a single antigen presentation Shigella vaccine. Select all that apply. | checkbox, Required | | | |
| --- | --- | --- | --- | --- | --- | --- | --- |
|  |  |  |  | 1 | stand_alone_cascade_nepal_1 | | The single antigen vaccine is moderately less effective at preventing moderate to severe diarrhea and/or deaths compared to the combination vaccine |
|  |  |  |  | 2 | stand_alone_cascade_nepal_2 | | The single antigen vaccine requires moderately more 2-8°C cold chain space compared to the combination vaccine |
|  |  |  |  | 3 | stand_alone_cascade_nepal_3 | | The single antigen vaccine is moderately more expensive compared to the combination vaccine |
|  |  |  |  | 4 stand_alone_cascade_nepal_4 | | | None of the above |
|  | 397 | [ combo_cascade_nepal]  Show the field ONLY if: [e_single_dose_inject_nepal] = 2 | 5.5 Which of the following attributes would change your willingness to consider introducing a combination presentation Shigella vaccine? Select all that apply. | checkbox, Required | | | |
|  |  |  |  | 1 | combo_cascade_nepa1_1 | The combination vaccine is moderately less  effective at | |
|  |  |  |  |  |  | preventing | |
|  |  |  |  |  |  | moderate to severe  diarrhea and/or | |
|  |  |  |  |  |  | deaths compared to | |
|  |  |  |  |  |  | the single antigen vaccine | |
|  |  |  |  | 2 | combo_cascade_nepa1_2 | The combination | |
|  |  |  |  |  |  | vaccine requires | |
|  |  |  |  |  |  | moderately more 2-  80C cold chain | |
|  |  |  |  |  |  | space compared to | |
|  |  |  |  |  |  | the single antigen vaccine | |
|  |  |  |  | 3 | combo_cascade_nepal_3 | The combination | |
|  |  |  |  |  |  | vaccine is | |
|  |  |  |  |  |  | moderately more | |
|  |  |  |  |  |  | expensive | |
|  |  |  |  |  |  | compared to the | |
|  |  |  |  |  |  | single antigen vaccine | |
|  |  |  |  | 4 combo_cascade_nepal_4 | | None of the above | |

| 1 | Would not affect interest |
| --- | --- |
| 2 | Moderately less willing to consider this vaccine |
| 3 | Much less willing to consider this vaccine |
| 4  - | Would not consider this vaccine |

| 1 | Would not affect interest |
| --- | --- |
| 2 | Moderately less willing to consider this vaccine |
| 3 | Much less willing to consider this vaccine |
| 4 | Would not consider this vaccine |

| 1 | &q |
| --- | --- |
| 2 | cfTc WTm' &q |
| 3 | c!T &qlJT W I |

| 398 | [ stand_alone_cascade_why_n epal]  Show the field ONLY if: [stand_alone_cascade_nepal (1)] = '1' or [stand_alone_casc ade_nepal(2)] = '1' or [stand_a lone_cascade_nepal(3)] = '1' o r [stand_alone_cascade_nepal (4)] = '1' or [combo_cascade_n epal(1)] = '1' or [combo_casca de_nepal(2)] = '1' or [combo_c ascade_nepal(3)] = '1' or [com bo_cascade_nepal(4)] = '1' | 5.5.1 fcl;";,? *Do not need to type the answer - audio recording will capture. | text |
| --- | --- | --- | --- |
| 399 | [ e_combo_pref_nepal]  Show the field ONLY if: [e_single_dose_inject_nepal] = '2' | 5.6 Now imagine that the Shigella vaccine is only offered as single antigen vaccine (Shigella only). How would this affect your willingness to consider introducing a Shigella vaccine as part of the routine immunization schedule? | radio, Required |
| 400 | [ e_combo_pref_2_nepal]  Show the field ONLY if: [e_single_dose_inject_nepal] = '1' | 5.6 Now imagine that the Shigella vaccine is only offered as a combination vaccine (Shigella plus another antigen). How would this affect your willingness to consider introducing  a Shigella vaccine as part of the routine immunization schedule? | radio, Required |
| 401 | [ e_combo_pref_why_nepal]  Show the field ONLY if: [e_combo_pref_nepal] = '1' or [e_combo_pref_nepal] = '2' or [e_combo_pref_nepal] = '3' or [e_combo_pref_nepal] = '4' | 5.6.1 fcl;-;,i:n'3W 1-f!TT: '[e_combo_pref_nepal]'?  *Do not need to type the answer - audio recording will capture. | text |
| 402 | [e_combo_pref_2_why_nepal]  Show the field ONLY if: [e_combo_pref_2_nepal] = '1' or [e_combo_pref_2_nepal] = '2' or [e_combo_pref_2_nepal]  = '3' or [e_combo_pref_2_nep al]= '4' | 5.6.1fcl;-;,i:n'3W 1-f!TT: '[e_combo_pref_2_nepal]'?  *Do not need to type the answer - audio recording will capture. | text |
| 403 | [visual_aid_7_nepal_nepal]  Show the field ONLY if: [interview_location_nepal] = 4 | cillft:mcr\9 | descriptive |
| 404 | [ e_route_admin_only_nepal] | 5.7 J'.fRT <TT &q'<, ollciollf'dcf>l$'6cil  &q f.'\ur!:t Tf"!TT I <TT &q m'cf>T llTc!T  cfTc I <TT W"'1lJT£r!lClq]c f3c!>  "ITTci1 \iffiTT ffi"l£9 ? | radio, Required |
| 405 | [e_route_admin_only_why_ne pal]  Show the field ONLY if: [e_route_admin_only_nepal] = '1' or [e_route_admin_only_ne pal] = '2' or [e_route_admin_o nly_nepal] = '3' | 5.7.1fcl;-;,i:n'3W 1-f!TT:  '[e_route_admin_only_nepal]'?*Do not need to type the answer - audio recording will capture. | text |

|  | 406 | [ e_oral_cascade_nepal]  Show the field ONLY if: [e_route_admin_only_nepal]= 1 | 5.8 Which of the attributes below would change your willingness to consider an oral Shigella vaccine? Select all that apply. | checkbox, Required | | | |
| --- | --- | --- | --- | --- | --- | --- | --- |
|  |  |  |  | 1 | e_oral_cascade_nepal_1 | The oral vaccine is moderately less effective at preventing moderate to severe diarrhea and/or deaths compared to the injectable vaccine | |
|  |  |  |  | 2 | e_oral_cascade_nepa1_2 | The oral vaccine requires moderately more 2-8°C cold chain space compared to the injectable vaccine | |
|  |  |  |  | 3 | e_oral_cascade_nepa1_3 The oral vaccine is | | |
|  |  |  |  |  |  | moderately more expensive compared to the injectable  vaccine | |
|  |  |  |  | 4 e_oral_cascade_nepal_4 | | None of the above | |
|  | 407 | [ e_injectable_cascade_nepal]  Show the field ONLY if: [e_route_admin_only_nepal]= 2 | 5.8 Which of the attributes below would change your willingness to consider an injectable Shigella vaccine? Select all that apply. | checkbox, Required | | | |
|  |  |  |  | 1 | e_injectable_cascade_nepal_1 | | The injectable vaccine is moderately less effective at preventing moderate to severe  diarrhea and/or deaths compared to the oral vaccine |
|  |  |  |  | 2 | e_injectable_cascade_nepal_2 | | The injectable vaccine requires moderately more 2-8°( cold chain space compared to the oral vaccine |
|  |  |  |  | 3 | e_injectable_cascade_nepal_3 | | The injectable vaccine is moderately more expensive compared to the oral  vaccine |
|  |  |  |  | 4 e_injectable_cascade_nepal_4 | | | None of the above |
|  | 408 | [ e_oral_injectable_cascade_w hy_nepal]  Show the field ONLY if: [e_oral_cascade_nepal(1)] = '1' or [e_oral_cascade_nepal(2)] = '1' or [e_oral_cascade_nepal  (3)] = '1' or [e_oral_cascade_n epal(4)] = '1' or [e_injectable_c ascade_nepal(1)] = '1' or [e_inj ectable_cascade_nepal(2)] = '1' or [e_injectable_cascade_n epal(3)] = '1' or [e_injectable_c ascade_nepal(4)] = '1' | 5.8.1 fcp-;, ?*Do not need to type the answer- audio recording will capture. | text | | | |

|  | 409 | [ e_oral_only_nepal J | 5.9 '3fo! fuit.rr 1¥1o11c 1mc1>mt  "Q"'l{ffc@rrcfu- fct,- fuit;rr mfi'@  f.'m'd? | | radio, Required | | | |
| --- | --- | --- | --- | --- | --- | --- | --- | --- |
|  |  | Show the field ONLY if: |  |  | 1 | <llffi |  | |
|  |  | [e_route_admin_only_nepal] = |  |  |  |  |  |  |
|  |  |  |  |  | 2 | lC[&[l:l1'[ |  |  |
|  |  | 2 |  |  |  |  |  |  |
|  |  |  |  |  | 3 | cf»! |  |  |
|  |  |  |  |  | 4  - | -tj\ fcl'c!R |  |  |
|  | 410 | [ e_injectable_only_nepal J | 5.9 Now imagine that the Shigella vaccine is only offered as an injectable vaccine. How would this affect your willingness to consider introducing a Shigella vaccine as part of the routine immunization schedule? | | radio, Required | | | |
|  |  | Show the field ONLY if: [e_route_admin_only_nepal] = 1 |  |  | 1 | Would not affect interest | |  |
|  |  |  |  |  | 2 | Moderately less willing to consider this vaccine | |  |
|  |  |  |  |  | 3 | Much less willing to consider this vaccine | |  |
|  |  |  |  |  | 4 | Would not consider this vaccine | |  |
|  |  |  |  |  |  | | | |
|  | 411 | [ e_route_admin_only_2_why_ nepal] | 5.9.1 fcl,.,-tj\ 'l-fm: '[e_oral_only_nepal]'? *Do not need to type the answer - audio recording will capture. | | text | | | |
|  |  | Show the field ONLY if: [e_oral_only_nepal] = '1' or [e_ oral_only_nepal] = '2' or [e_or al_only_nepal] = '3' or [e_oral_ only_nepal] = '4' |  | |  | | | |
|  | 412 | [ e_route_admin_only_2_why_ 2_nepal J  Show the field ONLY if: [e_injectable_only_nepal] = '1' or [e_injectable_only_nepal] = '2' or [e_injectable_only_nepa I] = '3' or [e_injectable_only_n epal] = '4' | 5.9.1 fcl,.,-tj\ 'l-fm: '[e_injectable_only_nepal]'?  *Do not need to type the answer - audio recording will capture. | | text | | | |
|  | 413 | [ e_description_2_nepal J | fuit.rr fcl>iltiialt;,/,  fuit;rr lCfr::IT  'l-fml | ITT I rmfct;-  WT@:!-q;f <,-'I, il1iillt;,/,cf>l | descriptive | | | |
|  | 414 | [ e_lyophilized_nepal] | 5.10 ,3ffiffi""x m"ffi | | radio, Required | | | |
|  |  |  |  |  | 1 | <llffi |  | |
|  |  |  |  |  | 2 | lC[&[l:l1'[ |  |  |
|  |  |  |  |  | 3 | cf»! |  |  |
|  |  |  |  |  | 4 | -tj\ fcl'c!R |  |  |
| 1415 | | [ e_lyophilized_why_nepal J | 5.10.1 f<A-tj\ 'l-fm: '[e_lyophilized_nepal]'? *Do not need to type the answer - audio recording will capture. | | text | | | |
|  | 416 | [ e_packaged_single_dose_ne pal] | 5.11 -tj\  m mfi'lcl | Ujjq) fcl'clR  i-r-f? | radio, Required | | | |
|  |  |  |  |  | 1 | <llffi |  | |
|  |  |  |  |  | 2 | lC[&[l:l1'[ |  |  |
|  |  |  |  |  | 3 | cf»! |  |  |
|  |  |  |  |  | 4 | -tj\ fcl'c!R |  |  |
|  | 417 |  |  |  |  | | | |
|  |  | [ e_packaged_single_dose_wh y_nepal J | 5.11.1 -tj\ f<A"i'jl:R1Tj  1-fm: '[e_packaged_single_dose_nepal]'? *Do not need to type the answer - audio recording will capture. | | text | | | |
|  | 418 | [ e_booster_year_2_nepal J | 5.12 fuit.rr *"CITT9* crrm WT@:!-q;f 1  crql{cf>mt .mfi'@ | | radio, Required | | | |
|  |  |  |  |  | 1 | <llffi |  | |
|  |  |  |  |  | 2 | lC[&[l:l1'[ |  |  |
|  |  |  |  |  | 3 | cf»! |  |  |
|  |  |  |  |  | 4 | -tj\ fcl'c!R |  |  |
|  | 419 |  |  |  |  | | | |
|  |  | [ e_booster _year_2_why_nepal  l | 5.12.1 m fcA "i'jl:R1Tj1-fm:'[e_booster _year_2_nepal]'?  *Do not need to type the answer - audio recording will capture. | | text | | | |

I

I

|  | 420 | [ e_storage_neg_20_nepal] | 5.13 <TT - oc c@lJ:!if.f I <TT"@([  f.'fl:ffe@"citq 78 {iffi i);-*WI* | radio, Required | | | |
| --- | --- | --- | --- | --- | --- | --- | --- |
|  |  |  |  | 1 | <9ffil:fR"q, | |  |
|  |  |  |  | 2 | T[&[l:l1'[ | |  |
|  |  |  |  | 3 | q;i::r | |  |
|  |  |  |  | 4  - | m fcl'c!R | |  |
|  | 421 | [ e_storage_neg_20_why_nepa I] | 5.13.1 <TT'3"'dx fcl;-;, 'q"!:r,'f lJ:f 'l-f<TT: '[e_storage_neg_20_nepal]'?  *Do not need to type the answer - audio recording will capture. | text | | | |
|  | 422 | [ e_other_attributes_nepal] | 5.14 3R>g, Rl !ildl(l{> W,:,\iRffiIIT f.'fl:ffe@"citq  1R?i:ft 1-Ri);-i);-g-, *Do not need to type the answer - audio recording will capture. | text | | | |
|  | 423 | [f_sub_nat_intro_nepal] | Section Header: *'JffeTF: "fff?:&Cl<i/ '7ft*  *;/rrrCTRT fiJf½ fiff½ rmr CT!Tlft"cfT*  *f.lfim fitrimlcit 1:llJf I f.trilm &Cl<i/ "ffT1JcfiT "ffT{*  *JfTfJR1'llf.i "iI"i'if "f{q'iT[(Jf <'IPf "cfT 'l{[!ctit i/q;-wmr w:wr TRi1iT*  *"fl77ft fcrcff<ruw*  6.1 i),- «Rl'llt!fl]78 | radio, Required | | | |
| 1424 | | [why_do_not_need_to_type_t h_nepal] | 6.1.1 *Do not need to type the answer - audio recording will capture. | text | | | |
|  | 425 | [f_sub_nat_acceptability_nep al] | 6.2 m "tllfficm m «Rl'IT  -rr-:fu?r dT'tt? *Do not need to type the answer - audio  recording will capture.  *-;f/fi/ f.i'lfotcpr/1&./<-, fflfi't; I t/i;tl/&./<-cp/*  *"ffrfi'r?* | text | | | |
|  | 426 | [f_sub_nat_feasibility_nepal] | 6.3 m"@([ m 78, 1mRor 78 Wl-ffcxIBf qjl i),- i),- g-7  ? *Do not need to type the answer - audio recording will capture.  *-;f/fi/ f.iJ./10/cp(/1Ef./<-, fflfi't; I <i<l<4('./<-<PI*  *"ffrfi'r?* | text | | | |
|  | 427 | [g_other_comments_nepal] | Section Header: *'JffeT G: JRfclRfT "f/J./T[(f*  7.1 <RI $.  1-fJ: fct;- ofRlll'l-ffi  illtll'j§-0? *Do not need to type the answer - audio recording will capture. | text | | | |
|  | 428 | [ interview_guide_nepali_com plete] | Section Header: *Form Status*  Complete? | dropdown | | | |
|  |  |  |  | 0 | Incomplete |  | |
|  |  |  |  | 1 | Unverified |  |  |
|  |  |  |  | 2  - | Complete |  |  |
