# Supplementary material for: Exploring Shigella vaccine priorities and preferences: Results from a mixed-methods study in low- and middle-income settings
Source: Vaccine X. 2023 Aug 9;15:100368. doi: 10.1016/j.jvacx.2023.100368 (PMC10457597; doi:10.1016/j.jvacx.2023.100368)

**Supplemental material 2. Figure 1 country-specific results: Importance of given health issues, by stakeholder group**


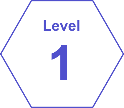
**
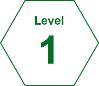
**

| National stakeholders (NS) |  | Healthcare providers (HP) |  |
| --- | --- | --- | --- |
| How important is each health issue? |  | How important is each health issue? |  |

Burkina Faso (NS n=7; HP n=13)


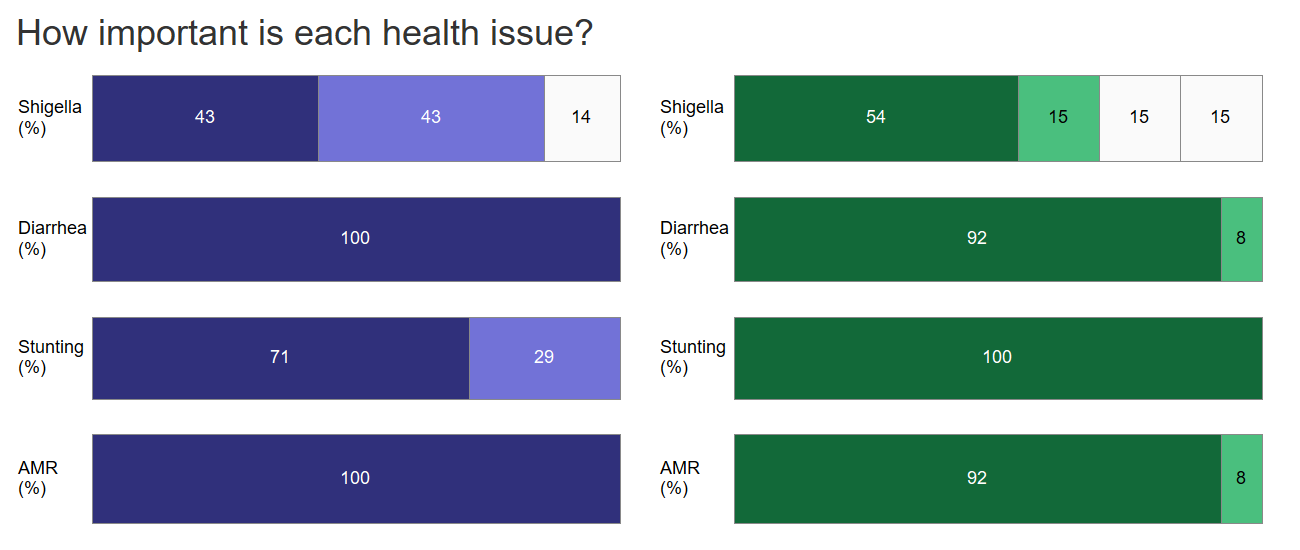


Ghana (NS n=6; HP n=11)


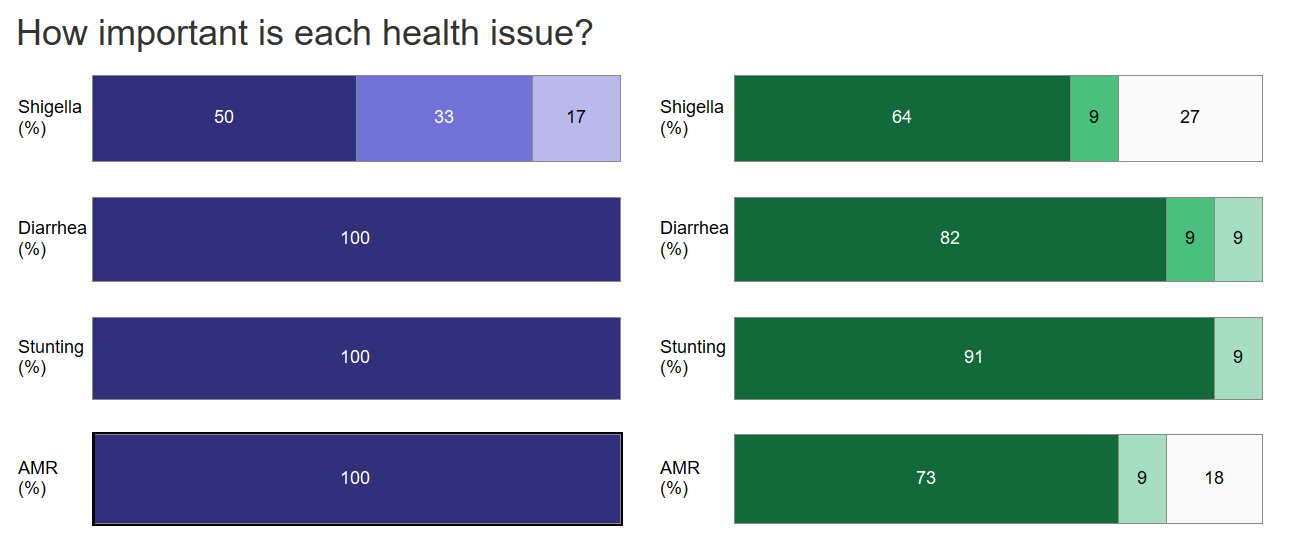


Kenya (NS n=5; HP n=10)


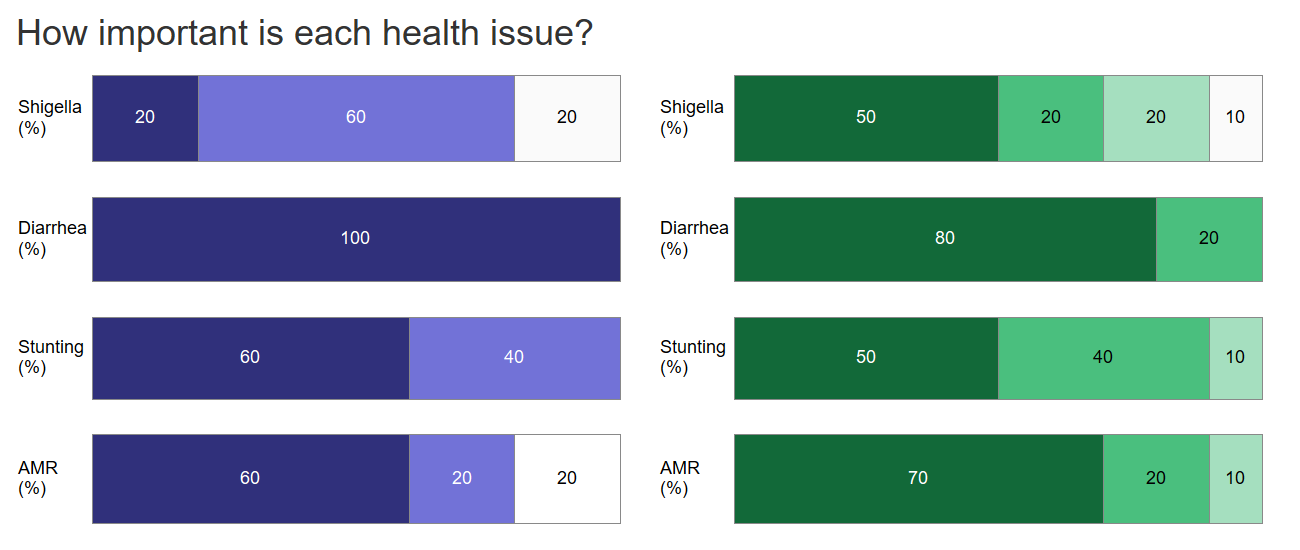


Nepal (NS n=5; HP n=10)
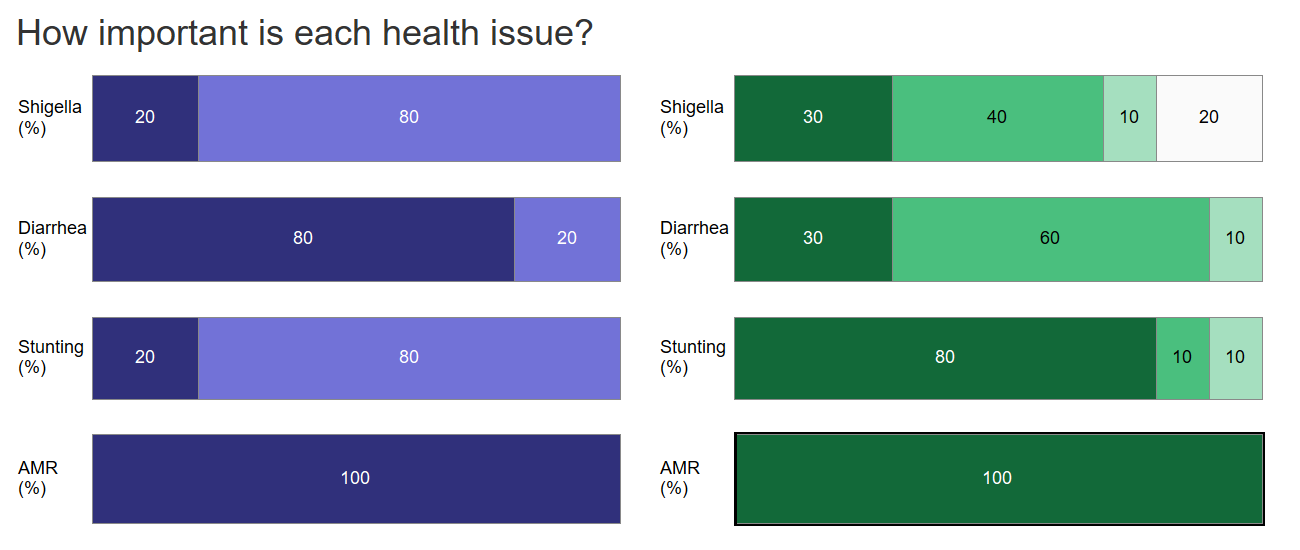


Vietnam (NS n=9; HP n=10)


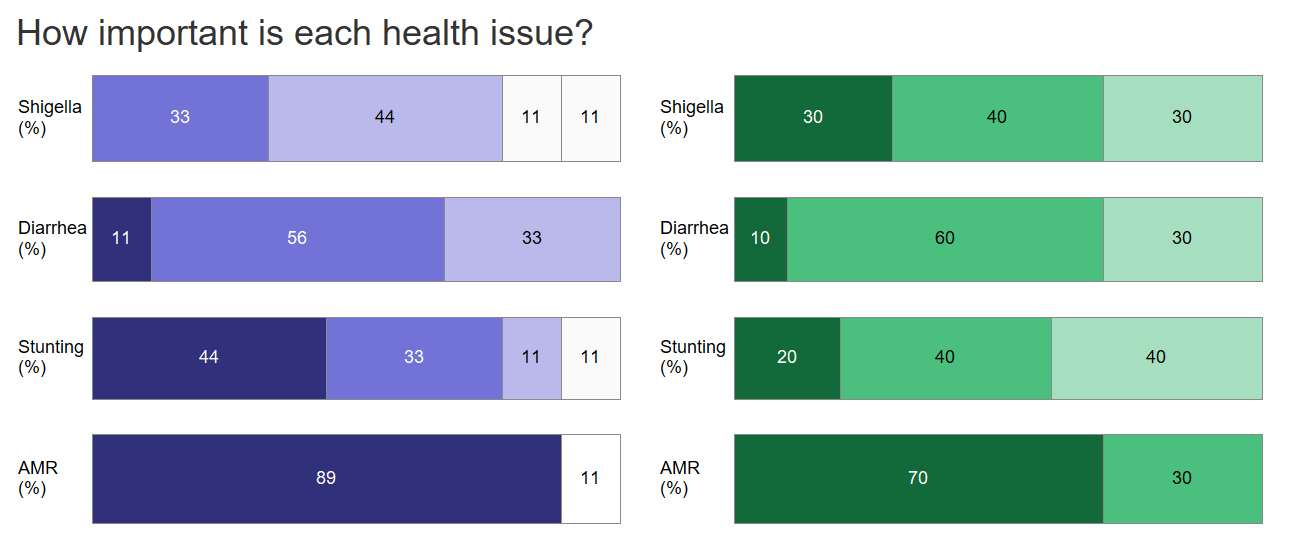


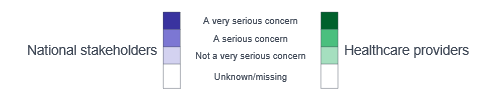

Supplement: Supplementary data 2 [file mmc2.docx]
