# Supplementary material for: Exploring Shigella vaccine priorities and preferences: Results from a mixed-methods study in low- and middle-income settings
Source: Vaccine X. 2023 Aug 9;15:100368. doi: 10.1016/j.jvacx.2023.100368 (PMC10457597; doi:10.1016/j.jvacx.2023.100368)

**Supplemental material 3. Figure 2 country-specific results: Importance of *Shigella*, by stakeholder group**


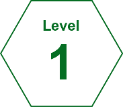

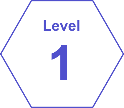


| National stakeholders (NS) |  | Healthcare providers (HP) |  |
| --- | --- | --- | --- |
| How important is *Shigella* as a health concern? |  | How important is *Shigella* as a health concern? |  |

Burkina Faso (NS n=7; HP n=13)


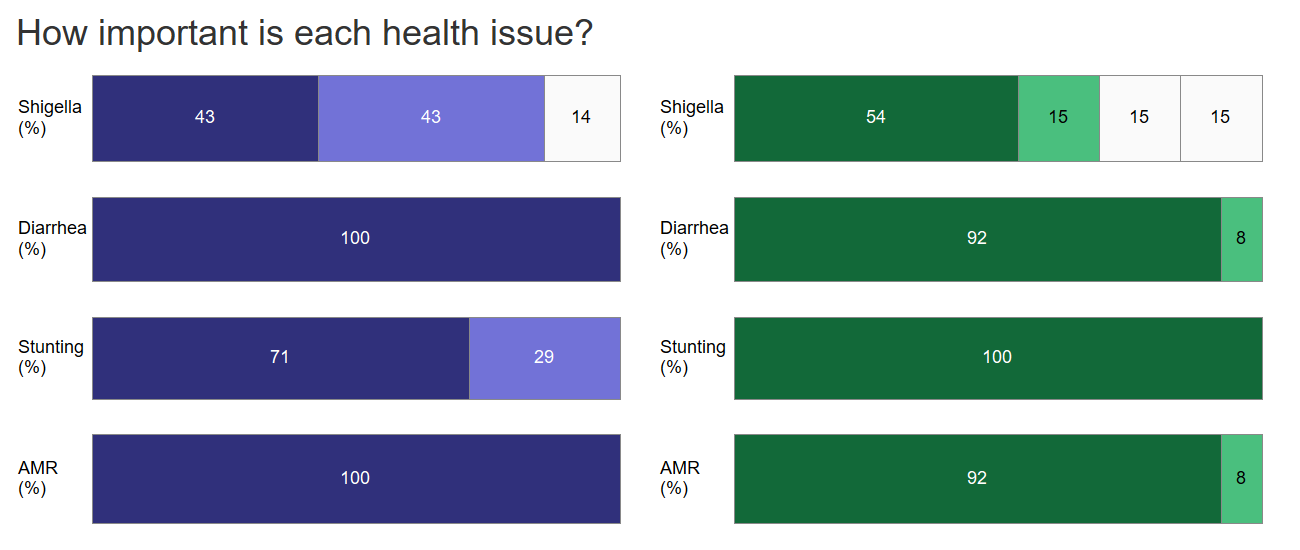


Ghana (NS n=6; HP n=11)


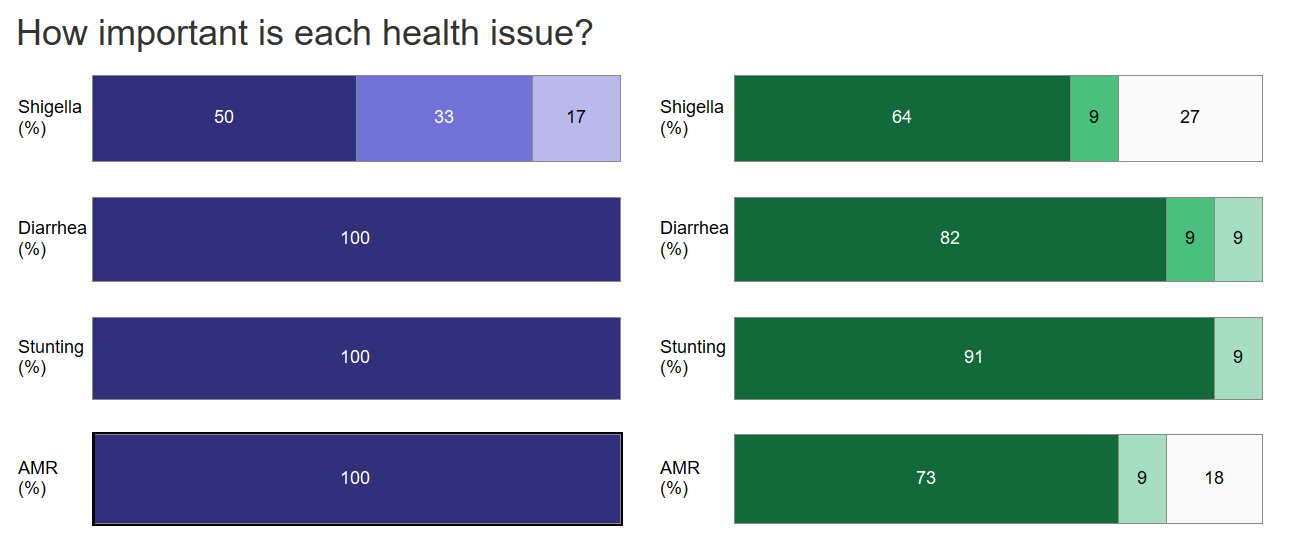


Kenya (NS n=5; HP n=10)


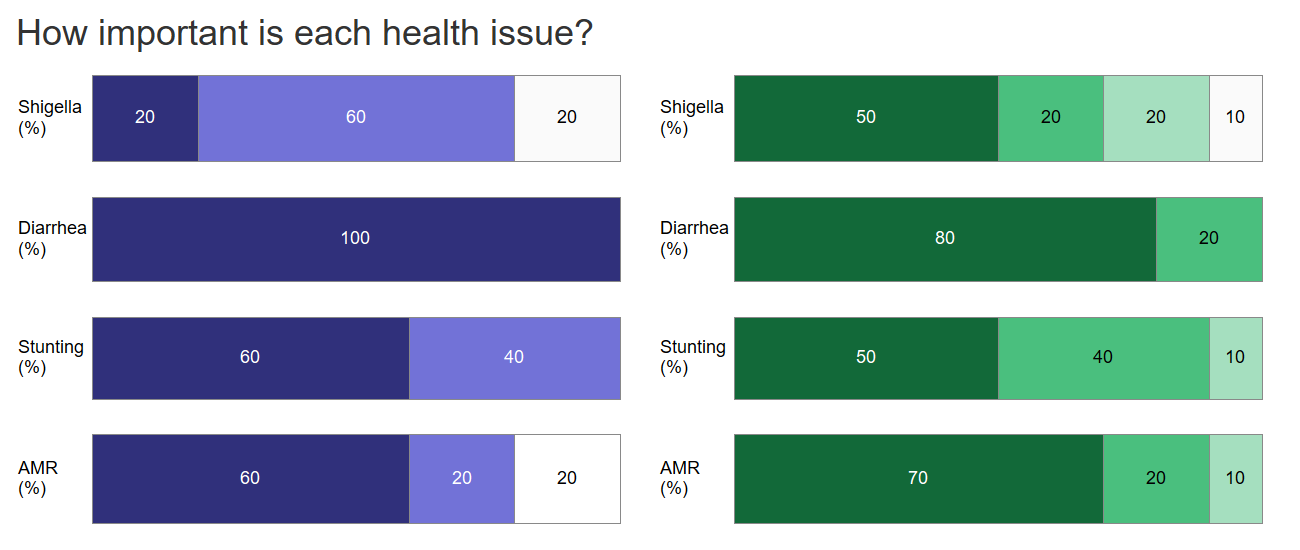


Nepal (NS n=5; HP n=10)


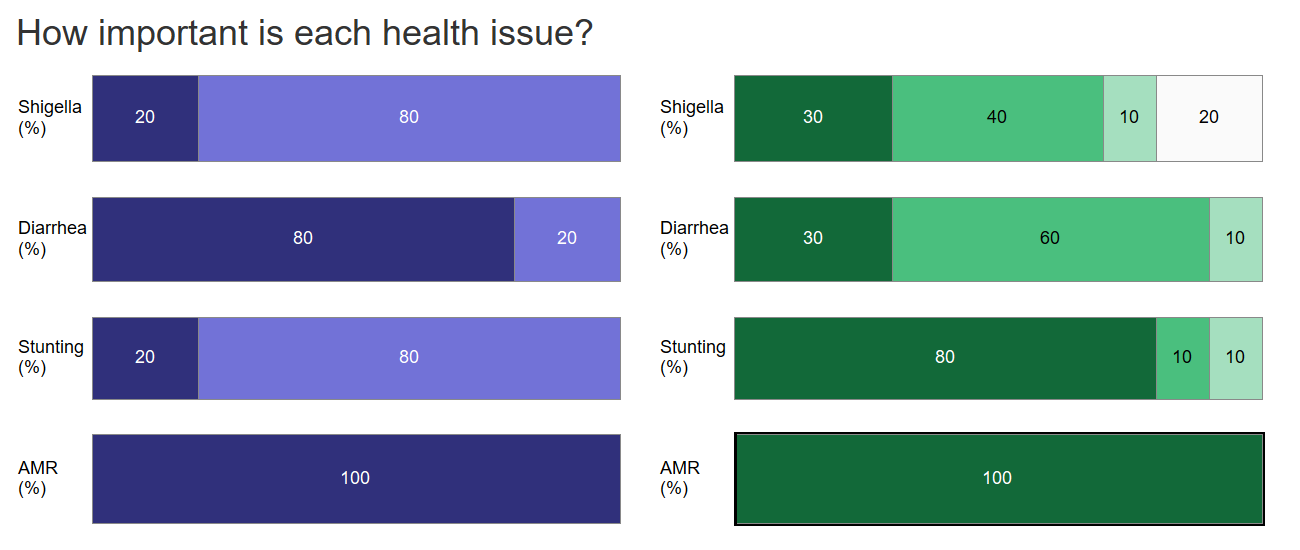


Vietnam (NS n=9; HP n=10)


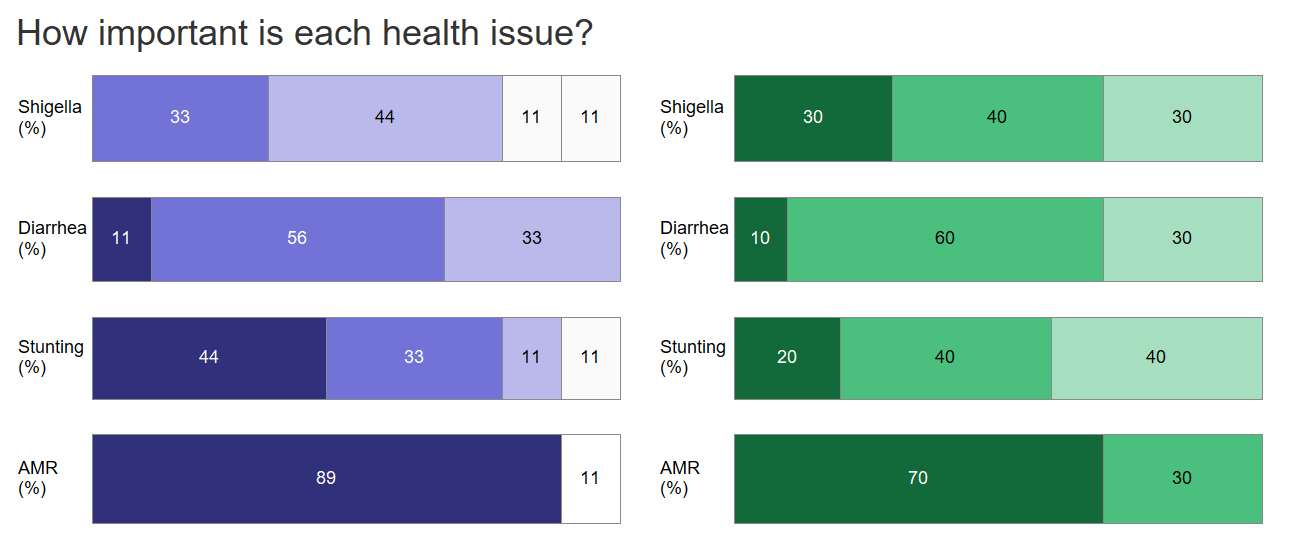


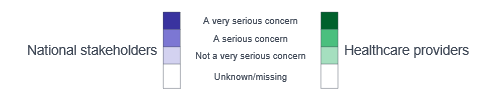

Supplement: Supplementary data 3 [file mmc3.docx]
