# Supplementary material for: Exploring Shigella vaccine priorities and preferences: Results from a mixed-methods study in low- and middle-income settings
Source: Vaccine X. 2023 Aug 9;15:100368. doi: 10.1016/j.jvacx.2023.100368 (PMC10457597; doi:10.1016/j.jvacx.2023.100368)
Supplement: Supplementary data 5 [file mmc5.docx]

**Supplemental material 5. Figure 4 country specific results: preferred route of administration, vaccine presentation, and administration timing, by stakeholder group (%)**

Burkina Faso (NS n=7; HP n=13)


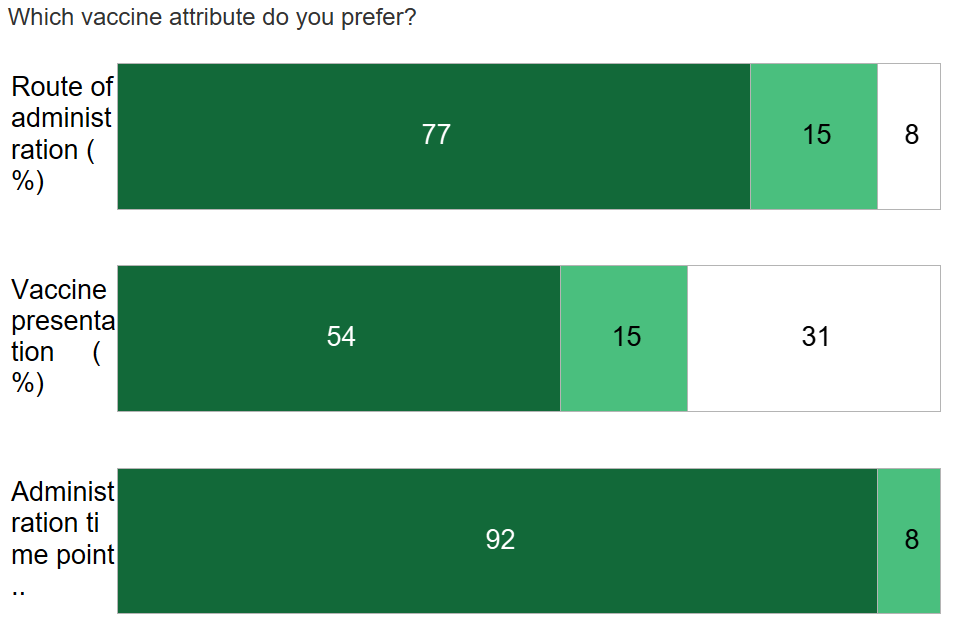

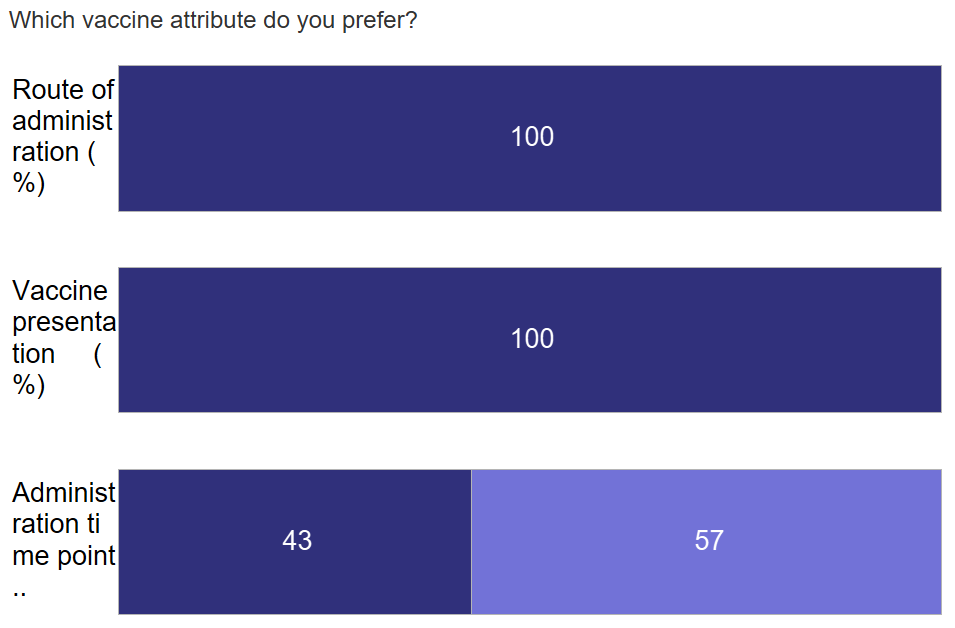


Route of administration (%)

Vaccine presentation (%)

Administration time point (%)

Route of administration (%)

Vaccine presentation (%)

Administration time point (%)

Healthcare providers (HP)

Which vaccine attribute do you prefer?


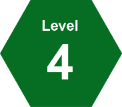


National stakeholders (NS)

Which vaccine attribute do you prefer?


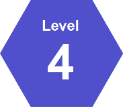


Ghana (NS n=6; HP n=11)


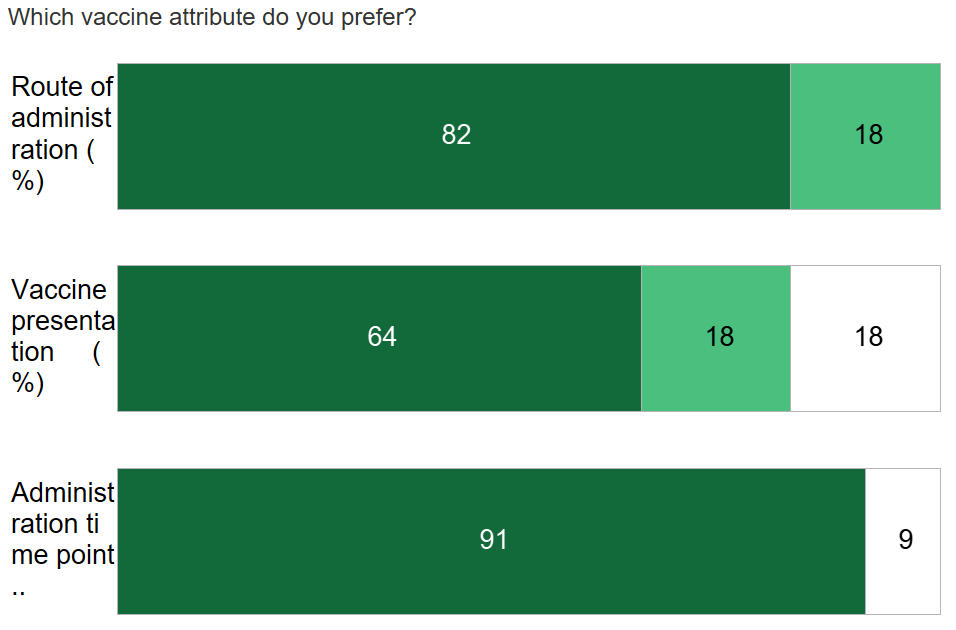

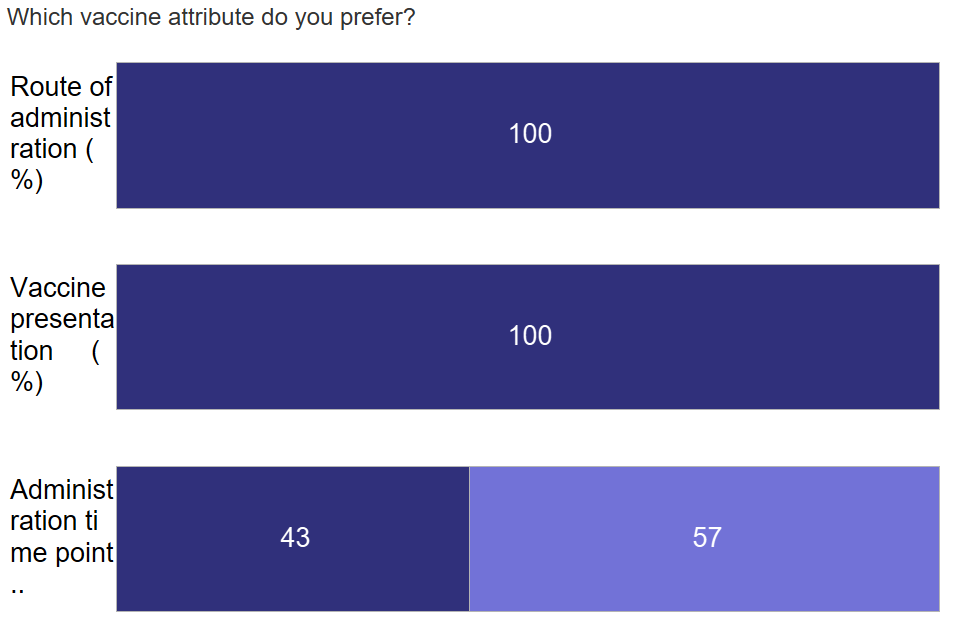


Route of administration (%)

Vaccine presentation (%)

Administration time point (%)

Route of administration (%)

Vaccine presentation (%)

Administration time point (%)

Kenya (NS n=5; HP n=10)


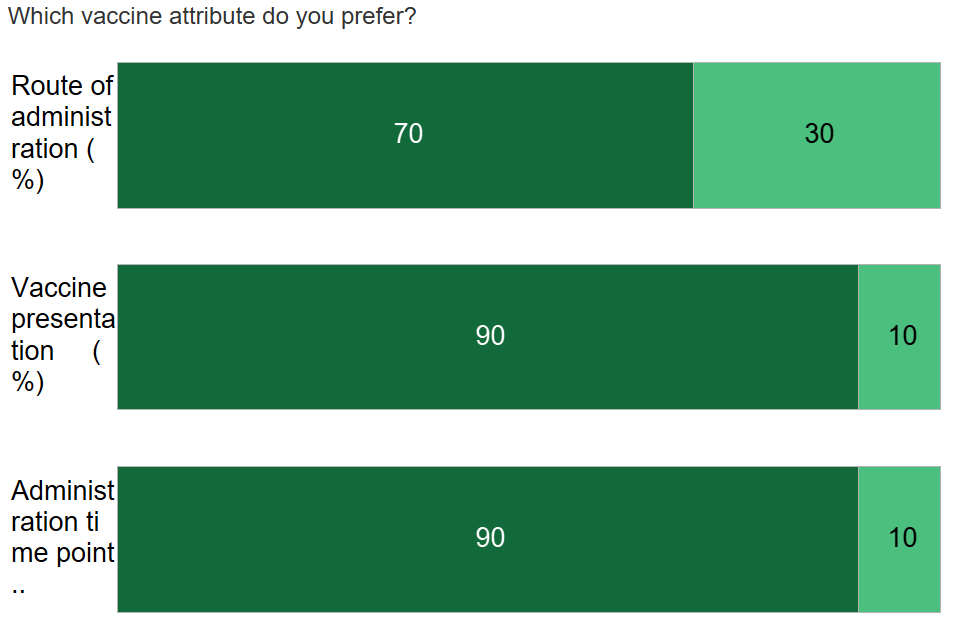

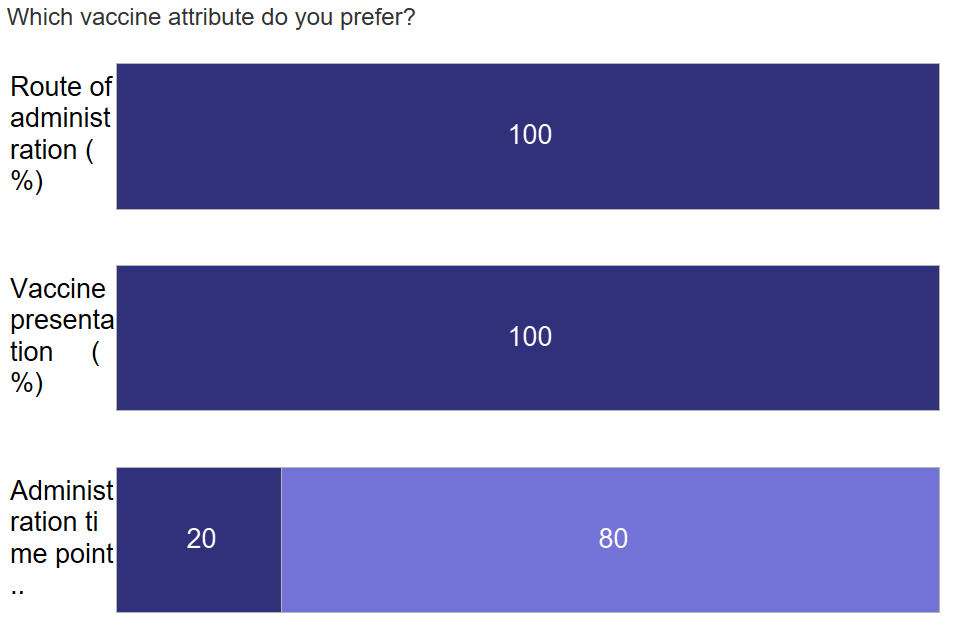


Route of administration (%)

Vaccine presentation (%)

Administration time point (%)

Route of administration (%)

Vaccine presentation (%)

Administration time point (%)

Nepal (NS n=5; HP n=10)


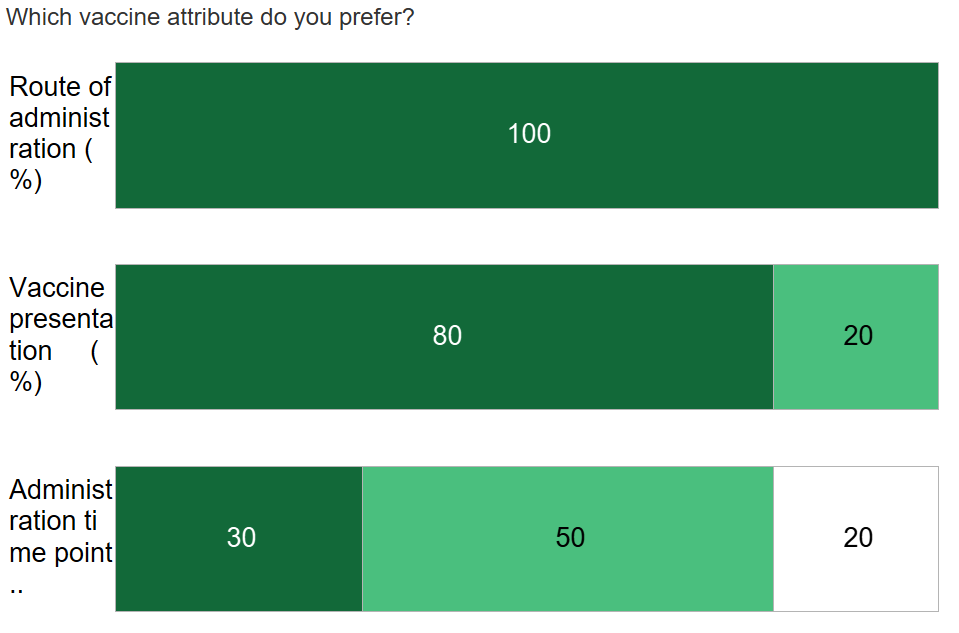

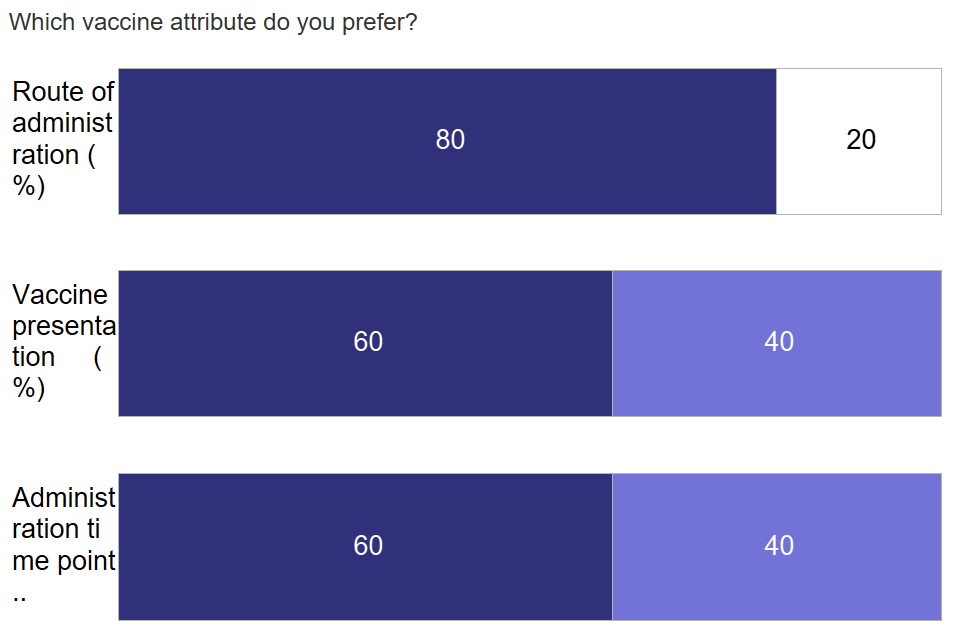


Route of administration (%)

Vaccine presentation (%)

Administration time point (%)

Route of administration (%)

Vaccine presentation (%)

Administration time point (%)

Vietnam (NS n=9; HP n=10)


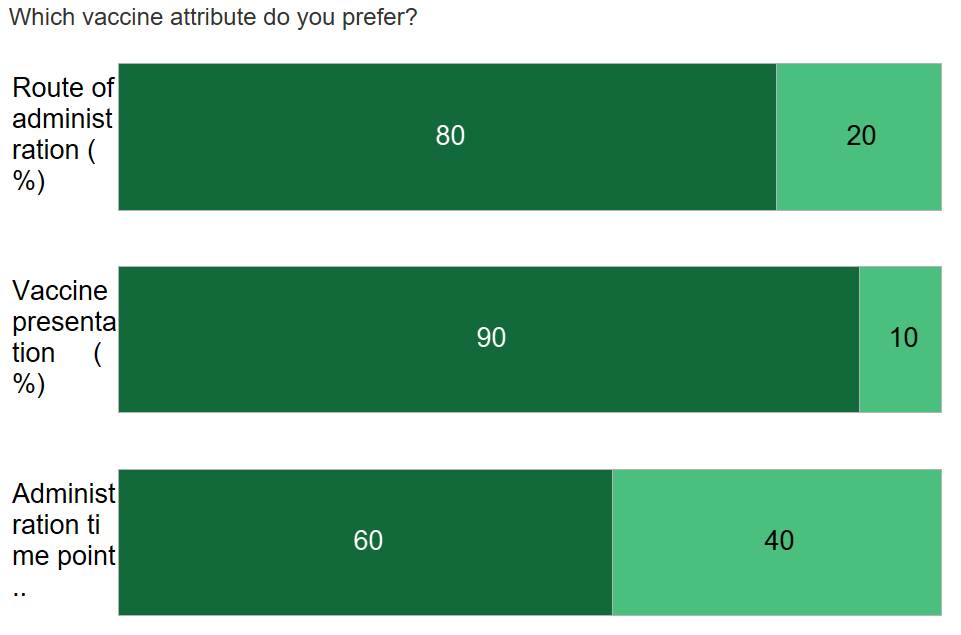

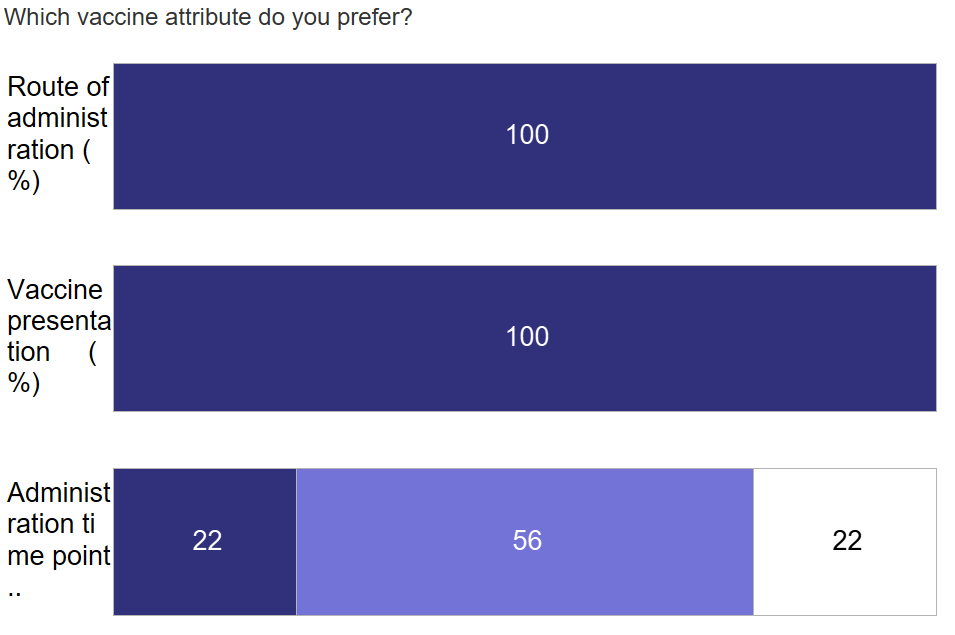


Route of administration (%)

Vaccine presentation (%)

Administration time point (%)

Route of administration (%)

Vaccine presentation (%)

Administration time point (%)


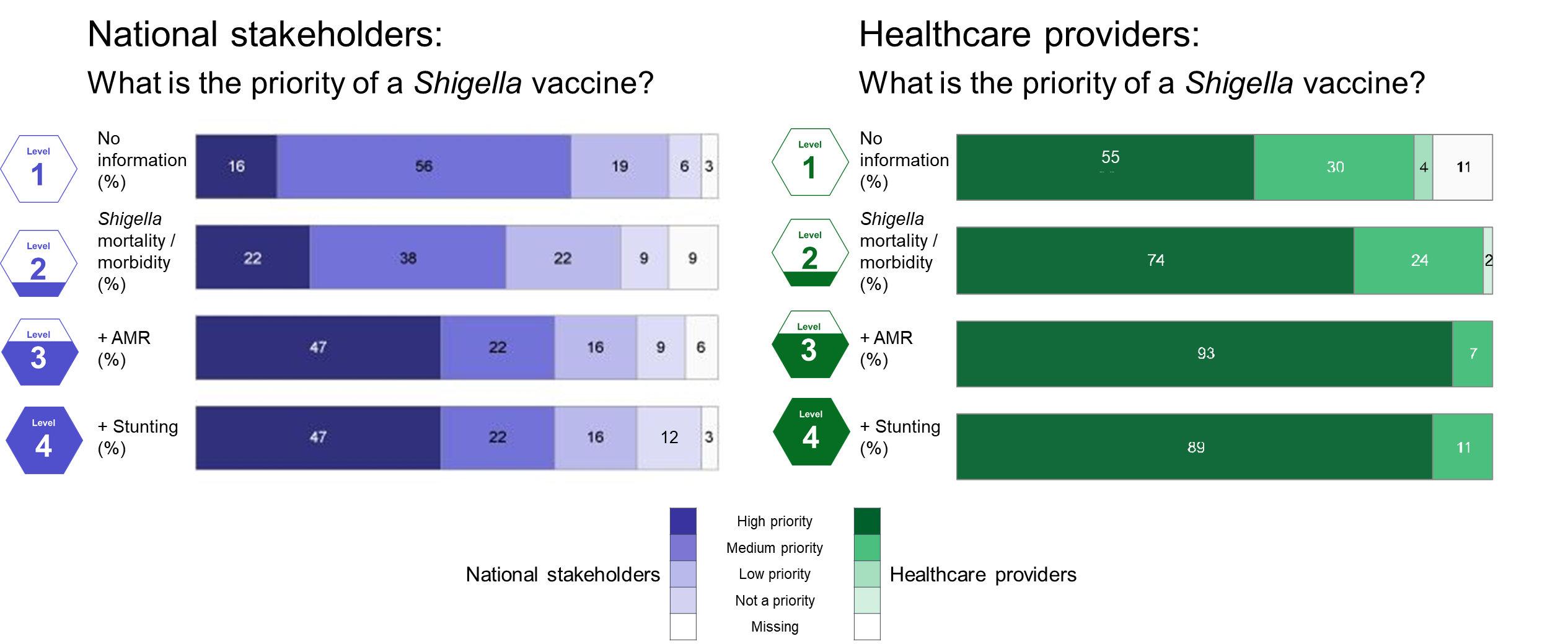

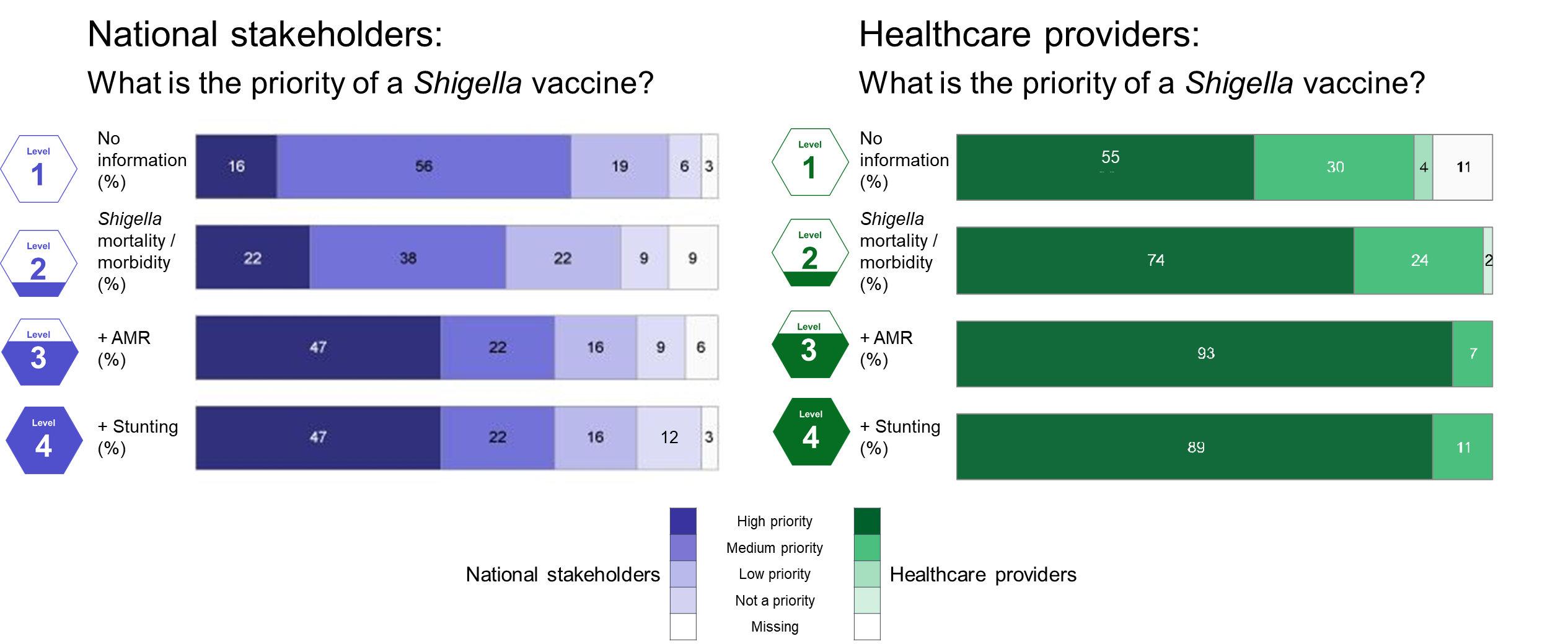


Oral

Injectable

No preference


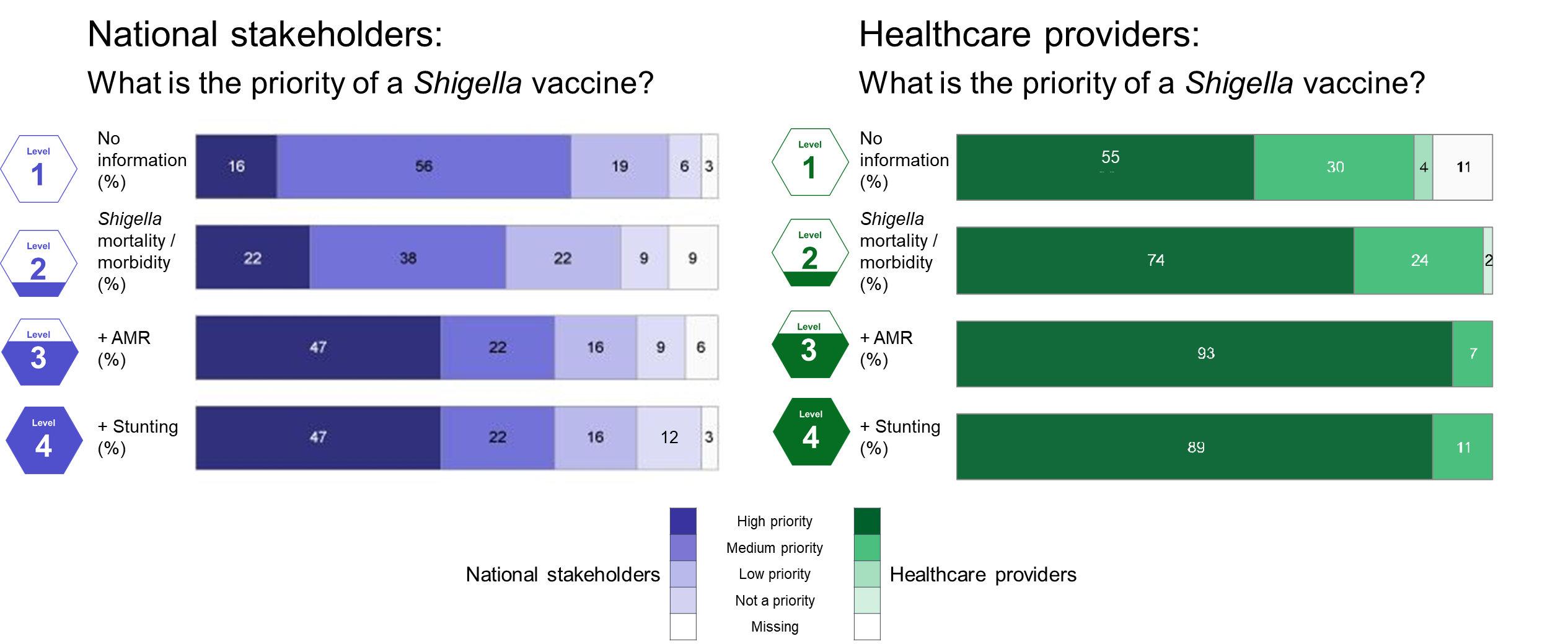

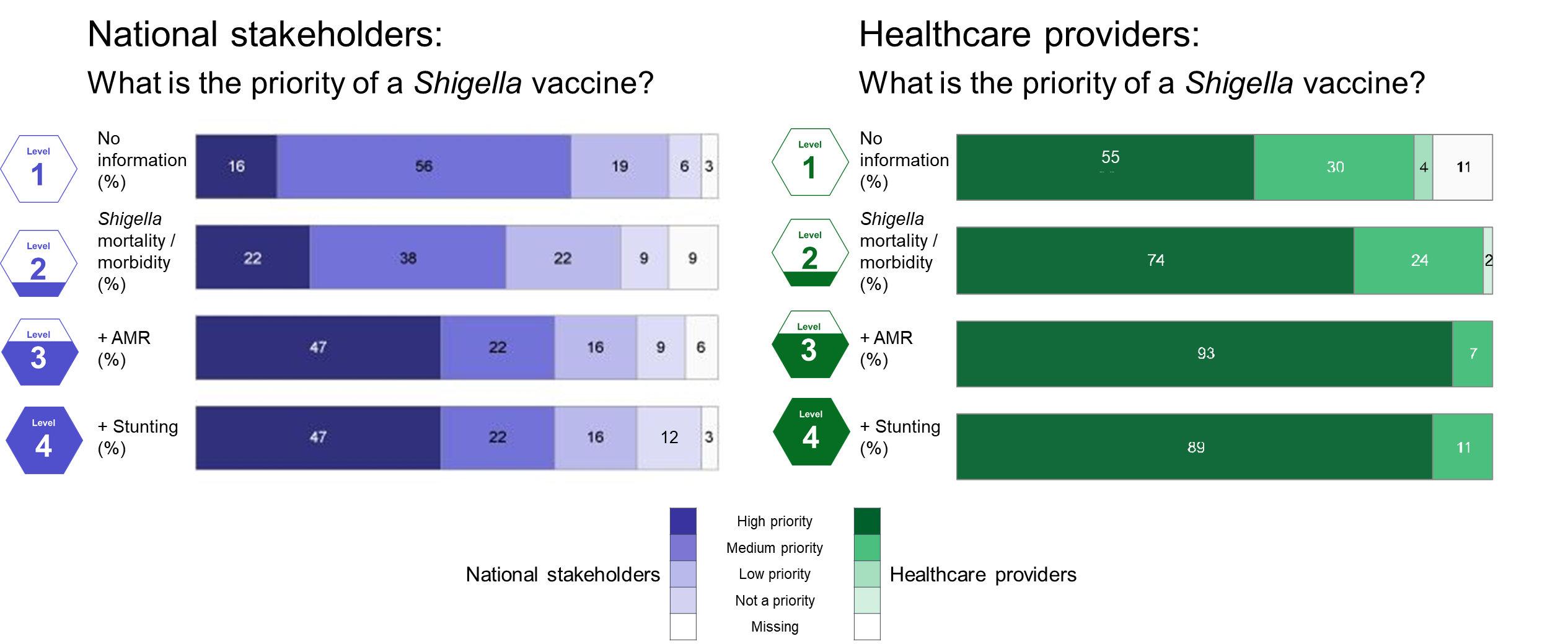


Combination

Single antigen

No preference


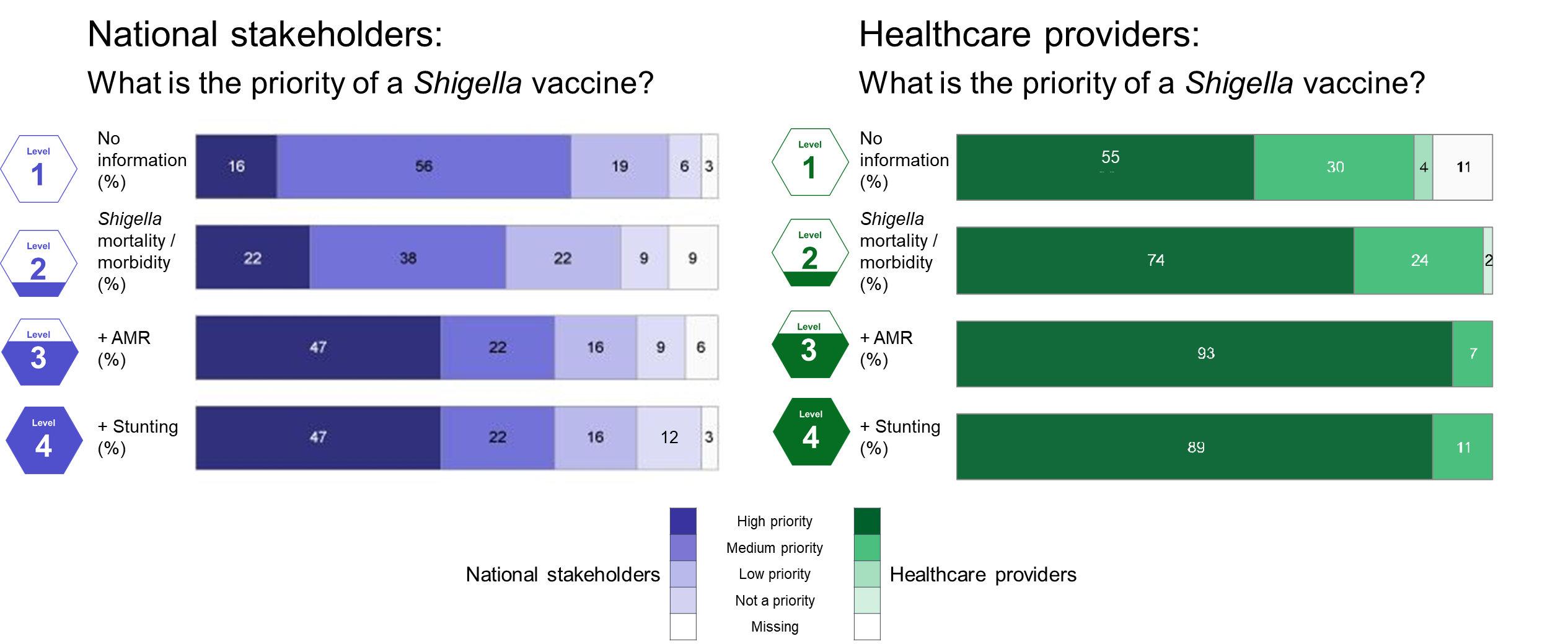

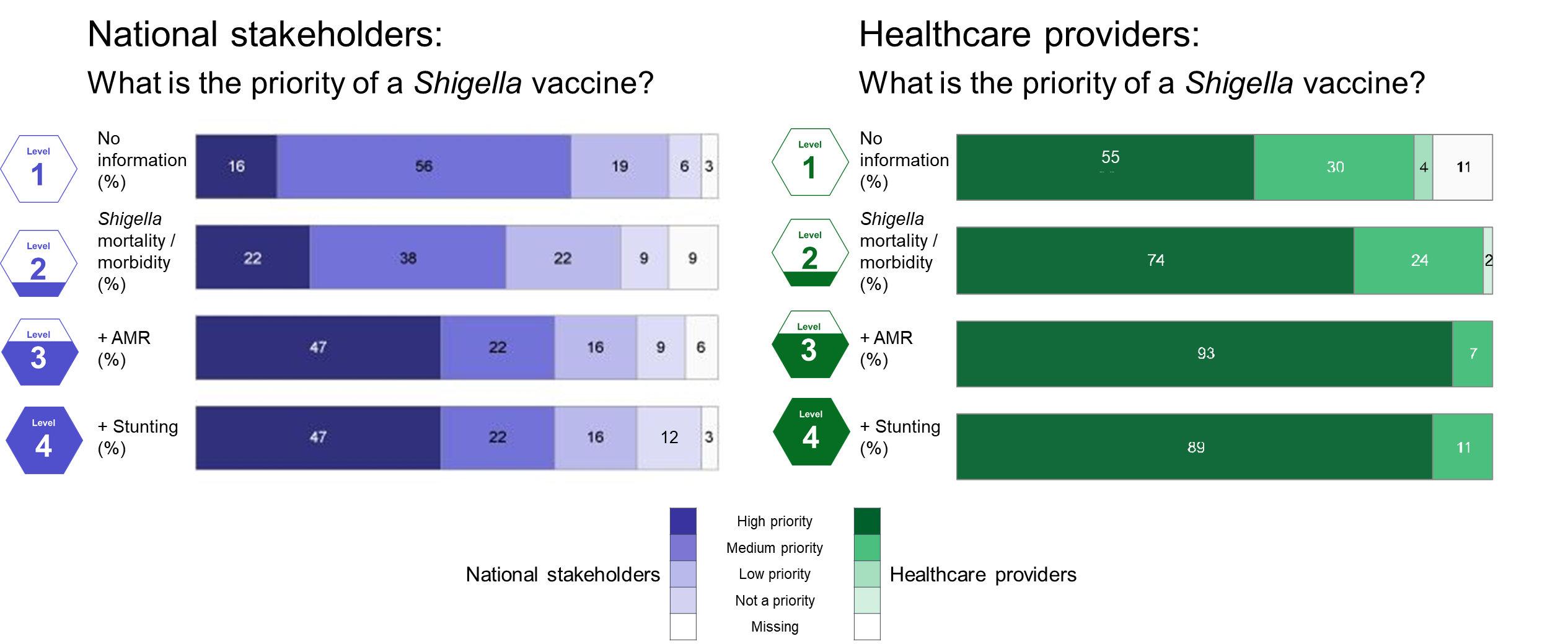


New vaccine visit

Existing vaccine visit

No preference


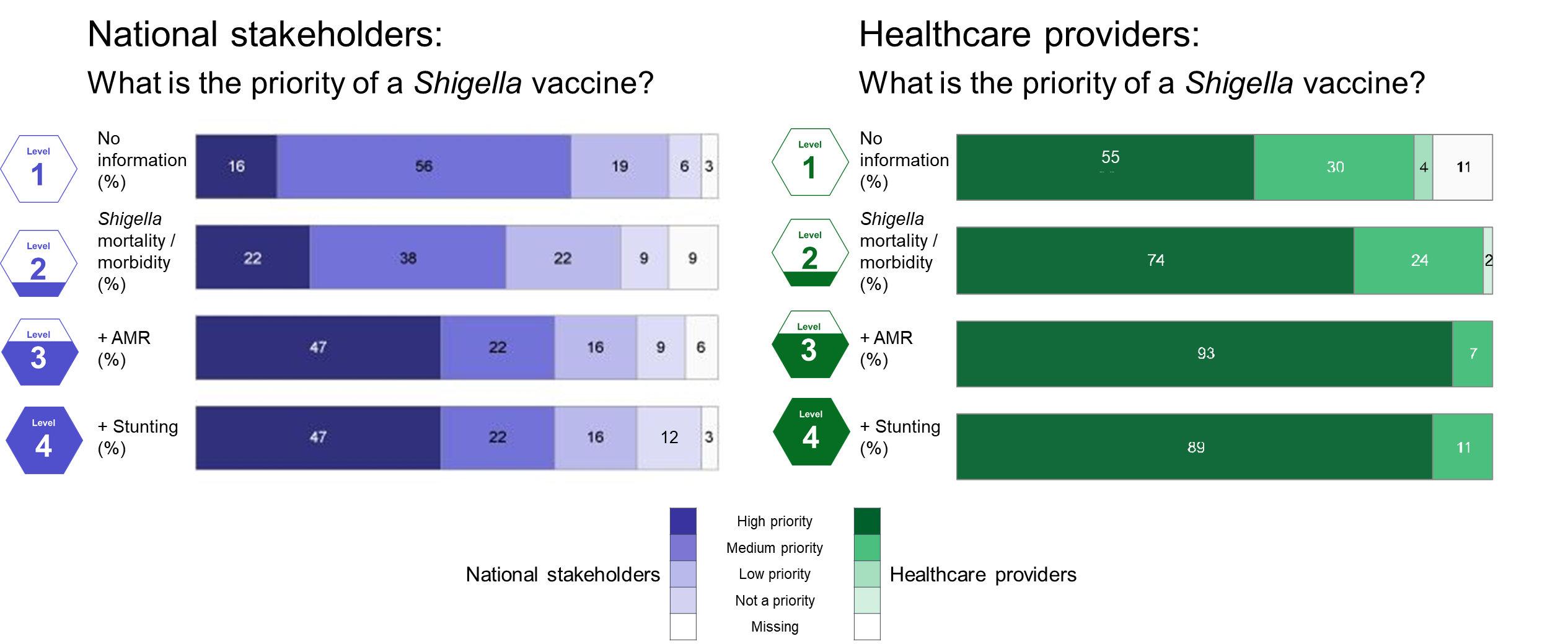

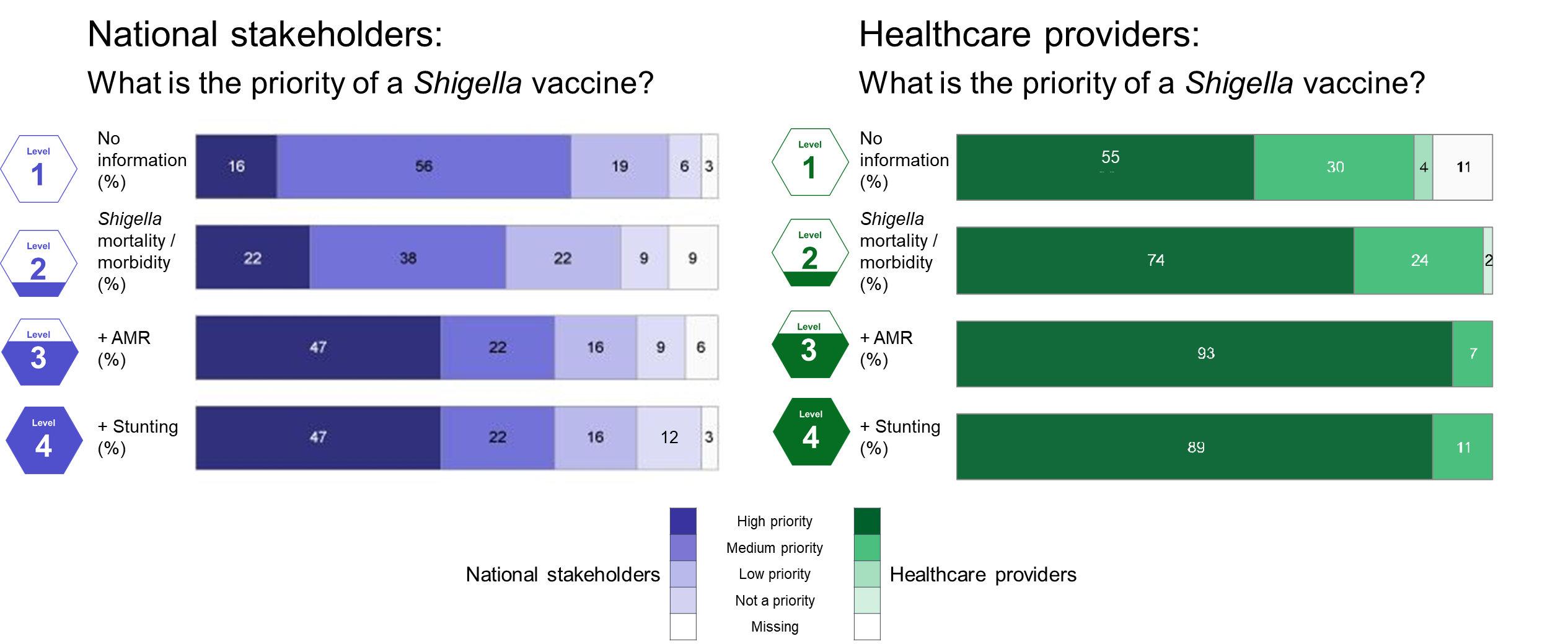


Oral

Injectable

No preference


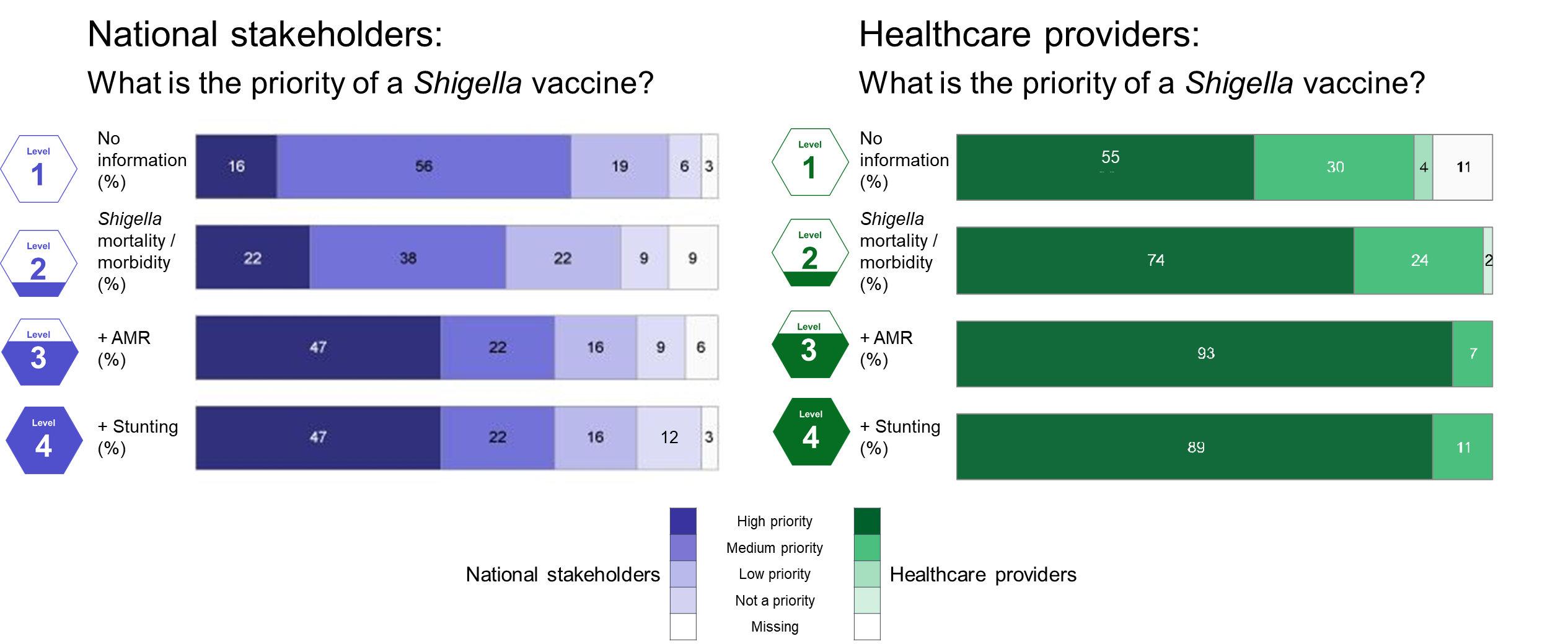

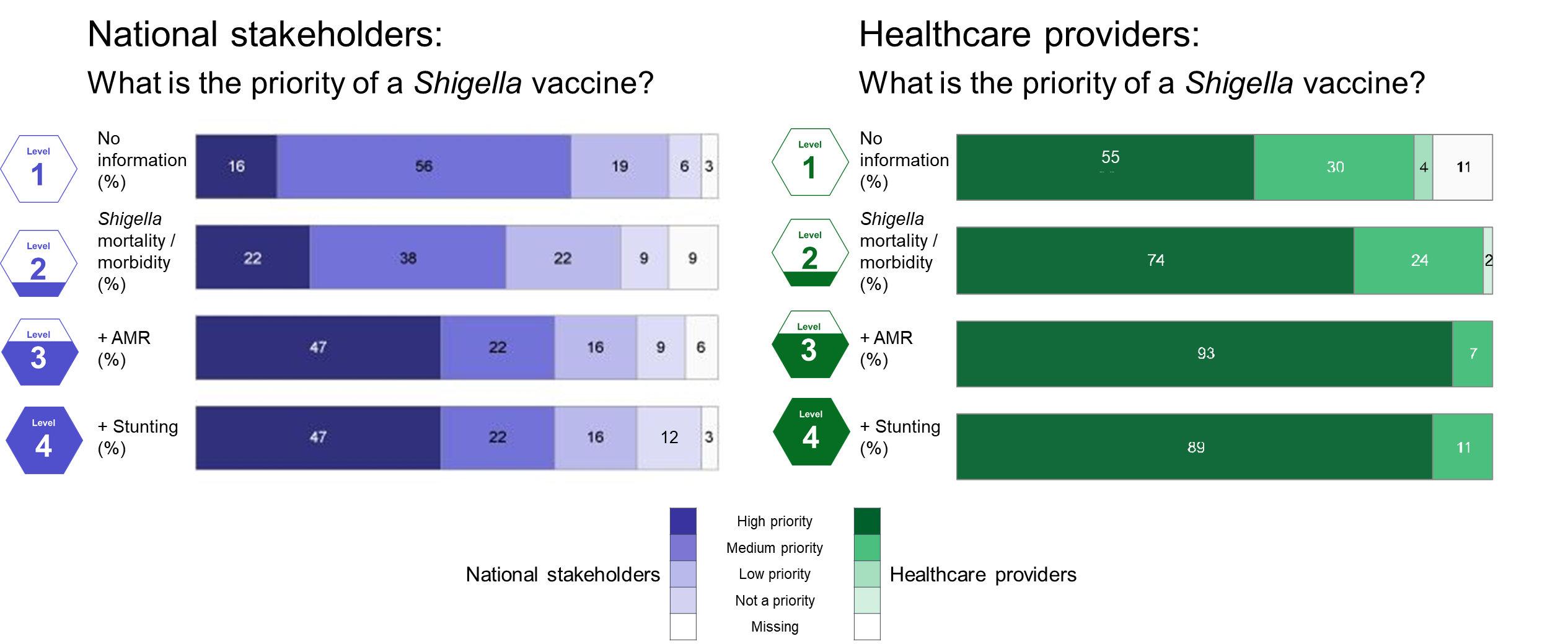


Combination

Single antigen

No preference


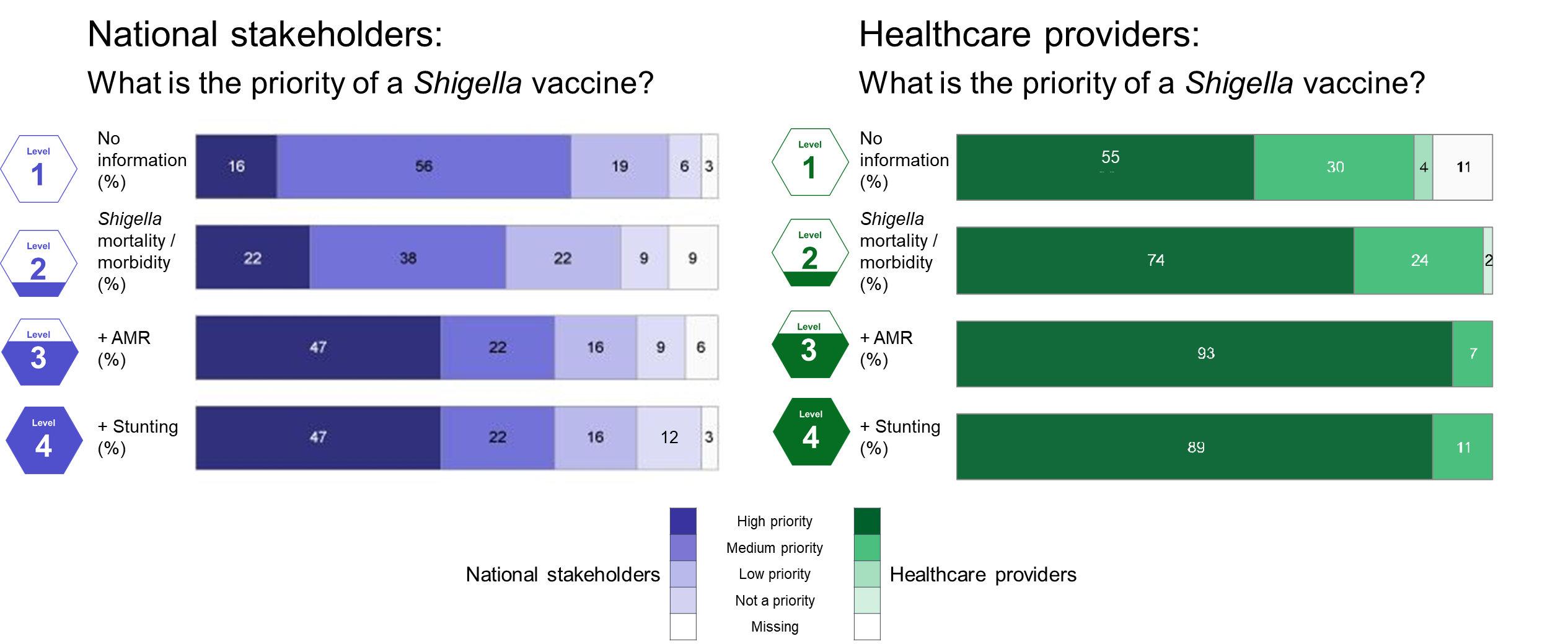

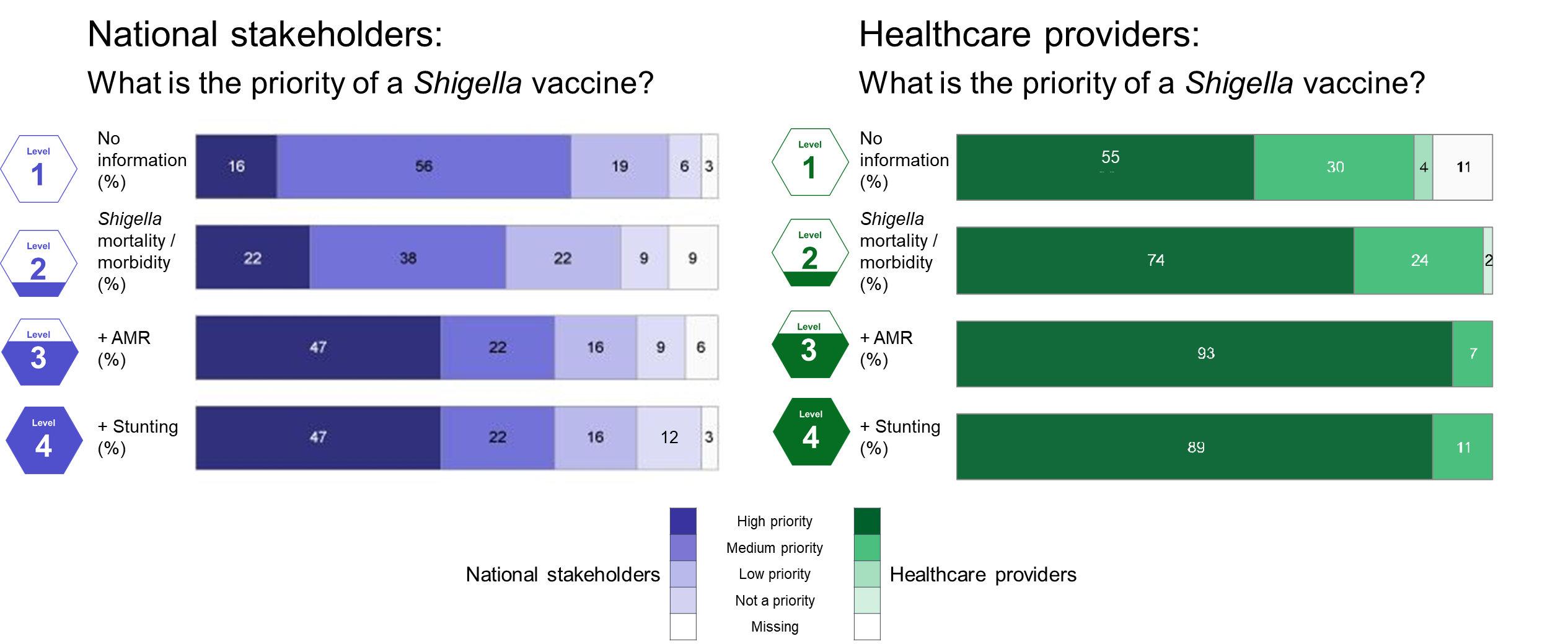


New vaccine visit

Existing vaccine visit

No preference

Administration time point

Vaccine

presentation

Route of administration
